# Characterization of a Putrescine Transaminase From *Pseudomonas putida* and its Application to the Synthesis of Benzylamine Derivatives

**DOI:** 10.3389/fbioe.2018.00205

**Published:** 2018-12-21

**Authors:** James L. Galman, Deepankar Gahloth, Fabio Parmeggiani, Iustina Slabu, David Leys, Nicholas J. Turner

**Affiliations:** School of Chemistry, Manchester Institute of Biotechnology, The University of Manchester, Manchester, United Kingdom

**Keywords:** biocatalysis, transaminase, green chemistry, benzylamines, protein structure

## Abstract

The reductive amination of prochiral ketones using biocatalysts has been of great interest to the pharmaceutical industry in the last decade for integrating novel strategies in the production of chiral building blocks with the intent of minimizing impact on the environment. Amongst the enzymes able to catalyze the direct amination of prochiral ketones, pyridoxal 5′-phosphate (PLP) dependent ω-transaminases have shown great promise as versatile industrial biocatalysts with high selectivity, regioselectivity, and broad substrate scope. Herein the biochemical characterization of a putrescine transaminase from *Pseudomonas putida* (Pp-SpuC) was performed, which showed an optimum pH and temperature of 8.0 and 60°C, respectively. To gain further structural insight of this enzyme, we crystallized the protein in the apo form and determined the structure to 2.1 Å resolution which revealed a dimer that adopts a class I transaminase fold comparable to other class III transaminases. Furthermore we exploited its dual substrate recognition for biogenic diamines (i.e., cadaverine) and readily available monoamines (i.e., isopropylamine) for the synthesis of benzylamine derivatives with excellent product conversions and extremely broad substrate tolerance.

## Introduction

**Figure 1:**
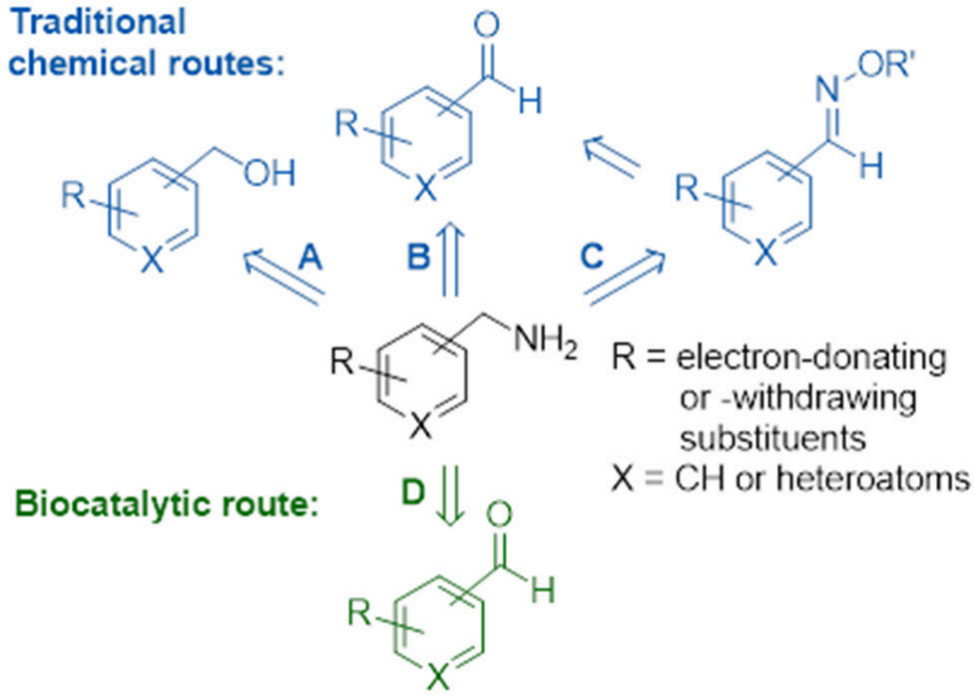
Chemical and biocatalytic routes to substituted benzylamines. **(A)** Direct amination of alcohols. **(B)** Reductive amination of aldehydes. **(C)** Reduction of oxime ethers. **(D)** Biocatalytic transamination.

Substituted benzylamines are essential building blocks for the synthesis of a diverse range of agrochemicals, pharmaceuticals, chiral ligands, and polymers (Roughley and Jordan, 2011; Froidevaux et al., 2016). Furthermore, they are key components with potent bioactivities in natural products and synthetic drugs, such as the topical antifungal treatment butenafine (Krauss et al., 2017), glucocorticoid receptor modulators with potent anti-inflammatory and immunosuppressive activity (Link et al., 2005), epoxide hydrolase inhibitors employed in the treatment of type II diabetes (Shen and Hammock, 2012), and most recently anti-tumor drugs (Mojena et al., 2018). The classical direct amination route, which has often been used on an industrial scale, is based on the reaction of alcohols with ammonia using transition metal supported catalysts (Figure 1A); however, this heterogeneous process requires high temperatures and pressures resulting sometimes in low yields of desired product (Hayes, 2001). For reasons of availability, a cost effective alternative would use a one-pot conversion of a carbonyl compound into an amine via the formation of an iminium ion intermediate which is subsequently reduced *in situ* by a reducing agent. Homogenous catalysis for the amination of carbonyl groups using rhodium, iridium, and ruthenium transition metal catalysts with ammonia under hydrogen pressure (Figure 1B) demonstrate this effective and clean procedure (Gross et al., 2002; Tan et al., 2018). Apart from transition metal catalysis, a well-known two-step approach involving the reaction of hydroxylamine with carbonyl groups to give oxime ethers and subsequent reduction with borane to yield amine products has also been applied (Figure 1C), but this cumbersome approach requires isolation and purification steps of the stable reaction intermediates (Feuer and Braunstein, 1969; Mirjafary et al., 2015).

Biocatalysis provides numerous advantages to the traditional chemical synthetic methods, allowing for biosustainable processes that exhibit excellent stereo- and regioselectivities, mild reaction conditions and no requirement for expensive and toxic heavy metals or protection-deprotection strategies. The expanding toolbox of enzymes enables scalable asymmetric amine synthesis to access chiral amine derivatives. Several biocatalytic strategies that have been developed include amine dehydrogenases (Ye et al., 2015), ammonia lyases (Parmeggiani et al., 2018), reductive aminases (Aleku et al., 2017) or via kinetic resolutions using monoamine oxidases (Ghislieri et al., 2013; Batista et al., 2018), and/or hydrolases (Reetz and Schimossek, 1996). Of these the reductive amination of prochiral ketones has been listed in the ACS Green Chemistry Roundtable discussion of implementing an atom efficient and environmentally benign method to produce chiral amines (Constable et al., 2007). The direct amination of prochiral ketones via the transfer of ammonia from an amine donor using ω-transaminases (Figure 1D) has an attractive advantage over dehydrogenases as the latter require an external nicotinamide cofactor regeneration system whilst ω-transaminases utilize pyridoxal 5′-phosphate (PLP) as a tightly bound prosthetic group (Slabu et al., 2017). However, ω-transaminase suffers from thermodynamic equilibrium limitations (Tufvesson et al., 2014) and substrate inhibition (Shin and Kim, 1997). Studies have shown this can be circumvented using inexpensive amine donors in large excess such as isopropylamine (which in the formation of volatile acetone that drives the equilibrium to the desired product) or using sacrificial amine cosubstrates which displaces equilibria via ring aromatization or cyclisation (Gomm et al., 2016; Martínez-Montero et al., 2016; Galman et al., 2017). To date there are many examples that have demonstrated the use of ω-transaminases toward the production of industrially relevant amines, alkaloids, and other bioactive natural products (Guo and Berglund, 2017; Costa et al., 2018; Galman et al., 2018).

We earlier identified a putrescine transaminase *spuC* gene in *Pseudomonas putida* with preference to aliphatic diamines other than putrescine such as cadaverine and spermidine (Galman et al., 2017). A basic local alignment search tool (BLAST) analysis (Altschul et al., 1990) revealed a 76% protein sequence identity to a *spuC* gene from *Pseudomonas aeruginosa*, that was previously characterized to be part of a polyamine uptake and utilization pathway converting putrescine to 4-aminobutyraldehyde (Lu et al., 2002). Further investigations supported the physiological role of SpuC by maintaining alanine homeostasis via the transamination of pyruvate (Han et al., 2008). However, the promiscuous role of *P. putida* Pp-SpuC may also contribute to a complementary pathway in L-lysine catabolism via the cadaverine route (Revelles et al., 2005) to produce sought after polyamide building blocks 5-aminovalerate and glutaric acid (Adkins et al., 2013). In this study, we report on the detailed characterization (structural and biochemical) of the putrescine transaminase Pp-SpuC, and its application in the transamination of a comprehensive range of ketones/aldehydes for the production of a variety of benzylamine derivatives.

## Results

### The Effects of Temperature and pH on Pp-SpuC

**Figure 2:**
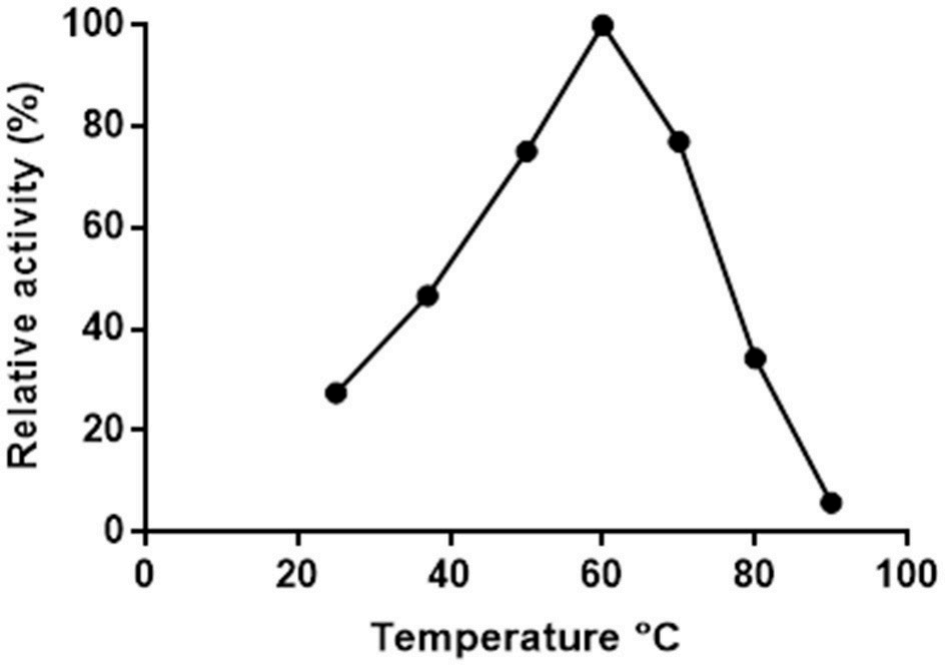
Effect of temperature on the activity of Pp-SpuC. The activity was determined spectrophotometrically based the formation of acetophenone.

Putrescine transaminase Pp-SpuC was cloned into a pET28b vector containing an *N*-terminal His_6_-tag and recombinantly expressed in *E. coli* BL21 (DE3) as previously reported (Galman et al., 2017). The initial thermoactivity of purified Pp-SpuC was investigated by incubating the enzyme with (*S*)-α-methylbenzylamine and sodium pyruvate at 25–90°C in Tris-HCl buffer pH 8.0 (Figure 2). The activity was measured according to the UV absorbance change due to the formation of acetophenone. The activity of the enzyme significantly improved as the temperature increased to its suboptimum activity at 50°C, and reached 100% optimum relative activity at 60°C. At higher temperature, it showed gradual denaturation but maintaining significant residual activity (77%) up to 70°C. Interestingly, an activity above 50% of the maximum was observed over a large temperature range (45–75°C).

**Figure 3:**
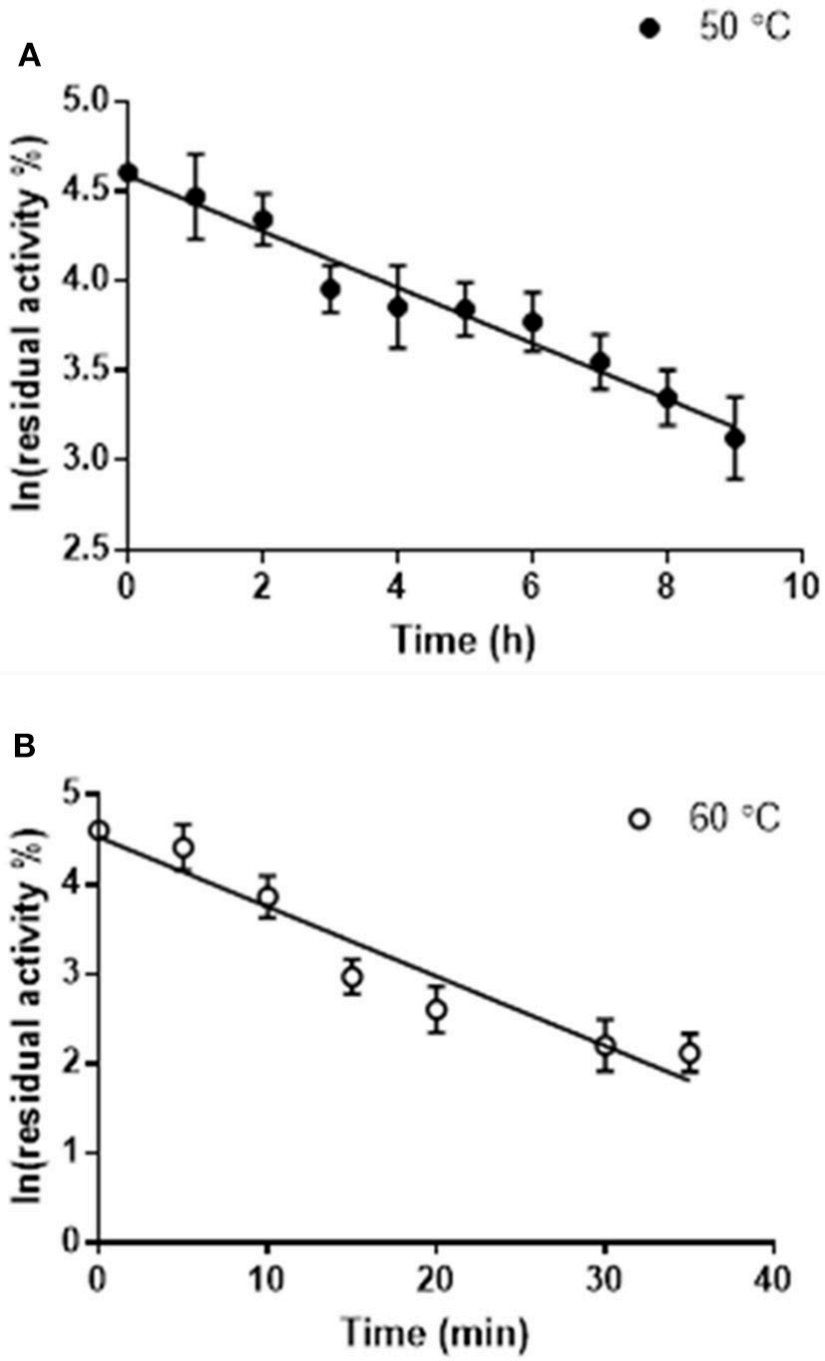
Residual activity of purified Pp-SpuC after incubation in 50 mM Tris-HCl, 0.1 mM PLP at different temperatures. **(A)** Incubation at 50°C (●) with a half life t_1/2_ = 7.7 min. **(B)** Incubation at 60°C (○) with a half life t_1/2_ = 4.7 h. All reactions were performed in triplicate and the standard deviations <9%.

The thermostability of Pp-SpuC was assayed by incubating the enzyme (5 mg mL^−1^) at optimum and sub-optimum temperatures for different times, then assaying the residual activity at 30°C as described above (Figure 3). Each result was an average of triplicate measurements from the initial rates upon the formation of acetophenone and was linearly plotted as ln(residual activity %) vs. time. The deactivation rate constants *k*_d_ were calculated at 4.65 and 0.156 h^−1^ for incubated enzyme temperatures at 60 and 50°C, respectively. The half-life times (t_1/2_ = ln2/k_d_) were calculated as 4.4 h at 50°C which is more stable than the optimal relative activity conditions at 60°C with a half-life time of 8.9 min.

**Figure 4:**
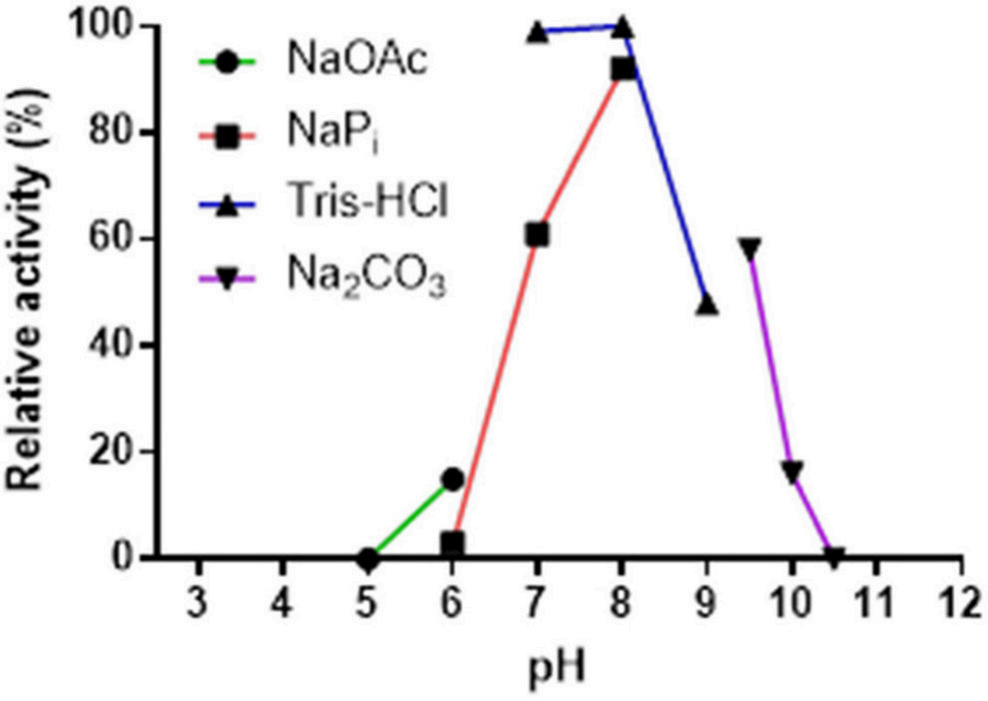
Effect of pH on the activity of Pp-SpuC. The activity was determined by HPLC based on the formation of the corresponding amine. The buffers used were acetate (pH 5.0–6.0), phosphate (pH 6.0–8.0), Tris-HCl (pH 7.0–9.0), and carbonate (pH 9.5–10.5) at a concentration of 50 mM.

The pH-stability profile of Pp-SpuC was determined by incubating at 30°C for 24 h the purified enzyme in different buffer solutions (50 mM), then measuring residual activity as described above (Figure 4). The pH profile reveals the effect on transaminase activity in defined buffers shown as relative activity using 2-fluoroacetophenone as the acceptor substrate and isopropylamine (50 equiv.) as the amine donor. Pp-SpuC was found inactive at acidic pH values pH < 6.0 and active at pH 7.0–9.0 with optimum activity at pH 8.0, which is typical of a majority of (*S*)-selective transaminases such as those from *Halomonas elongata* He-ωTA (Cerioli et al., 2015), *Vibrio fluvialis* Vf-ωTA (Shin et al., 2003), and *Chromobacterium violaceum* Cv-ωTA (Schell et al., 2009). Tris-HCl buffer was found to stabilize the protein, with the highest activity at pH 7.0 (compared to phosphate buffer which showed a 40% decrease in activity at the same pH). The decrease in activity is likely to be due to the displacement of the cofactor pyridoxamine-5′-phosphate (PMP), the aminated form of PLP, via competing phosphate ions, as previously reported within Class III transaminases (Schell et al., 2009; Mathew et al., 2015). Enzyme stability decreases by about 55% when the pH was increased to 9.0 and only negligible activity could be seen at pH > 10.0.

### The Effects of the Nature and Concentration of Co-solvents on Pp-SpuC

**Figure 5:**
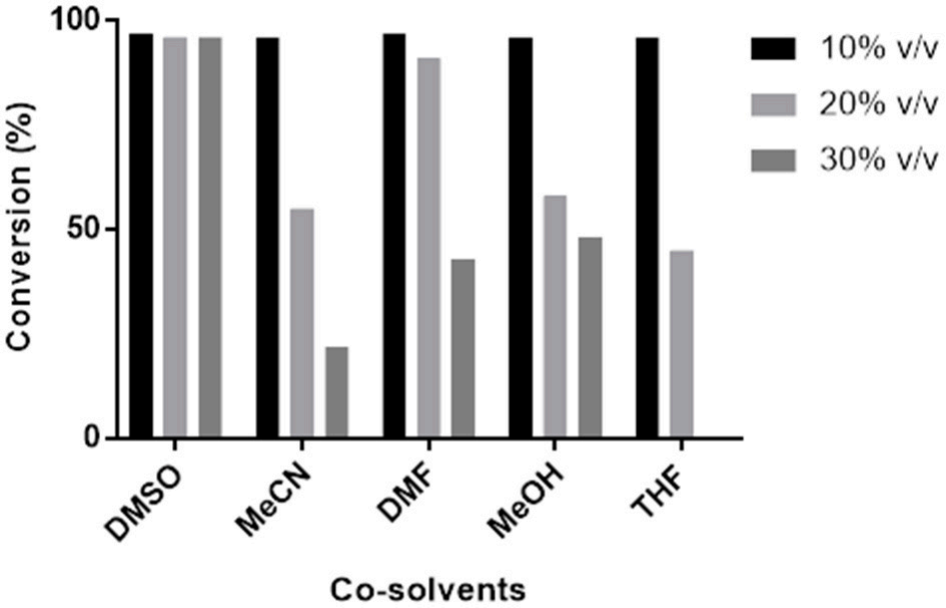
Effect of co-solvents on the activity of Pp-SpuC against **1a**. The activity was determined by LC-MS method based on the formation of the corresponding amine **1b**.

Transaminase catalyzed reactions often rely on the use of organic co-solvents, primarily to increase the solubility of hydrophobic substrates. Biotransformation reactions with Pp-SpuC transaminase were previously conducted using 1% ν/ν DMSO and gave high product conversions (Galman et al., 2017). However, a detailed study of the influence of various organic co-solvents was not performed (Figure 5). In order to investigate the effects, Pp-SpuC reactions were performed on a 5 mM scale using 3-fluorobenzaldehyde **1a** as the substrate acceptor with 50 equivalents of IPA in the presence of 10–30% ν/ν of several common co-solvents. Pp-SpuC was shown to tolerate up to 10% ν/ν with all the organic co-solvents tested. Increasing the co-solvents to 20% ν/ν revealed a 45–55% decrease in activity with MeCN, MeOH, and THF. Interestingly, the activity of Pp-SpuC remained unaffected with DMSO at 30% ν/ν, therefore higher concentrations were tested, revealing almost identical activity at up to 40% ν/ν DMSO. The high DMSO stability of Pp-SpuC is comparable to a recently published wild type transaminase (pQR2189, 52% protein sequence identity to Pp-SpuC) isolated from a household drain metagenome, displaying activity up to 50% ν/ν DMSO (Leipold et al., in press).

### Crystallization and Structure Determination of Pp-SpuC

In an attempt to rationalize the substrate scope and gain better insight into the catalytic mechanism, the crystal structure of Pp-SpuC was solved as follows. The His-tagged Pp-SpuC purified by nickel affinity chromatography was further purified by ion-exchange before crystallization. A commercially available crystallogenesis screening set was employed, obtaining crystals under a number of different conditions after overnight incubation at 20°C. The best diffracting crystals were obtained in 0.2 M MgCl_2_, 0.1 M Bis-Tris pH 6.5, 25% PEG3350, then cryoprotected before freezing. X-ray diffraction data was collected from single crystal at IO4 beamline at the Diamond Light Source Syncrotron (UK). The structure was solved by the molecular replacement method using as a search model the aspartate aminotransferase from *Pseudomonas* sp. (Pse-ωTA) (PDB id: 5TI8) (Wilding et al., 2017) which shows 77% identity with Pp-SpuC. The final refined structure of Pp-SpuC has been deposited in the PDB database (PDB id: 6HX9).

### Applications of Pp-SpuC as a Biocatalyst With Broad Substrate Tolerance

Lastly, to demonstrate the applicability of Pp-SpuC as an efficient biocatalyst, the transamination of a broad range of aromatic carbonyl compounds (a representative panel of 30 aldehyde/ketone substrates substituted with halogens, electron-donating groups, and electron-withdrawing groups, was tested. Reactions were carried out with recombinant Pp-SpuC (2 mg mL^−1^) in Tris-HCl buffer (50 mM) pH 9.0, containing PLP (1.0 mM), at 30°C for 18 h. For comparison, two equilibrium-shifting approaches were tested: either a large excess isopropylamine (50 equiv.) as a low-cost amine donor, or bio-derived cadaverine (5 equiv.) as a cleaner, “green” alternative sacrificial co-substrate, which drives the reaction by spontaneous cyclisation. Conversions were monitored by LC-MS and the identity of the products was verified by MS and/or by comparison with authentic standards. In most cases, almost quantitative conversions could be observed (see Discussion section), proving the broad substrate tolerance of this enzyme.

In order to demonstrate the feasibility of the reaction with reduced catalyst loadings, biotransformations of the model substrate **1a** were performed with successive ten-fold dilutions, under the same conditions specified above. With a catalyst concentration of 0.2 mg mL^−1^ full conversion could still be achieved, while reducing the enzyme loading to 0.02 mg mL^−1^ only a modest conversion (19%) was observed. From the latter data, with the substrate only partially consumed, a total turnover number (TTN) could be estimated to ~2400.

## Discussion

### X-ray Crystal Structure of Pp-SpuC

**Figure 6:**
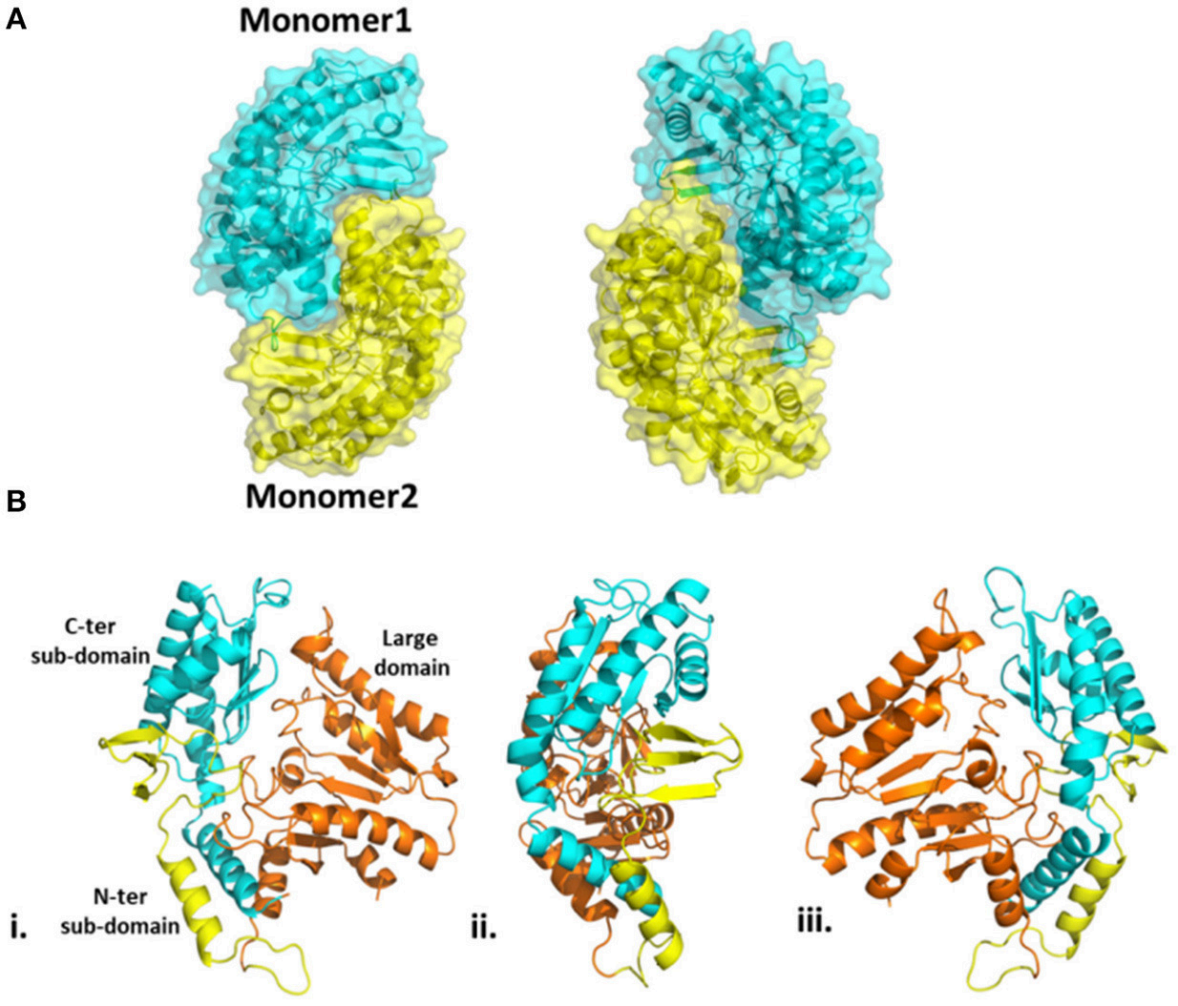
**(A)** Homodimeric structure of Pp-SpuC. Monomer 1 shown in cyan and monomer 2 shown in yellow. **(B)** Domain organization of the Pp-SpuC monomer, divided into one large domain (residue 93–313, orange) and two sub-domains, N-terminal (residues 1–92, yellow) and C-terminal (residues 314–352, cyan). Views (ii) and (iii) are obtained by 90° rotation of (i).

The overall structure of Pp-SpuC (PDB id: 6HX9) is similar to *Pseudomonas* sp. Pse-ωTA (PDB id: 5TI8) (Wilding et al., 2017) and *C. violaceum* Cv-ωTA (PDB id: 4A6T) (Humble et al., 2012; Sayer et al., 2013) and typical to the class I transaminase fold. There are two monomers in the asymmetric unit and the enzyme forms a tight dimer with predicted surface area of about 28710 Å^2^, with buried surface area of 3980 Å${}_{.}^{2}$ Each monomer can be divided into two major domains (Figure 6). The large domain comprises residues 93–313 and folds into a sandwich of α/β/α comprising a core of seven stranded β-sheets with only β10 being antiparallel. The large domain is flanked by a small domain composed of two subdomains joined by a parallel β interaction between strand β3 and β14. The *N*-terminal subdomain is composed of three antiparallel β strands. In the case of holo Cv-ωTA (PDB id: 4A6U) these anti parallel β strands capped by two most *N*-terminal α-helices, which were absent in our structure, similarly to the apo structures of CV-ωTA (PDB id: 4A6U) and Pse-ωTA (PDB id: 5TI8). The *N*-terminal subdomain forms extensive interactions with monomer 2 of the dimer which is important for active site formation, similar to what was observed with Cv-ωTA and Pse-ωTA. The *C*-terminal subdomain is formed of four antiparallel β-sheets.

**Figure 7:**
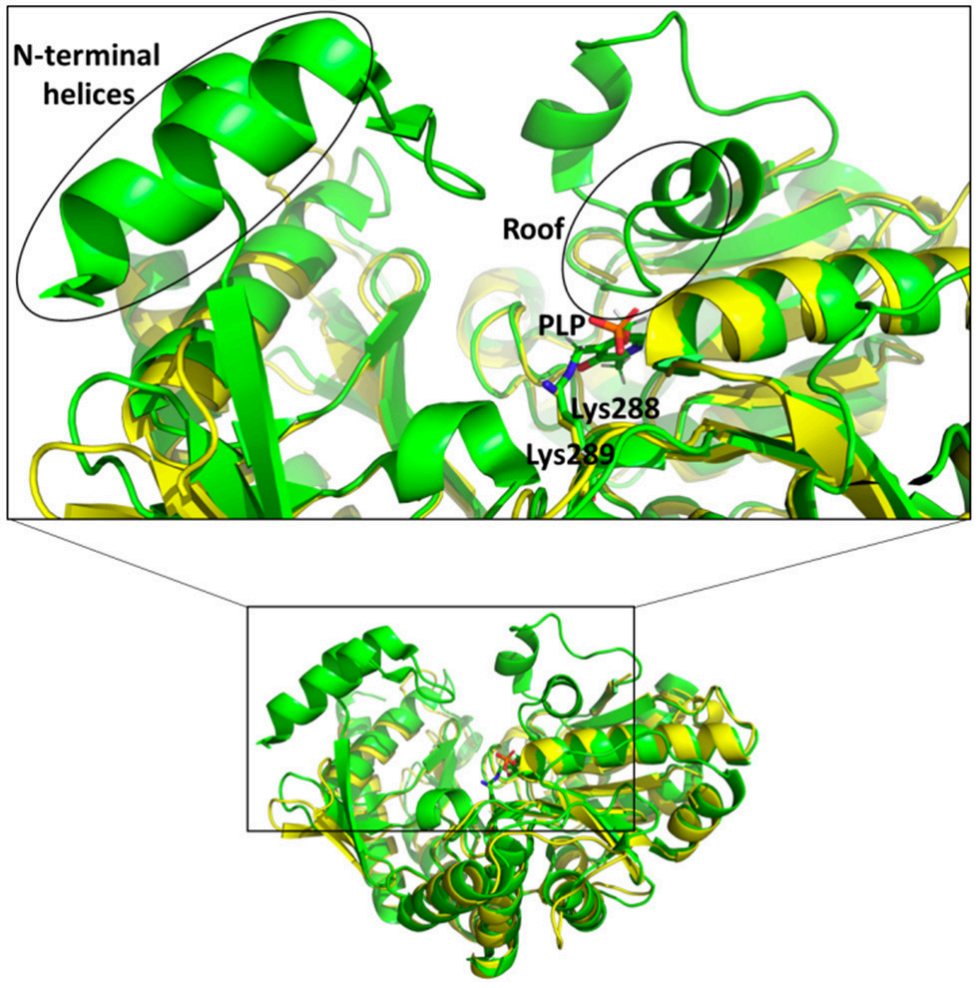
Superposition of holo Cv-ωTA (PDB id: 4A6T, green) structure on Pp-SpuC crystal structure (PDB id: 6HX9, yellow). The Pp-SpuC catalytic lysine (Lys289) residue superimposes well with the catalytic Lys288 of holo Cv-ωTA. In the case of Pp-SpuC the *N*-terminal helices and the residues corresponding to the roof of the PLP binding site (highlighted in the box) remain disordered.

In the active site of aminotransferases, the PLP cofactor binds to the enzyme to form an aldimine intermediate in which the C4′ of PLP forms a Schiff base with an active site lysine during the catalytic interaction (Eliot and Kirsch, 2004). Unfortunately, all our attempts to obtain the structure of the complex of Pp-SpuC with PLP bound were unsuccessful. The superposition of the Pp-SpuC structure with the holo structure of Cv-ωTA (PDB id: 4A6T) reveals the position of the active site. In the case of SpuC, the catalytic residue Lys289 is located on the loop between β4 and β10. The Pp-Spuc structure is very similar to the Pse-ωTA (PDB id: 5TI8) (Wilding et al., 2017) and the apo form of Cv-ωTA (PDB id: 4A6U) (Humble et al., 2012) as compared to holo structure of Cv-ωTA (PDB id: 4A6T). The first *N*-terminal 33 residues are missing from the pp-SpuC structure which forms the corresponding two α helices in the holo Cv-ωTA (PDB id: 4A6T). Another disordered region of Pp-SpuC are amino acid residues 154–179 and 313–324. The homologous region 154–179 forms a “roof” above the cofactor binding site in the holo structure of Cv-ωTA, indicating its importance in PLP binding (Figure 7). This region is also absent in case of apo-Cv-ωTA and Pse-ωTA. The additional missing loop from 313–324 which should connect two alpha helices and comes close to the phosphate moiety of the PLP cofactor in holo-Cv-ωTA (PDB id: 4A6T).

### Comparison of Pp-SpuC Active Site Structure and Catalytic Activity With Related Class III Transaminases

**Figure 8:**
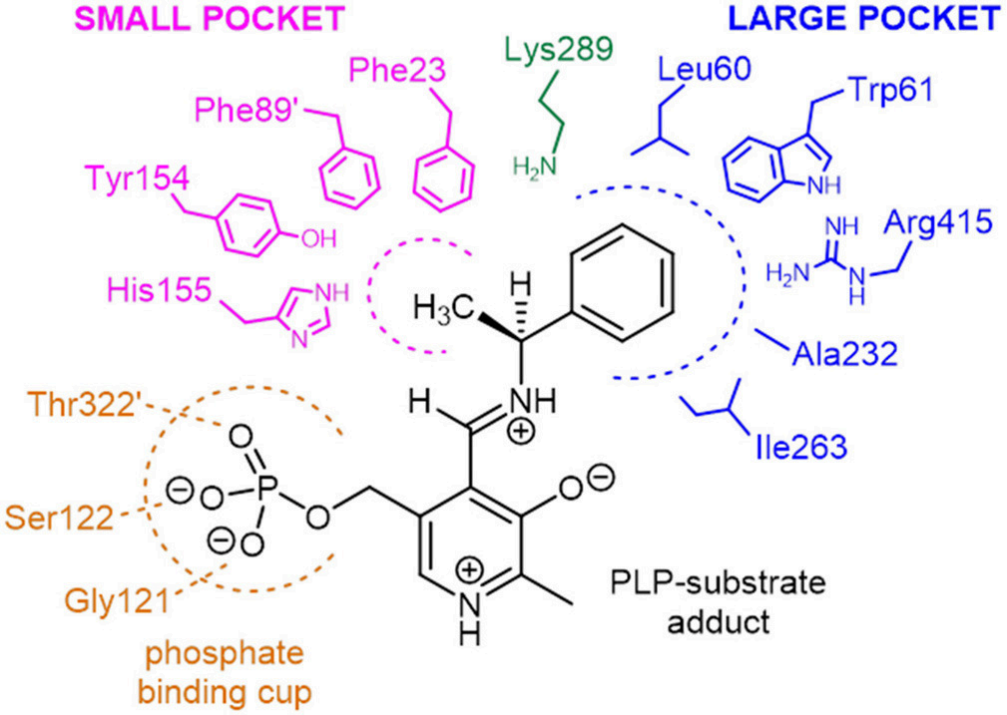
Schematic representation of the amino acid residues forming the small and large binding pockets of Pp-SpuC around the external aldimine intermediate (PLP-substrate adduct). The model is based on the multiple sequence alignment analysis and on the active site structure of Vf-ωTA (PDB id: 4E3Q).

Two structurally homologous genes Vf-ωTA (Shin and Kim, 2002; Midelfort et al., 2013) and Cv-ωTA with a sequence identity of 36 and 55% respectively have been extensively studied with crystals isolated in the apo and holo form co-crystallized with PLP or bound to a suicide inhibitor such as gabaculine (Sayer et al., 2013). Superposition of Pp-SpuC structure with the Cv-ωTA apo monomer (PDB id: 4A6U) (Humble et al., 2012), in which the authors also experienced difficulties obtaining crystals with PLP bound, gave an root mean square deviation (RMSD) value of 1.5 Å between 376 Cα atoms of aligned residues (out of 389) with the same “roof” residues missing in the apo structure of Cv-ωTA. The disordered region may indicate why no electron density is observed for the PLP cofactor at the interface of the two enzyme subunits. A structure-based sequence alignment of closely related ω-TAs was constructed with Pp-SpuC as a template, revealing several important characteristics for these industrially relevant enzymes for the production of chiral amines (Figure S1, Supplementary Information). Similar to other Class III transaminases of fold type I, the active site of ω-TAs contain two binding pockets for binding of the substrates (Figure 8). The active key amino acid residues which constitute the small binding pocket and allow accommodation of substituents less bulky than an ethyl group are identified in Pp-SpuC as Phe23, Phe89′ (from the other subunit), Tyr154 and His155 (absent in the Pp-SpuC apo structure). A clear observation of these residues acting as a steric barrier narrowing the entrance to the active site cavity (Malik et al., 2012) is the decrease in amino donor specificities from (*S*)-methylbenzylamine to (*S*)-ethylbenzylamine which was also observed in Vf-ωTA (Nobili et al., 2015) and *Ochrobactrum anthropi* Oa-ωTA (Han et al., 2015). Several previous studies have mutated the corresponding Trp61 in homologous ωTA sequences resulting in improved activities toward aromatic and aliphatic amines without compromising enantioselectivity (Cho et al., 2008; Cassimjee et al., 2012). On the other hand, the highly conserved amino acid residues which surround the large hydrophobic binding pocket were identified as Leu60, Trp61, Ala232, Ile263, and Arg415 which has dual recognition for hydrophobic substituents and a carboxylate group (Hirotsu et al., 2005). This was shown to be in general agreement with previous studies of ω-TAs with high affinity for acetophenone derivatives as well as aliphatic methyl ketones and aldehydes (Kaulmann et al., 2007; Cerioli et al., 2015).

### Biocatalytic Synthesis of Benzylamines Derivatives

A plethora of ω-transaminases have been reported to aminate acetophenone derivatives and bulkier ketones. There have been recent reports on the use cinnamaldehyde derivatives as amine acceptor molecules using ω-transaminases from Cv-ωTA, Vf-ωTA, and isolated transaminase genes from metagenomic samples furnishing modest yields of the allylic amines (Baud et al., 2017). To our surprise, only a few have been described on the acceptance of benzylic aldehydes (Cerioli et al., 2015; Guidi et al., 2018). In one of the reports, the benzaldehydes were generated by biocatalytic oxidation of the corresponding benzylic alcohols, followed by transamination to the corresponding benzylamines (Fuchs et al., 2012). Interestingly, within the panel of ω-transaminases investigated, significant differences in the amination of benzaldehyde and analogs could be observed, transamination biocatalysts with a broad substrate tolerance toward this class of compounds are rare. Moreover, an additional cofactor recycling system in the presence of a sacrificial cosubstrate was essential to drive the equilibrium to completion.

**Figure 9:**
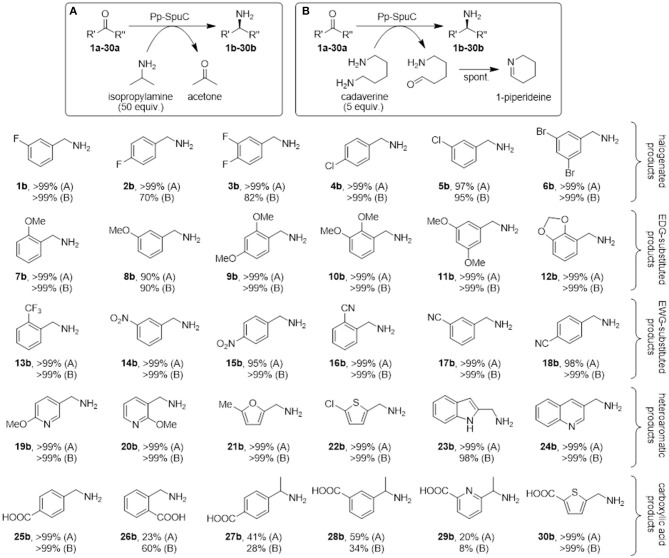
Biocatalytic synthesis of a range of substituted benzylamines mediated by Pp-SpuC. Reaction conditions: 5 mM substrate, 2 mg mL^−1^ Pp-SpuC, 1 mM PLP, 1% v/v DMSO, 250 mM isopropylamine **(A)** or 25 mM cadaverine **(B)**, in 50 mM Tris-HCl, pH 9.0, 30 °C, 18 h. Conversions were measured by LC-MS (Supplementary Information).

Since Pp-SpuC was already demonstrated to have high activity with a variety of prochiral ketones (Slabu et al., 2018), we envisaged to investigate the amination of a range of benzaldehyde analogs bearing halogen substituents (**1a-6a**), electron-donating groups (**7a-12a**), electron-withdrawing groups (**13a-18a**) and heteroatoms in the ring (**19a-24a**). Considering the importance of non-natural amino acids, we also investigated aromatic aldehydes and ketones that lead to pharmaceutically relevant examples of this class of compounds (**25a-30a**). All compounds were screened using either a large excess of isopropylamine as a donor (Figure 9A) or only a moderate excess of cadaverine, a biogenic amine donor that favors reaction equilibria by cyclising spontaneously to 1-piperideine after transamination (Figure 9B).

Mono- and dihalogenated benzaldehydes **1a**-**6a** afforded excellent conversions to the halogenated benzylamines **1b-6b** using isopropylamine as the amine donor within 18 h (in the preliminary screening, substrate **1a** was converted quantitatively in < 30 min with both amine donors). Interestingly, a decrease in conversions was observed using *para-*fluorinated substrates **2a-3a** with cadaverine as the amine donor. We previously observed a similar low conversion with a *para*-substituted fluoroacetophenone derivative (Galman et al., 2017) which is presumably ascribed to the interaction between the piperideine monomer from the transamination of cadaverine and the activated *para*-fluoro substrates. Encouraged by these results, other benzylic derivatives were tested to assess the stereoelectronic effects of electron-donating (methoxy, methylenedioxy), or electron-withdrawing (nitro, cyano, trifluoromethyl) substituents. All functional groups were well-tolerated, giving almost quantitative conversion with both amine donors (except for *m*-anisaldehyde **8a** that afforded amine **8b** in 90% conversion). No overall detrimental effects were observed due to the higher or lower electrophilicity of the carbonyl group due to the substituents, or due to the steric hindrance due to multiple substitutions at *ortho*-, *meta*-, and *para*-positions. Similarly, heteroaromatic aldehydes proved to be accepted very well by Pp-SpuC, affording complete conversions with pyridines (**19a-20a**), furan (**21a**), thiophene (**22a**), indoline (**23a**), and quinoline (**24a**) moieties.

In addition, we attempted to carry out the biocatalytic synthesis of benzylamines carrying the carboxylic acid functionality from the transamination of formylbenzoic acid derivatives (**25a**-**26a**). The *para*-substituted compound was readily accepted to produce the corresponding amine **25b** with excellent conversion, while the *ortho*-derivative gave considerably lower conversions to **26b** (23 and 60%, respectively), indicating the presence of a carboxylic acid substituent at the *ortho*-position can have a detrimental effect on the reactivity. This facile approach to generate **25b**, which is used as an antifibrinolytic agent, provides a sustainable alternative to other reductive amination protocols requiring toxic metal catalysts and harsh reaction conditions (Yraola et al., 2006). Prochiral acetylbenzoic acids **27a** and **28a** are considerably more challenging due to the presence of the methylketone moiety. Indeed only moderate conversions to **27b** (41%) and **28b** (59%) could be achieved. This can be rationalized by looking at the active site shape in the crystal structure of Pp-SpuC, which provides an insight into the substrate scope of the enzyme: the hydrophobic environment of the large pocket can accommodate large aromatic groups, presumably by stabilization from π-π stacking interactions, while the smaller pocket imposes a steric constraint on the other substituent of the carbonyl group and might hinder access to larger substituents. This explains why almost any aromatic aldehyde derivative is accepted easily, while the range of methyl (and higher) ketones is very limited, limiting the biocatalytic synthesis of chiral amines and is configured to accept aromatic aldehydes readily. Lastly, we screened heteroaryl oxoacid derivatives **29a** and **30a**. Unsurprisingly, **29a** afforded lower conversions (20 and 8%, respectively), presumably for the same reasons discussed above, while **30a** was converted completely, indicating that the presence of a carboxylic acid substituent does not have a detrimental effect on the reactivity of these heteroaromatic substrates.

## Conclusion

In summary, we have performed the biochemical characterization of putrescine transaminase Pp-SpuC which reveals a high relative activity at 60°C and excellent stability in 50 mM Tris-HCl at pH 8.0. The enzyme structure was obtained, showing a high degree of similarity to the apo form of Cv-ωTA (PDB id: 4A6U) which can accommodate large aromatic substrates for catalysis. We recognized the important dual substrate recognition of Pp-SpuC, accepting cadaverine or isopropylamine as the equilibrium shifting amine donor, and having excellent activity against a broad range of aromatic aldehyde derivatives bearing various electron-donating and electron-withdrawing substituents, as well as different heteroatoms in the ring, with excellent conversions. This method also eliminates the need for an expensive cofactor recycling system making this approach to important amine scaffolds greener and more economically feasible.

## Materials and Methods

### General Methods

All commercially available reagents, analytical standards, and solvents were purchased from Sigma-Aldrich, Alfa Aesar, or Fluorochem and used without further purification. *Escherichia coli* DH5α and BL21 (DE3) cells were purchased from New England Biolabs (Ipswich, MA, USA). Expression vector pET-28b was purchased from Novagen (Darmstadt, Germany) and was used for gene expression. *P. putida* NBRC 14161 (Asc no. 9494) was purchased from the NCIMB culture collection (Aberdeen, UK). The putrescine transaminase *spuC* from *P. putida* was cloned in a pET-28b vector containing an N-terminal His_6_-tag and was recombinantly overexpressed in *E. coli* BL21 (DE3) cells according to a previously published procedure (Galman et al., 2017) with slight modifications.

### Protein Expression and Purification

Plasmid pET28b-SpuC was transformed into *E. coli* BL21 (DE3). A fresh colony was used to inoculate LB medium (4 mL) containing kanamycin (50 μg mL^−1^). This freshly prepared overnight culture was grown at 220 rpm at 37°C, and was used to inoculate 500 mL of LB medium supplemented with kanamycin (50 μg mL^−1^) in a 2 L Erlenmeyer flask at 220 rpm at 37°C. The recombinant protein expression was induced by adding isopropyl-β-D-1-thiogalactopyranoside (IPTG) (0.2 mM, final) when OD_600_ reached 0.6–0.8. The cell cultures were then incubated at 18°C for 16 h. The cells were harvested by centrifugation at 4°C (3,250 g, 20 min) and were resuspended (1g in 10 mL) in lysis buffer (50 mM Tris-HCl, 5 mM imidazole, pH 7.0) and lysed in an iced bath by ultra-sonication by Soniprep 150 (20 s on, 20 s off, for 20 cycles, at 30% amplitude). After centrifugation (4°C, 16,000 g, 20 min) the clarified lysate was used for protein purification via a Ni-NTA agarose column. The bound enzyme was washed with 20 mL wash buffer (50 mM Tris-HCl, 30 mM imidazole pH 7.0), and eluted with 50 mM Tris-HCl, 250 mM imidazole at pH 7.0. The collected fractions were concentrated in Vivaspin^TM^ filter spin membrane columns (10,000 MWCO). The purified enzyme was buffer exchanged using PD-10 desalting columns using 50 mM Tris-HCl containing 1 mM PLP at pH 9.0. The purity was analyzed by SDS/PAGE and the protein was more than 95% pure and the protein stock was determined by the Bradford assay using bovine serum albumin as standard.

### Biotransformations and LC-MS Analysis

Unless otherwise specified, all biotransformation were performed in 2 mL Eppendorf tubes, at 30°C in biotransformation buffer: Tris-HCl (50 mM) containing PLP (1 mM), pH 9.0. To the addition of amine donor, cadaverine (25 mM), or isopropylamine (250 mM) in biotransformation buffer adjusted to pH 9.0 and carbonyl substrate (5 mM, from a 500 mM stock solution in DMSO), was added purified Pp-SpuC (2 mg mL^−1^, purified as described above) in a final volume of 0.5 mL. After 18 h, the reaction mixture was quenched by adding aqueous NaOH solution (100 μL, 2 M).

The residual activities of the enzyme after incubating at 50 and 60°C at the allotted time was determined by the generation of acetophenone from (*S*)-methylbenzylamine with pyruvate as the amine acceptor substrate molecule (Schätzle et al., 2009). The absorbance was measured at 300 nm using a UV transparent 96-well plate (Tecan infinite M200 spectrophotometer, Austria). One unit of enzyme activity (U) was defined as the amount of enzyme required to produce 1 μmole of acetophenone produced per min. The deactivation rate constant k_d_ was determined from the slope and the half-lives were calculated as t_1/2_ = ln2/k_d_.

The effect of pH on enzyme stability was carried at various pH buffers from pH 4.0–10.5. The enzyme (2 mg mL^−1^) was incubated for 24 h at 4°C in different buffers containing 1 mM PLP: sodium acetate (pH 4.0–6.0), sodium phosphate (pH 6.0–8.0), Tris-HCl (pH 7.0–9.0), and sodium carbonate/bicarbonate (pH 9.2–10.0). The residual activity was assayed from the biotransformations as described above.

The effect of solvent stability was performed using a variety of different co-solvents (DMSO, MeOH, MeCN, DMF, and THF) at different solvent concentrations % (v/v) using 5 mM of **1a**, and 2 mg mL^−1^ of purified Pp-SpuC, 1 mM PLP, and 250 mM isopropylamine at pH 9.0.

Conversions were calculated by HPLC analysis on an Agilent 1,100 Series system equipped with a quaternary pump, a HiChrom ACE 5 C18-AR column (15cm × 4.6 mm), a diode UV array detector and an Agilent LC/MSD SL LCMS System. Separation conditions: 1 mL min^−1^ flowrate, mobile phases: H_2_O + 0.1% v/v TFA, MeOH + 0.1% v/v TFA. Gradient: 95:5 for 4 min, to 0:100 over 25 min. Temperature: 30°C. Detection wavelength: 210 nm. Injection volume: 10 μL. Compounds were ionized using API-electrospray technique and detected in positive mode on the LCMS System. Drying gas temperature 250°C at 12 L min^−1^, and nebulizer pressure at 25 psig. All products, unless otherwise specified, were identified by their [M+H]^+^ signal and confirmed via chemical standards.

### Crystallization and Structure Determination

C-terminal His-tagged Pp-SpuC purified by affinity chromatography as described above was exchanged to 20 mM HEPES buffer pH 7.5 and submitted to an additional step of ion-exchange purification on a ResourceQ column (6 mL, GE healthcare), to further purify the protein for crystallogenesis. Pp-SpuC was loaded on the column in 20 mM HEPES pH 7.5 buffer and elution was performed with a linear gradient (25 column volumes) of NaCl in buffer. All the fractions were analyzed by SDS-PAGE. Fractions containing Pp-SpuC were pooled and concentrated in a Vivaspin™ filter spin membrane columns (10,000 MWCO).

Apo and PLP (equimolar) mixed protein was screened against commercially available crystallogenesis screens using a mosquito nanodispenser robot (LLP Biotech). Overnight crystals appeared in a number of conditions at 20°C. The best diffracting crystals were obtained in 0.2 M MgCl_2_, 0.1 M Bis-Tris pH 6.5, 25% PEG3350. SpuC crystals cryoprotected with 10–20% PEG200 in mother liquor before freezing. Data was collected from single crystal at IO4 beamline at the Diamond Light Source Syncrotron (UK). Data was indexed and integrated with XDS (Kabsch, 2010) and scaled with AIMLESS (Evans and Murshudov, 2013). Data processing and refinement statistics are summarized in Table 1.

**Table 1:** Data collection and refinement statistics.

Wavelength	0.9795
Resolution range	59.45–2.05 (2.123–2.05)
Space group	P 2_1_ 2_1_ 2_1_
Unit cell	62.55 96.12 151.32 90 90 90
Unique reflections	57,573 (5,674)
Multiplicity	3.6 (3.7)
Completeness (%)	99.02 (98.72)
Mean I/σ(I)	9.5 (1.7)
Wilson B-factor	31.73
R-meas	0.106
CC1/2	1.0
Reflections used in refinement	57,494 (5,644)
Reflections used for R-free	2,880 (293)
R-work	0.2042 (0.2691)
R-free	0.2473 (0.3284)
Number of non-hydrogen atoms	6,425
-Macromolecules	5,907
-Solvent	518
Protein residues	762
RMS(bonds)	0.009
RMS(angles)	1.01
Ramachandran favored (%)	94.53
Ramachandran allowed (%)	4.93
Ramachandran outliers (%)	0.53
Rotamer outliers (%)	0.49
Clashscore	9.94
Average B-factor	39.72
-Macromolecules	39.37
-Solvent	43.69

*Statistics for the highest-resolution shell are shown in parentheses*.

The structure of Pp- SpuC was solved using molecular replacement method by Phaser (Eliot and Kirsch, 2004). Crystal structure of Pse-ωTA (PDB id: 5TI8, sequence identity 77%) was used as a phasing model. Iterative cycles of manual building in COOT (Emsley et al., 2010) and refinement in Phenix.refine (Murshudov et al., 1997; Eliot and Kirsch, 2004) were carried out to complete the model. Validation of structure was done with Molprobity (Chen et al., 2010) and PDB-redo (Joosten et al., 2014) were integrated into the iterative building and refinement procedure. The final refined structure of Pp-SpuC has been deposited in the PDB database (PDB id: 6HX9).

## Author Contributions

JG, FP, and NT conceived the project. JG performed molecular biology procedures associated with gene cloning and construct preparations. IS worked on enzyme preparations and biochemical characterisations. FP performed the LC-MS analytics. DG, and DL undertook all structural analyses.

### Conflict of Interest Statement

The authors declare that the research was conducted in the absence of any commercial or financial relationships that could be construed as a potential conflict of interest.
