# Supplementary material for: Characterization of a Putrescine Transaminase From Pseudomonas putida and its Application to the Synthesis of Benzylamine Derivatives
Source: Front Bioeng Biotechnol. 2018 Dec 21;6:205. doi: 10.3389/fbioe.2018.00205 (PMC6308316; doi:10.3389/fbioe.2018.00205)
Supplement: Supplementary file 1 [file Data_Sheet_1.pdf]

Characterisation of a putrescine transaminase from *Pseudomonas putida* and its application to the synthesis of benzylamine derivatives

James L. Galman,[a] Deepankar Gahloth,[a] Fabio Parmeggiani,[a] Iustina Slabu,[a] David Leys,[a] Nicholas J. Turner*[,a]

[a] School of Chemistry, The University of Manchester, Manchester Institute of Biotechnology,
131 Princess Street, Manchester, M1 7DN, United Kingdom

SUPPLEMENTARY INFORMATION

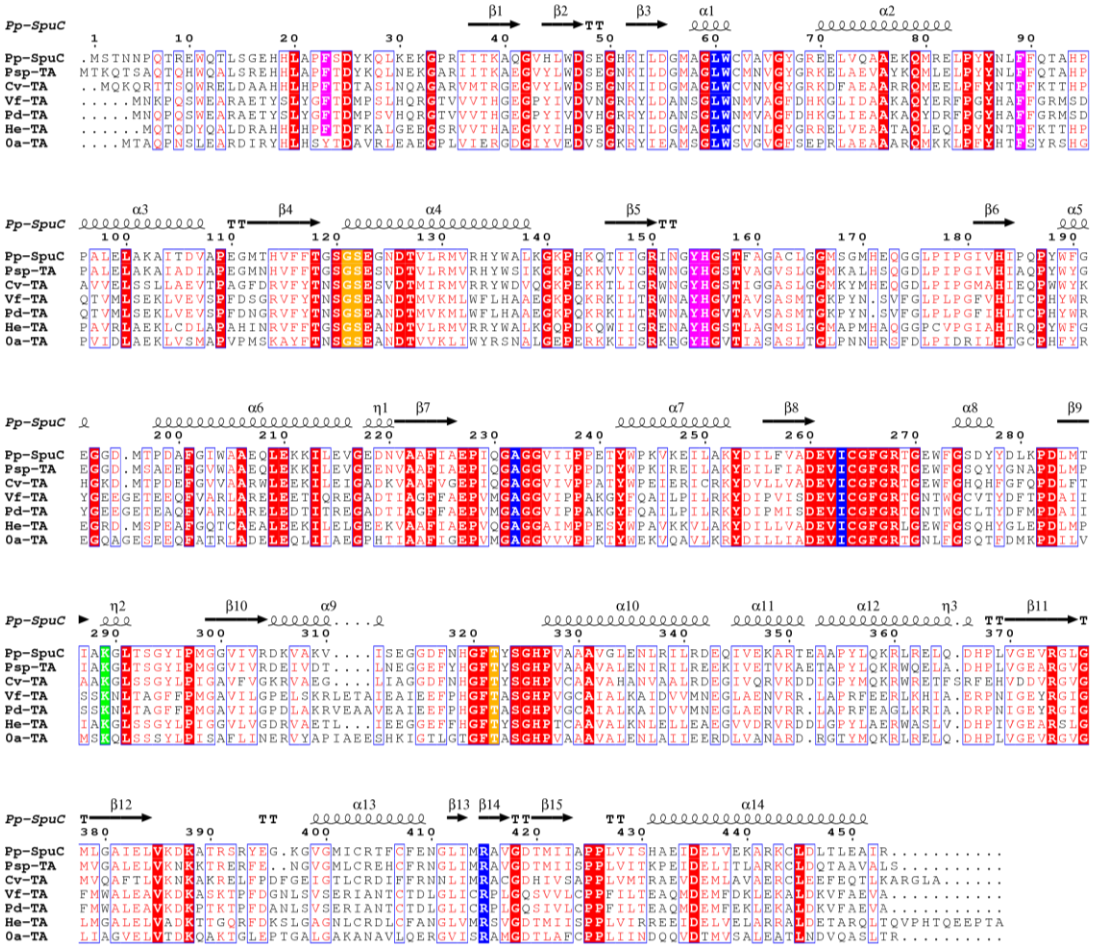

Figure S1: Protein sequence alignment (with secondary structure) of homologous ω-TAs: *P. putida* Pp-SpuC, *Pseudomonas sp*. Psp-ωTA, *C. violaceum* Cv-ωTA, *V. fluvialis* Vf-ωTA, *P. denitrificans* Pd-ωTA, *H. elongata* He-ωTA and *O. anthropic* Oa-ωTA. The amino acid numbering on top of the sequences is for Pp-SpuC. The catalytic lysine is highlighted in green and the residues which form hydrogen bonds with the phosphate binding cup are highlighted in orange. The active site residues in the small substrate binding pocket are coloured pink and the large substrate binding pocket are highlighted in blue. The white letters on the red background represent fully conserved residues. The structure based sequence alignment was generated using ESPript[1]

(1) Gouet, P. *Nucleic Acids Res.* **2003**, *31* (13), 3320–3323.

LC-MS traces of Pp-SpuC biotransfomations of 1-30a with isopropylamine (A) or cadaverine (B) as the amine donor

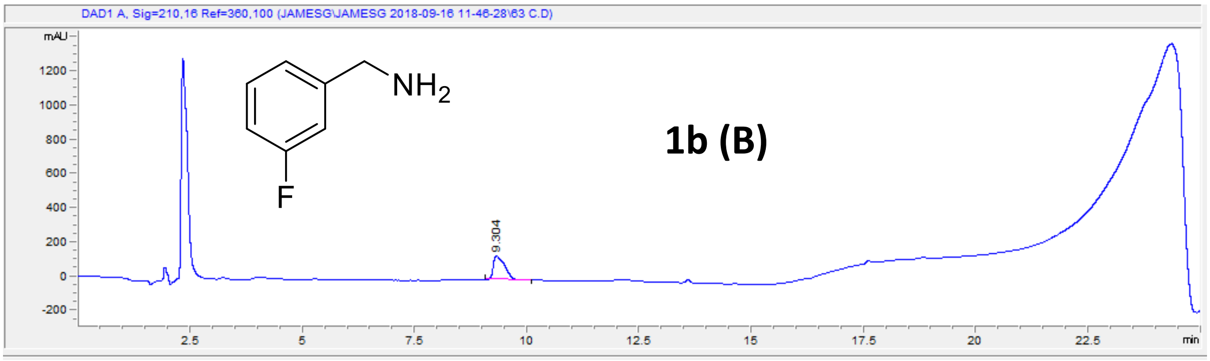

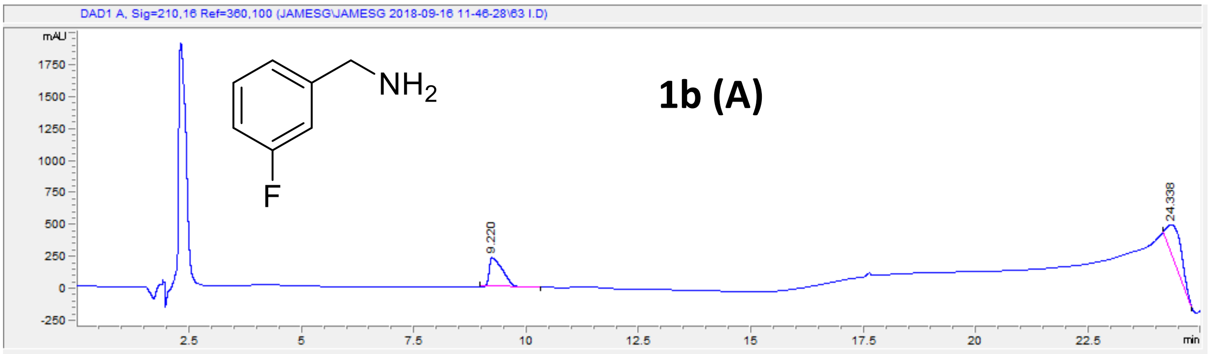

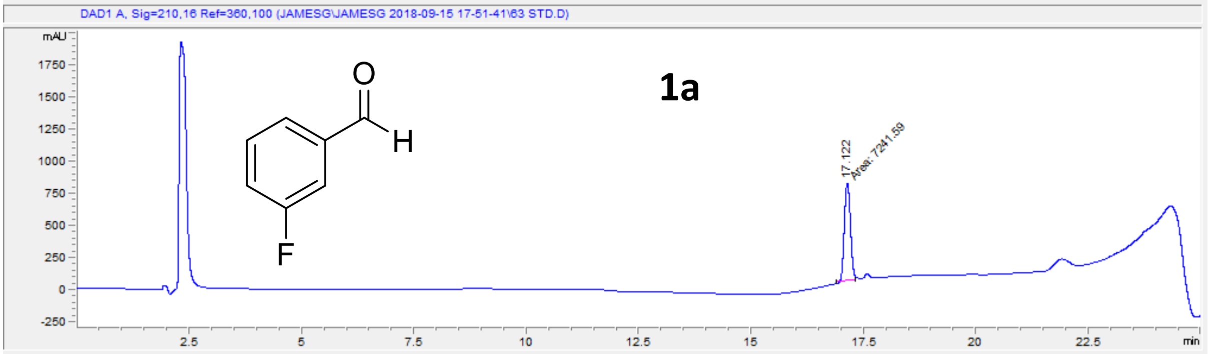

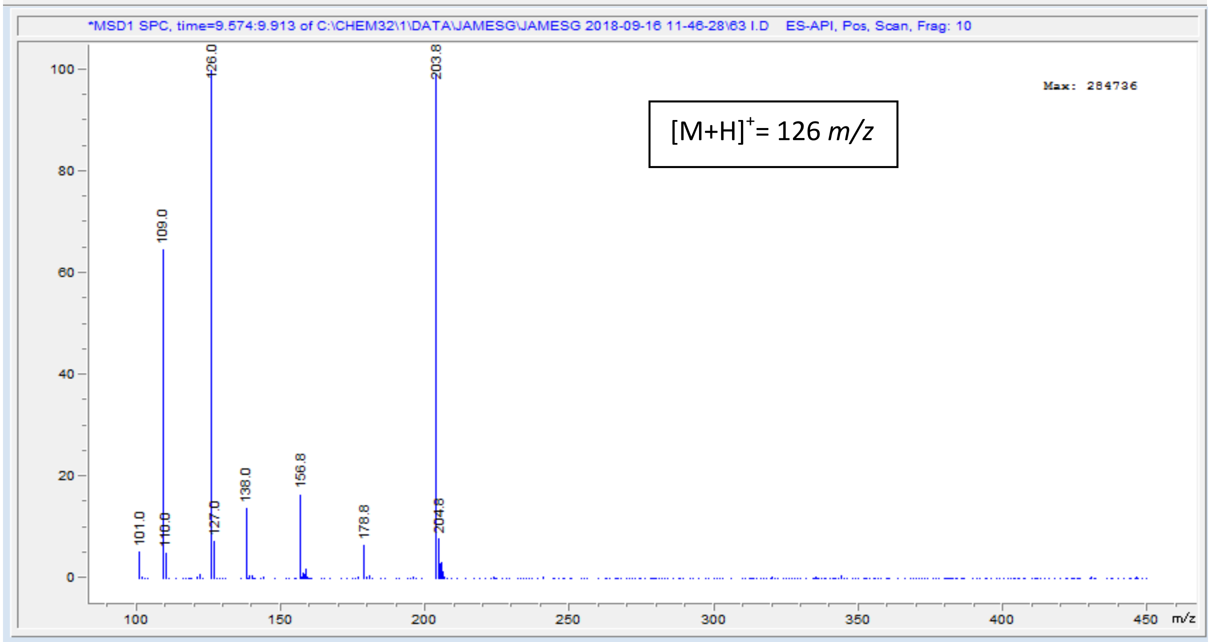

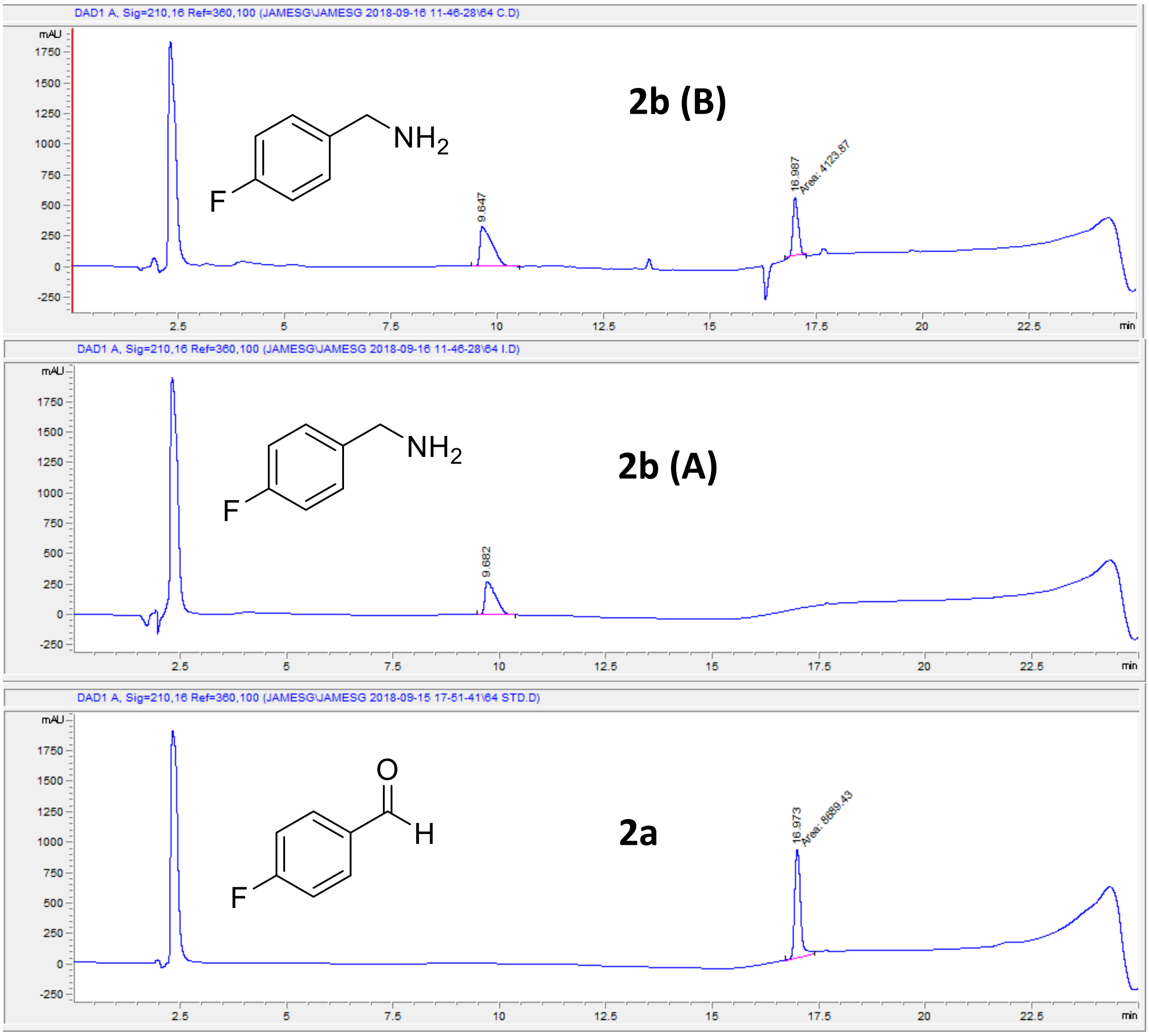
DAD1 A, Sig=210,16 Ref=360,100 (JAMESG\JAMESG 2018-09-16 11-46-28\64 C.D)
2b (B)
NH_2
F
9.647
16.987
Area: 4123.87
DAD1 A, Sig=210,16 Ref=360,100 (JAMESG\JAMESG 2018-09-16 11-46-28\64 I.D)
2b (A)
NH_2
F
9.682
DAD1 A, Sig=210,16 Ref=360,100 (JAMESG\JAMESG 2018-09-15 17-51-41\64 STD.D)
2a
O
H
F
16.973
Area: 5689.43
mAU
min

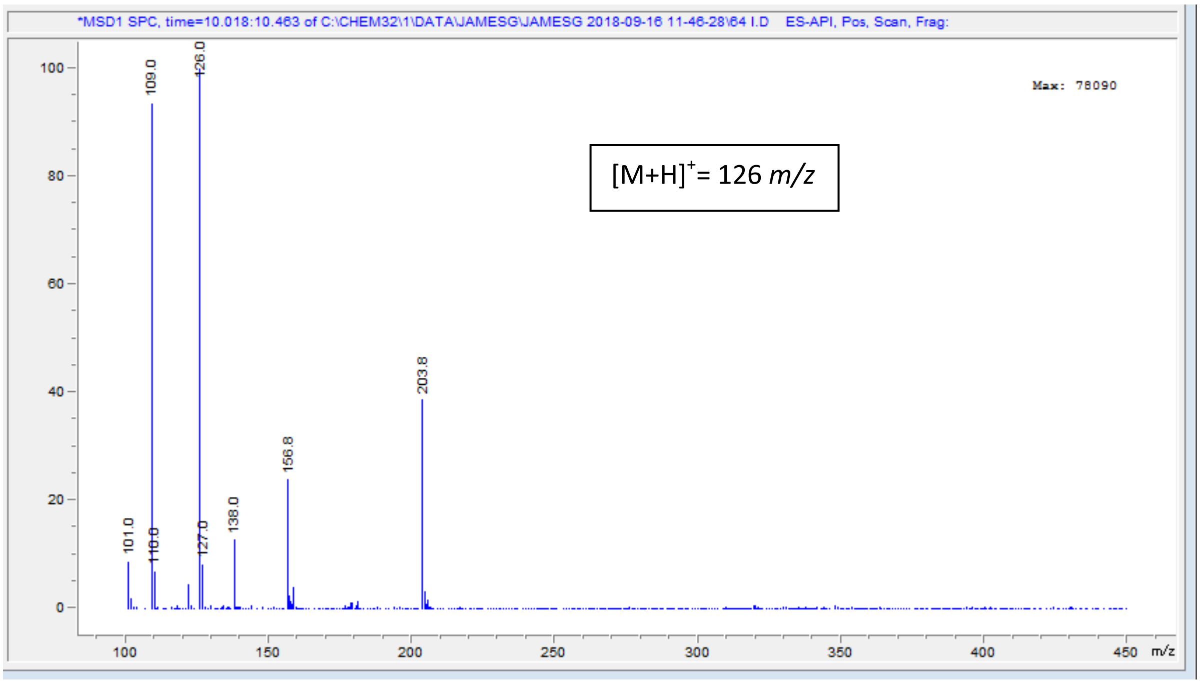
*MSD1 SPC, time=10.018:10.463 of C:\CHEM32\1\DATA\JAMESG\JAMESG 2018-09-16 11-46-28\64 I.D ES-API, Pos, Scan, Frag:
Max: 78090
$[M+H]^+$= 126 m/z
101.0
109.0
110.0
126.0
127.0
138.0
156.8
203.8
m/z

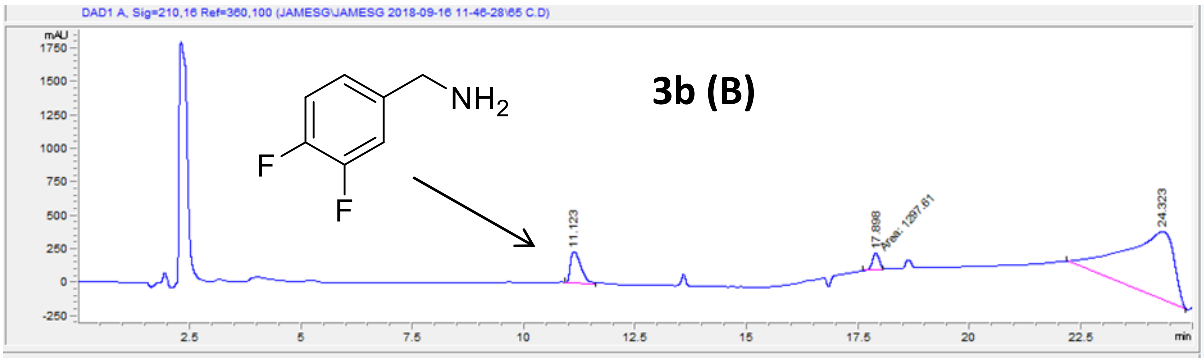
DAD1 A, Sig=210,16 Ref=360,100 (JAMESG\JAMESG 2018-09-16 11-46-28\65 C.D)
3b (B)
NH2
F
F
11.123
17.898
Area: 1297.61
24.323
mAU
min

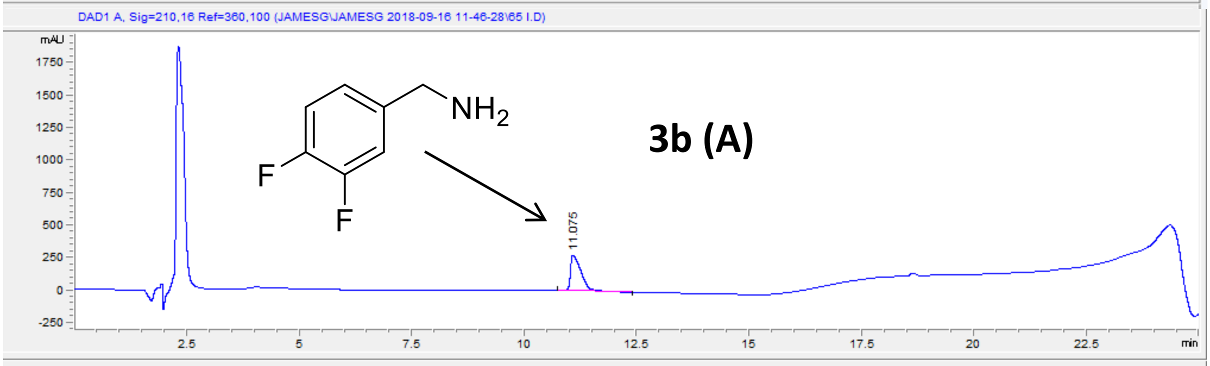
DAD1 A, Sig=210,16 Ref=360,100 (JAMESG\JAMESG 2018-09-16 11-46-28\65 I.D)
3b (A)
NH2
F
F
11.075
mAU
min

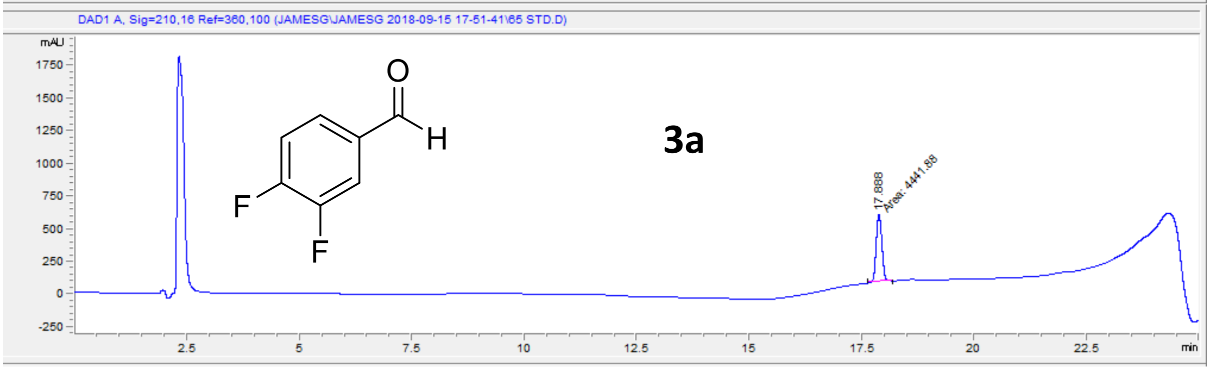
DAD1 A, Sig=210,16 Ref=360,100 (JAMESG\JAMESG 2018-09-15 17-51-41\65 STD.D)
3a
O
H
F
F
17.888
Area: 4441.88
mAU
min

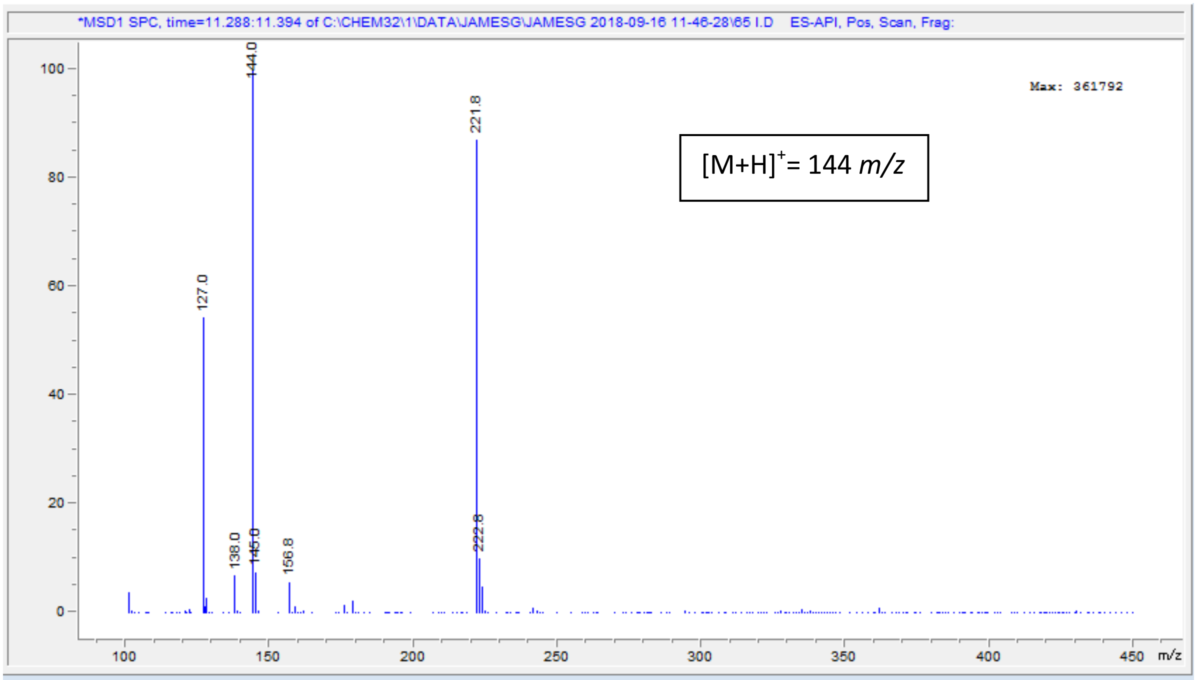
*MSD1 SPC, time=11.288:11.394 of C:\CHEM32\1\DATA\JAMESG\JAMESG 2018-09-16 11-46-28\65 I.D ES-API, Pos, Scan, Frag:
Max: 361792
[M+H]+= 144 m/z
144.0
221.8
127.0
138.0
145.0
156.8
222.8
m/z

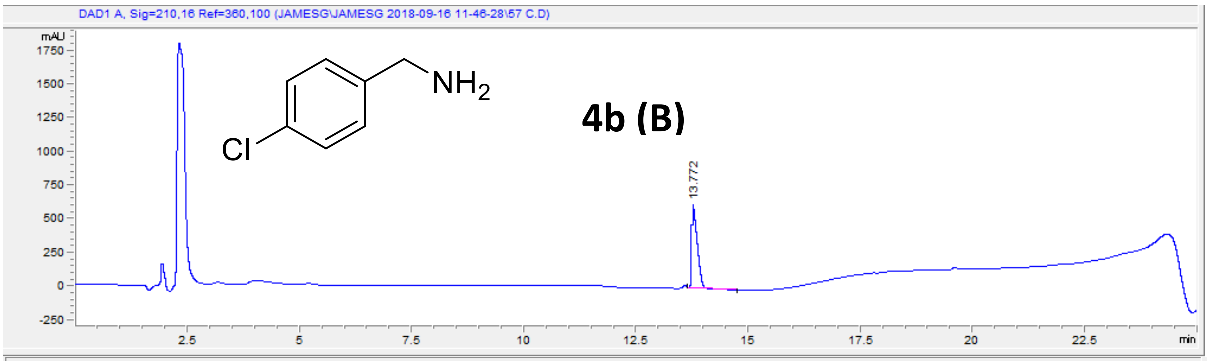
DAD1 A, Sig=210,16 Ref=360,100 (JAMESG\JAMESG 2018-09-16 11-46-28\57 C.D)
mAU
Cl
NH2
4b (B)
13.772
min

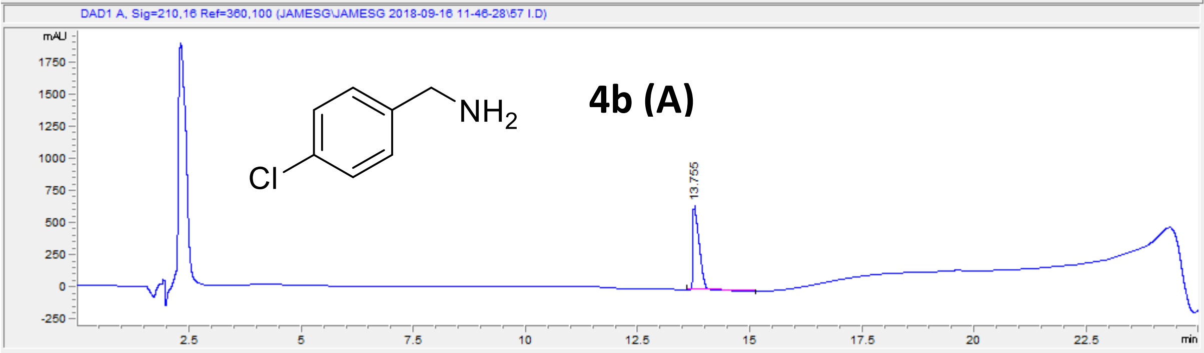
DAD1 A, Sig=210,16 Ref=360,100 (JAMESG\JAMESG 2018-09-16 11-46-28\57 I.D)
mAU
Cl
NH2
4b (A)
13.755
min

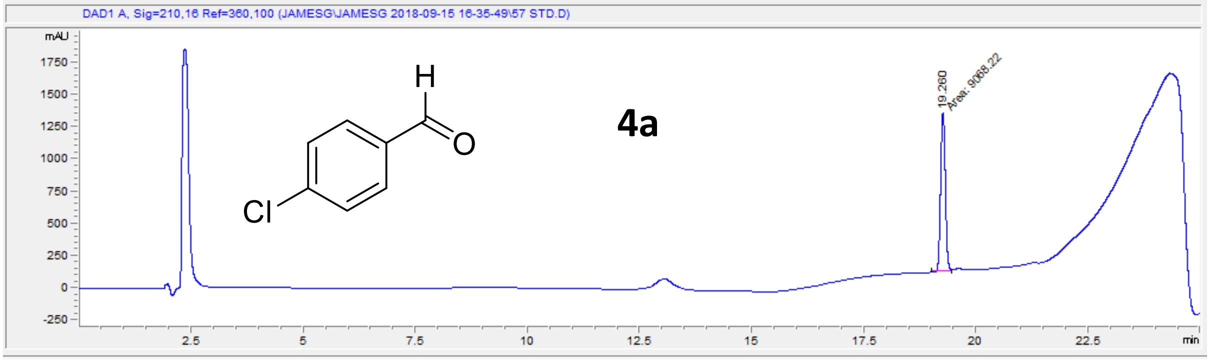
DAD1 A, Sig=210,16 Ref=360,100 (JAMESG\JAMESG 2018-09-15 16-35-49\57 STD.D)
mAU
H
O
Cl
4a
19.260
Area: 9068.22
min

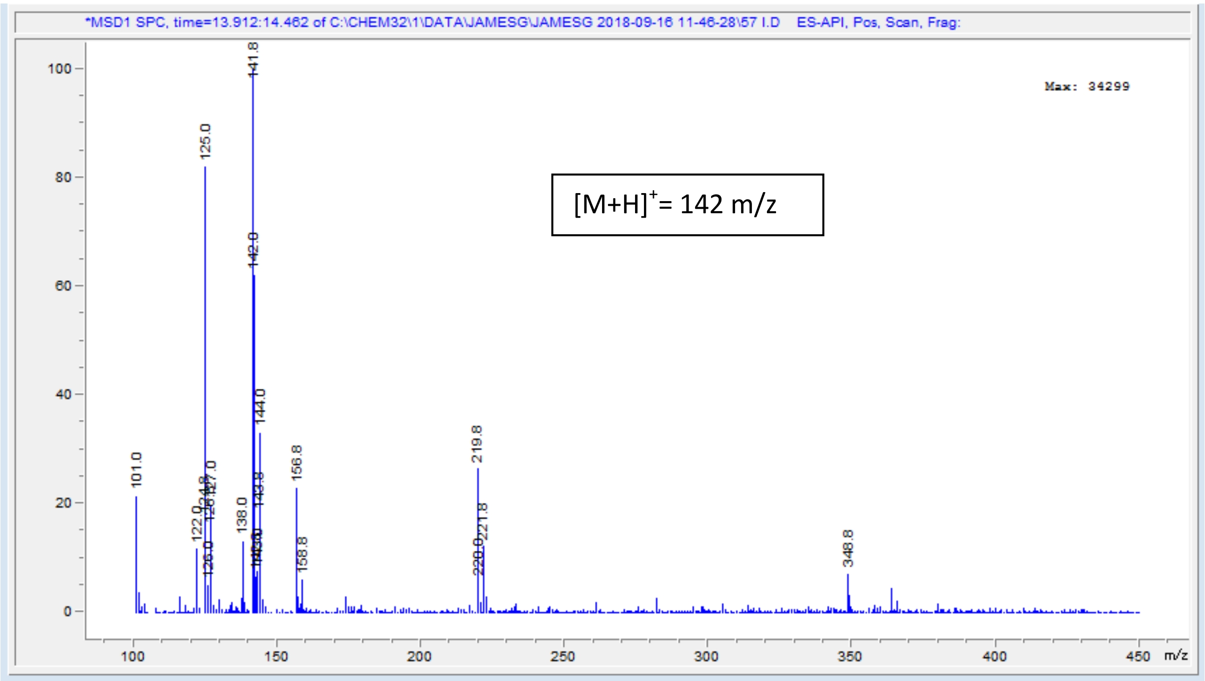
*MSD1 SPC, time=13.912:14.462 of C:\CHEM32\1\DATA\JAMESG\JAMESG 2018-09-16 11-46-28\57 I.D ES-API, Pos, Scan, Frag:
Max: 34299
[M+H]+= 142 m/z
141.8
125.0
142.0
144.0
101.0
156.8
219.8
348.8
138.0
m/z

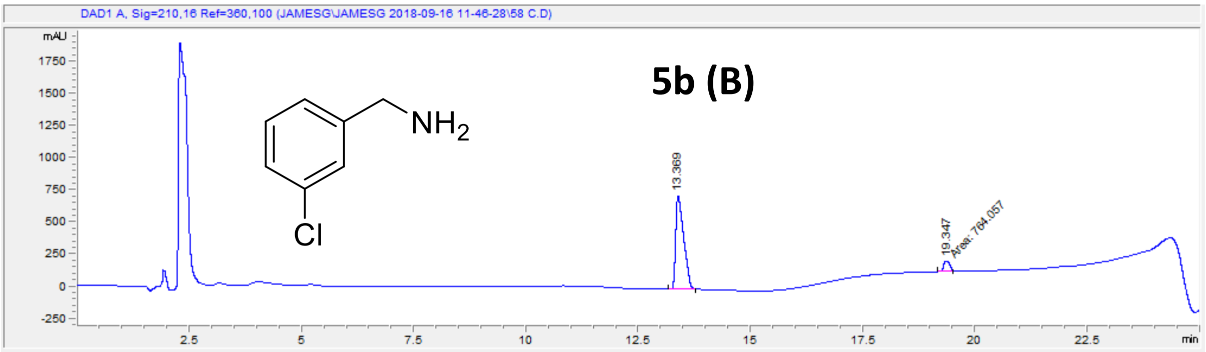
DAD1 A, Sig=210,16 Ref=360,100 (JAMESG\JAMESG 2018-09-16 11-46-28\58 C.D)
mAU
5b (B)
NH2
Cl
13.369
19.347
Area: 764.057
min

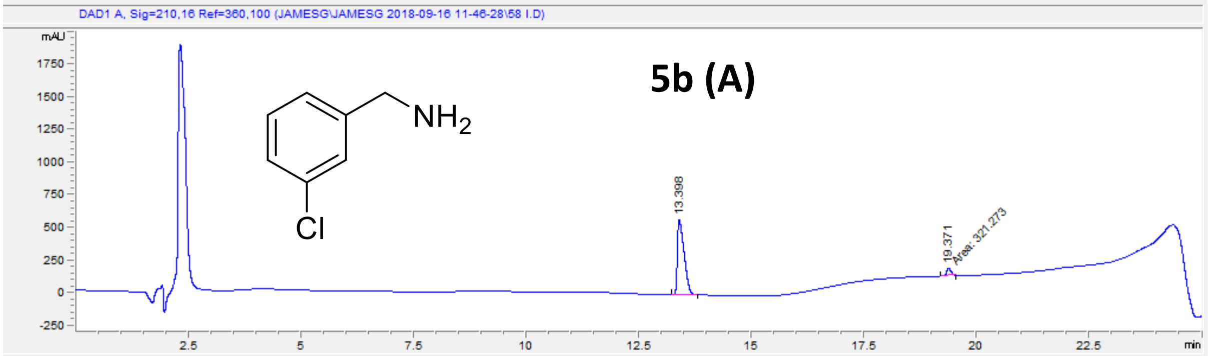
DAD1 A, Sig=210,16 Ref=360,100 (JAMESG\JAMESG 2018-09-16 11-46-28\58 I.D)
mAU
5b (A)
NH2
Cl
13.398
19.371
Area: 321.273
min

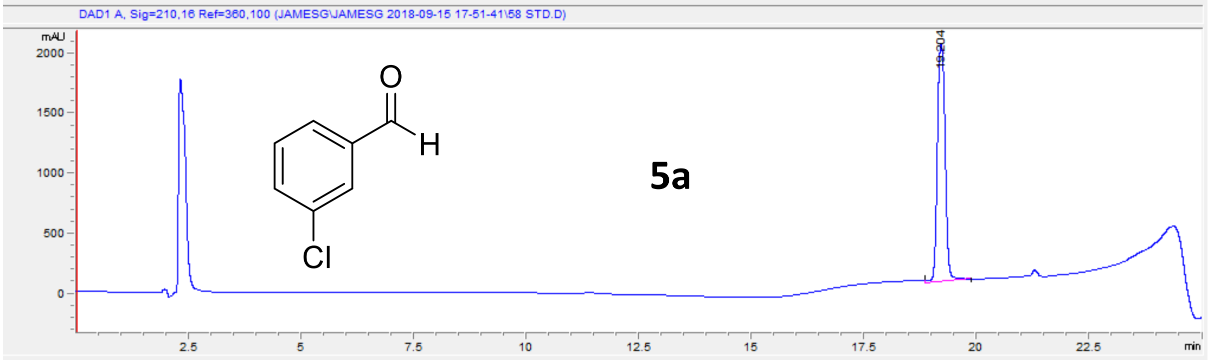
DAD1 A, Sig=210,16 Ref=360,100 (JAMESG\JAMESG 2018-09-15 17-51-41\58 STD.D)
mAU
O
H
Cl
5a
min

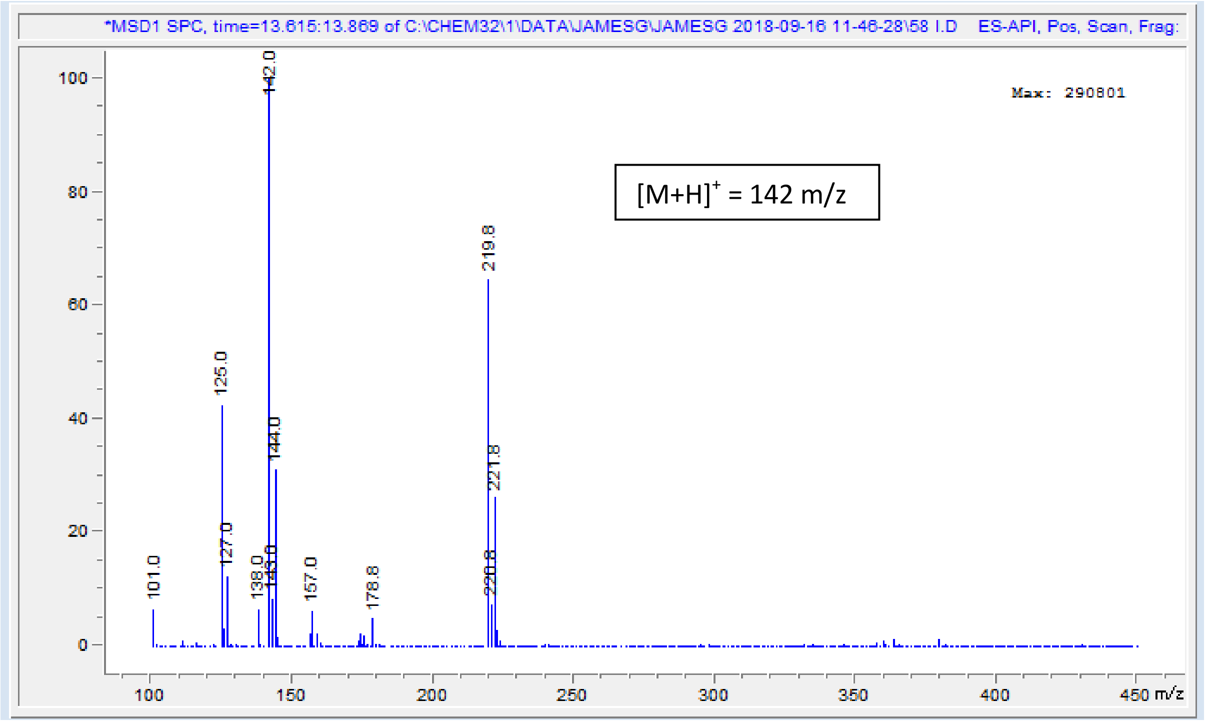
*MSD1 SPC, time=13.615:13.869 of C:\CHEM32\1\DATA\JAMESG\JAMESG 2018-09-16 11-46-28\58 I.D ES-API, Pos, Scan, Frag:
Max: 290801
[M+H]+ = 142 m/z
142.0
219.8
125.0
144.0
221.8
127.0
101.0
138.0
157.0
178.8
m/z

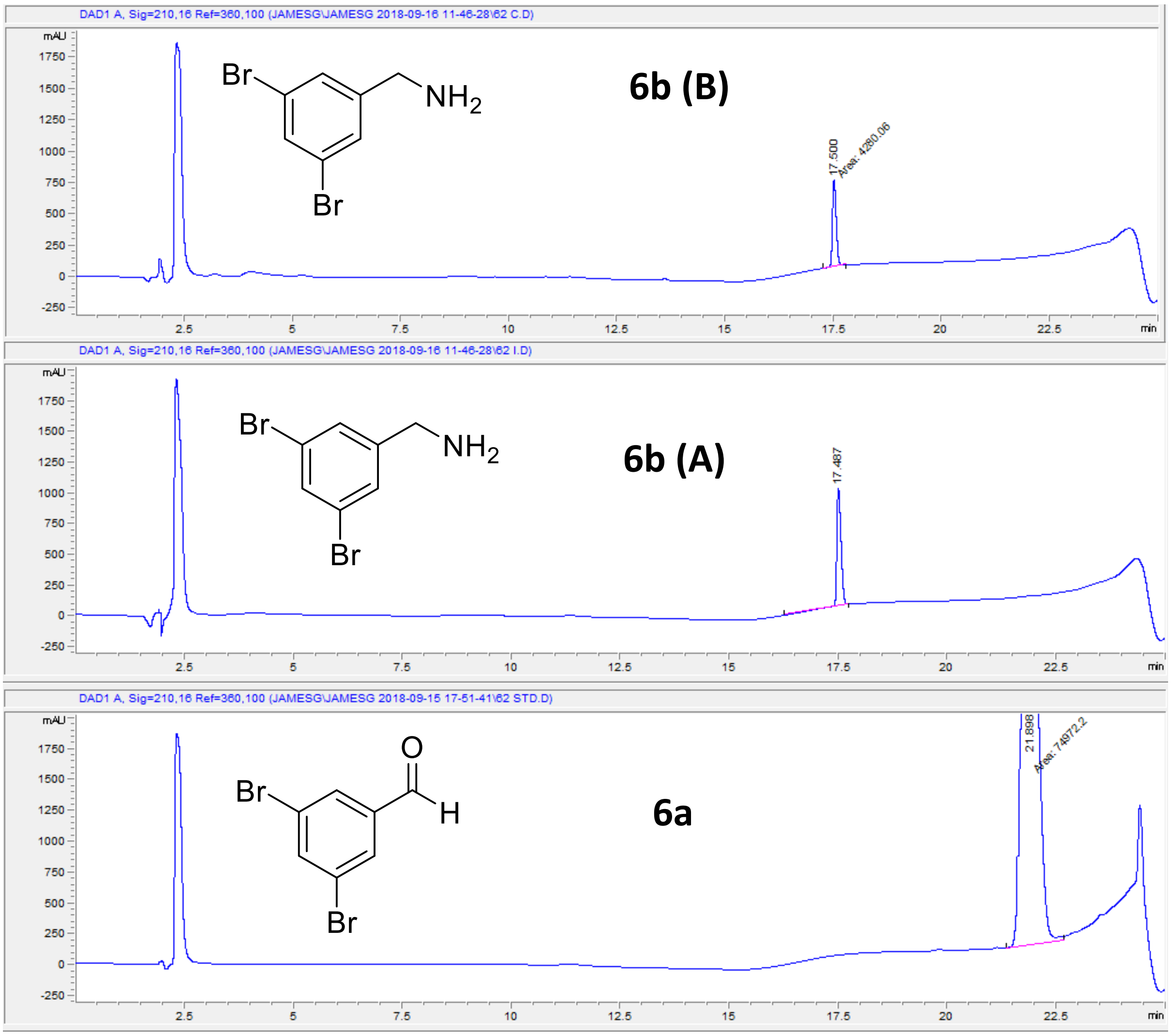
DAD1 A, Sig=210,16 Ref=360,100 (JAMESG\JAMESG 2018-09-16 11-46-28\62 C.D)
6b (B)
Br
NH2
Br
17.500
Area: 4280.06
DAD1 A, Sig=210,16 Ref=360,100 (JAMESG\JAMESG 2018-09-16 11-46-28\62 I.D)
6b (A)
Br
NH2
Br
17.487
DAD1 A, Sig=210,16 Ref=360,100 (JAMESG\JAMESG 2018-09-15 17-51-41\62 STD.D)
6a
O
H
Br
Br
21.898
Area: 74972.2
mAU
min

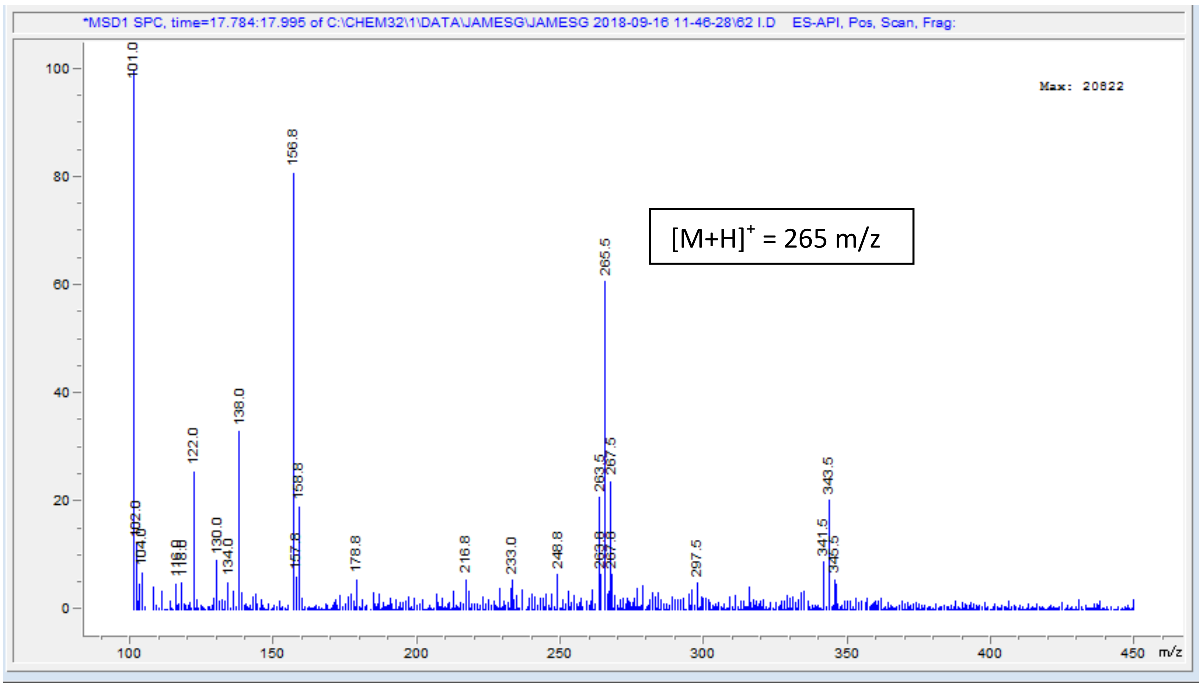
*MSD1 SPC, time=17.784:17.995 of C:\CHEM32\1\DATA\JAMESG\JAMESG 2018-09-16 11-46-28\62 I.D ES-API, Pos, Scan, Frag:
Max: 20822
[M+H]+ = 265 m/z
101.0
156.8
265.5
138.0
122.0
343.5
267.5
263.5
158.8
m/z

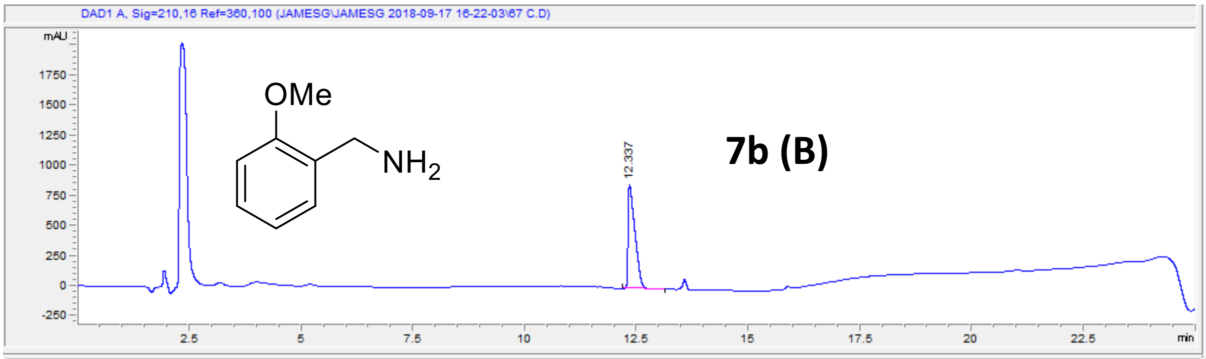
DAD1 A, Sig=210,16 Ref=360,100 (JAMESG\JAMESG 2018-09-17 16-22-03\67 C.D)
OMe
NH2
7b (B)
12.337
mAU
min

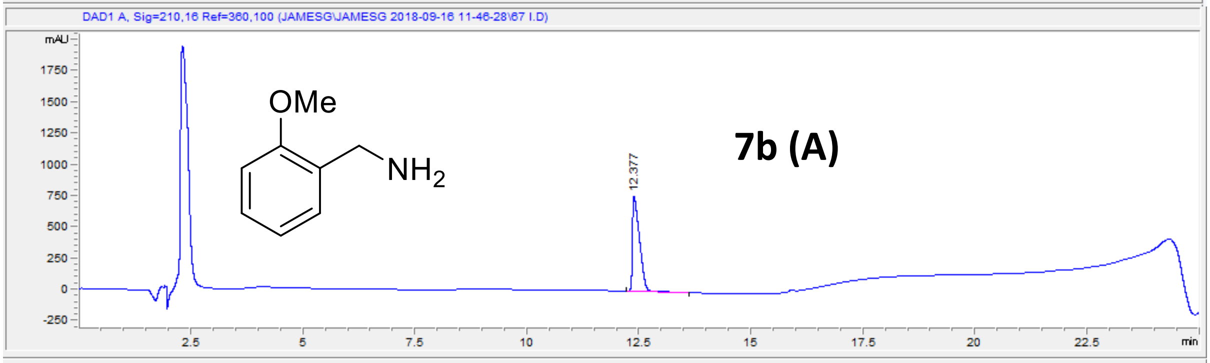
DAD1 A, Sig=210,16 Ref=360,100 (JAMESG\JAMESG 2018-09-16 11-46-28\67 I.D)
OMe
NH2
7b (A)
12.377
mAU
min

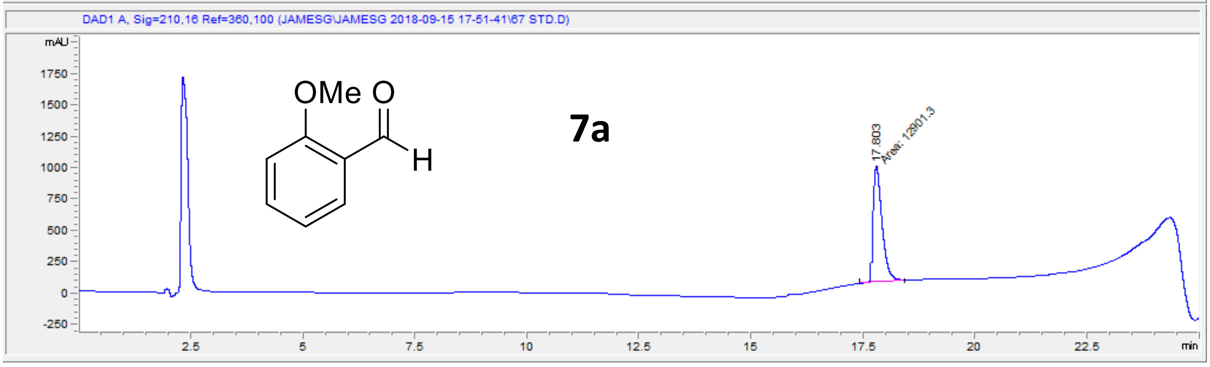
DAD1 A, Sig=210,16 Ref=360,100 (JAMESG\JAMESG 2018-09-15 17-51-41\67 STD.D)
OMe O
H
7a
17.803
Area: 12901.3
mAU
min

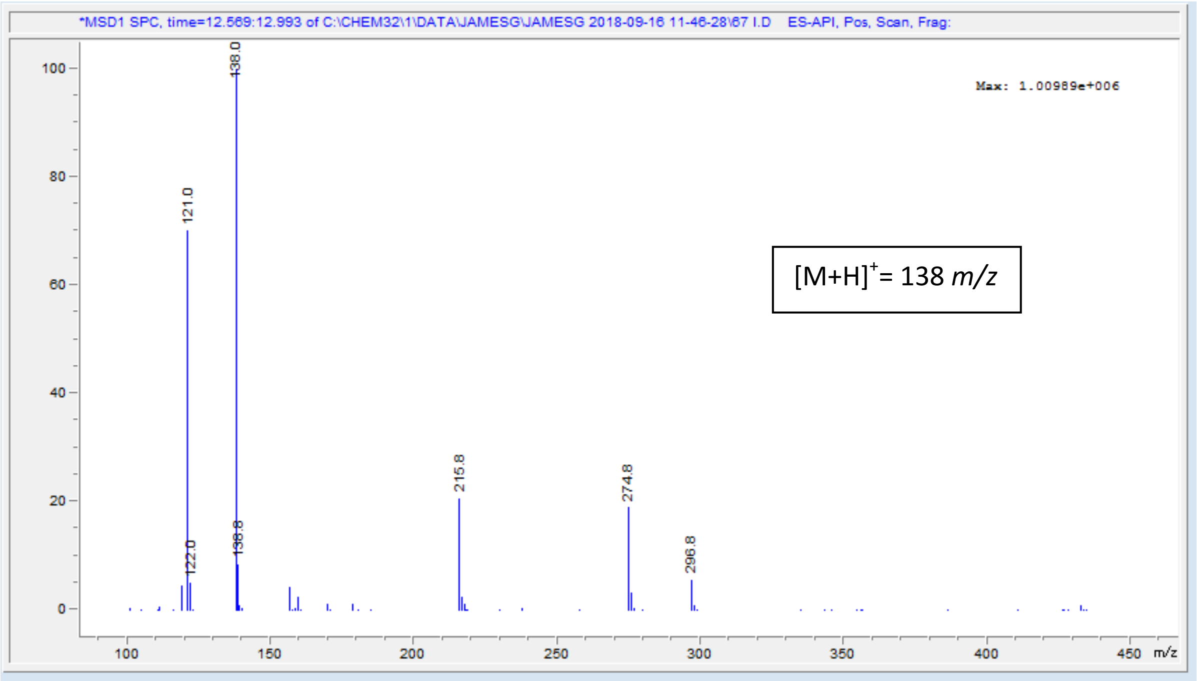
*MSD1 SPC, time=12.569:12.993 of C:\CHEM32\1\DATA\JAMESG\JAMESG 2018-09-16 11-46-28\67 I.D ES-API, Pos, Scan, Frag:
Max: 1.00989e+006
138.0
121.0
122.0
138.8
215.8
274.8
296.8
[M+H]+= 138 m/z
m/z

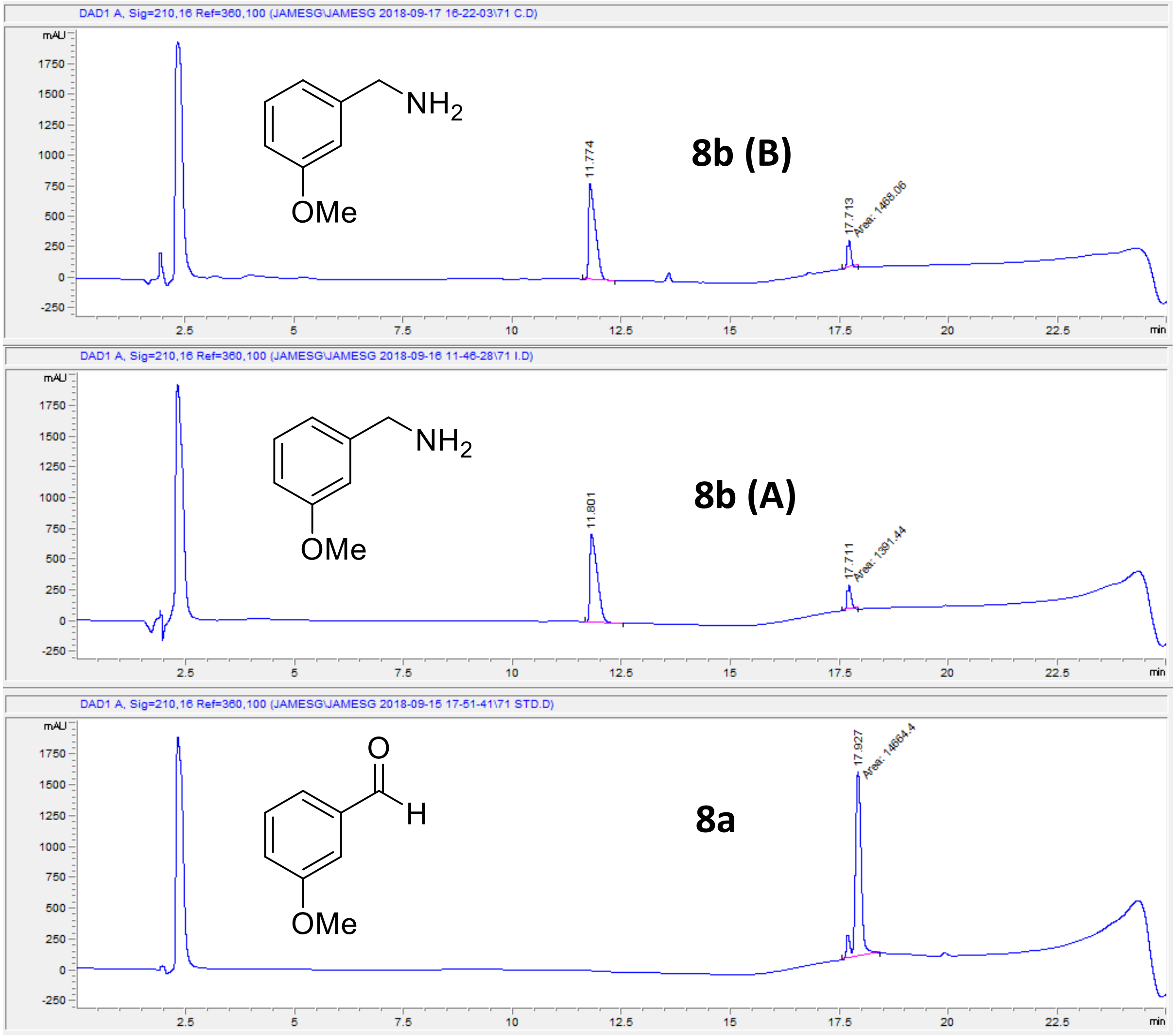

DAD1 A, Sig=210,16 Ref=360,100 (JAMESG\JAMESG 2018-09-17 16-22-03\71 C.D)
NH2
OMe
8b (B)
11.774
17.713
Area: 1468.06
DAD1 A, Sig=210,16 Ref=360,100 (JAMESG\JAMESG 2018-09-16 11-46-28\71 I.D)
NH2
OMe
8b (A)
11.801
17.711
Area: 1391.44
DAD1 A, Sig=210,16 Ref=360,100 (JAMESG\JAMESG 2018-09-15 17-51-41\71 STD.D)
O
H
OMe
8a
17.927
Area: 14664.4
mAU
min

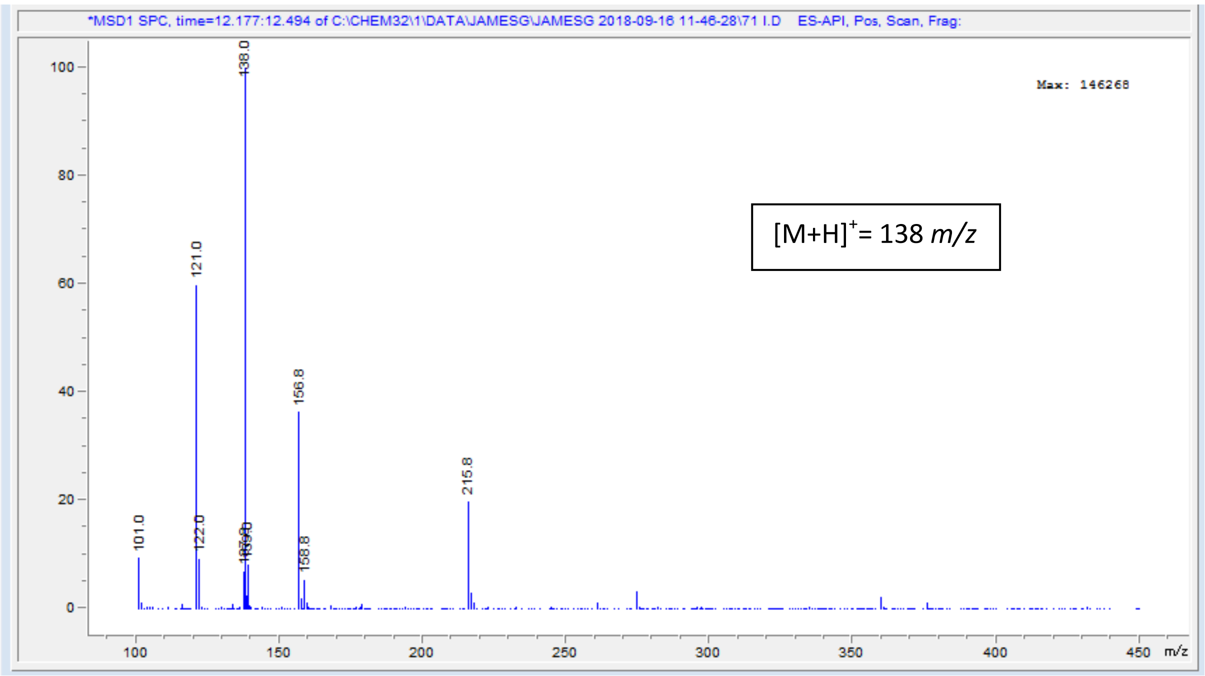

*MSD1 SPC, time=12.177:12.494 of C:\CHEM32\1\DATA\JAMESG\JAMESG 2018-09-16 11-46-28\71 I.D ES-API, Pos, Scan, Frag:
Max: 146268
[M+H]+= 138 m/z
101.0
121.0
122.0
138.0
156.8
158.8
215.8
m/z

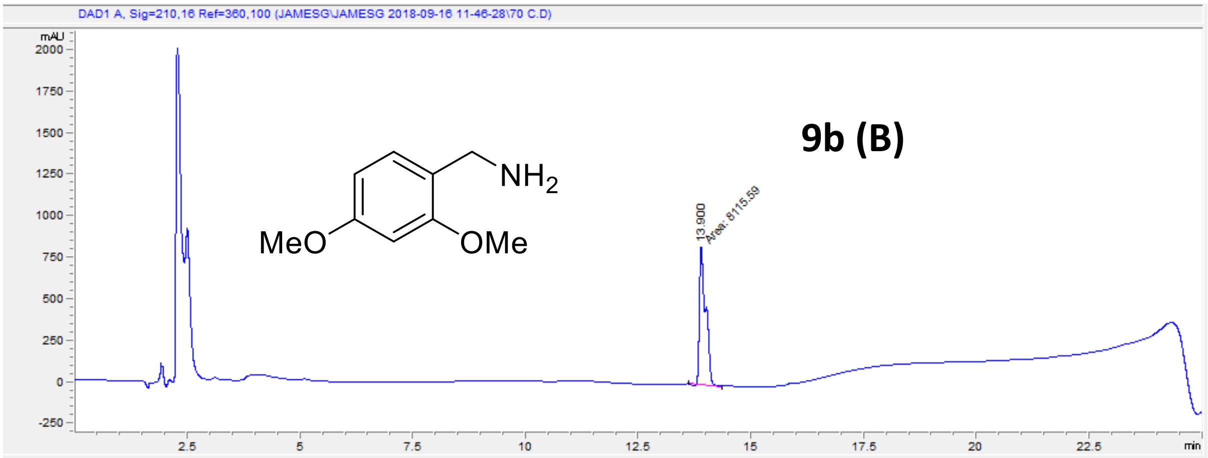
DAD1 A, Sig=210,16 Ref=360,100 (JAMESG\JAMESG 2018-09-16 11-46-28\70 C.D)
9b (B)
NH2
MeO
OMe
13.900
Area: 8115.59
mAU
min

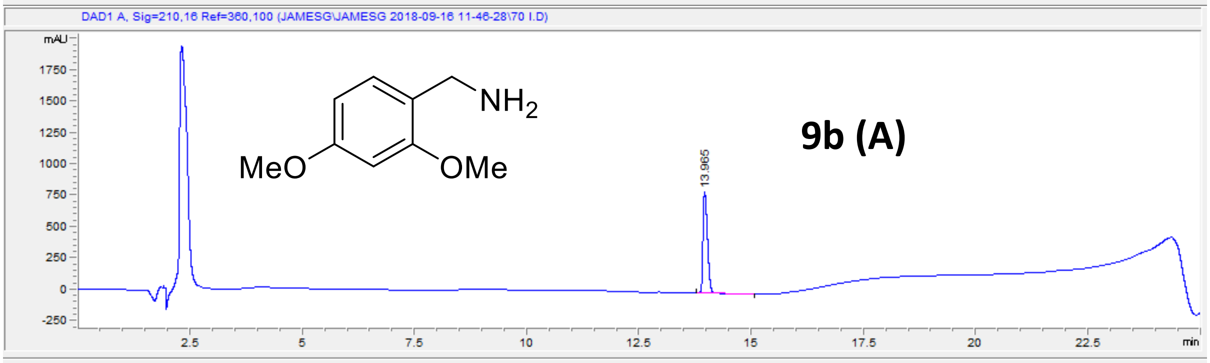
DAD1 A, Sig=210,16 Ref=360,100 (JAMESG\JAMESG 2018-09-16 11-46-28\70 I.D)
9b (A)
NH2
MeO
OMe
13.965
mAU
min

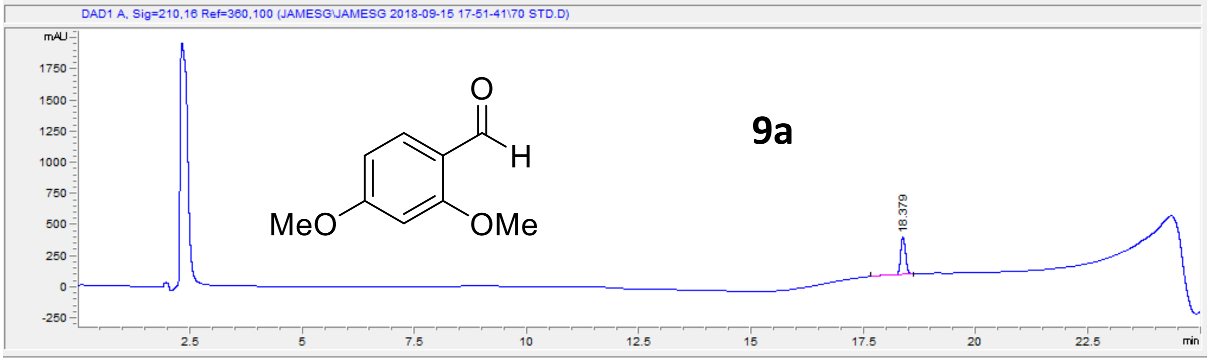
DAD1 A, Sig=210,16 Ref=360,100 (JAMESG\JAMESG 2018-09-15 17-51-41\70 STD.D)
9a
O
H
MeO
OMe
18.379
mAU
min

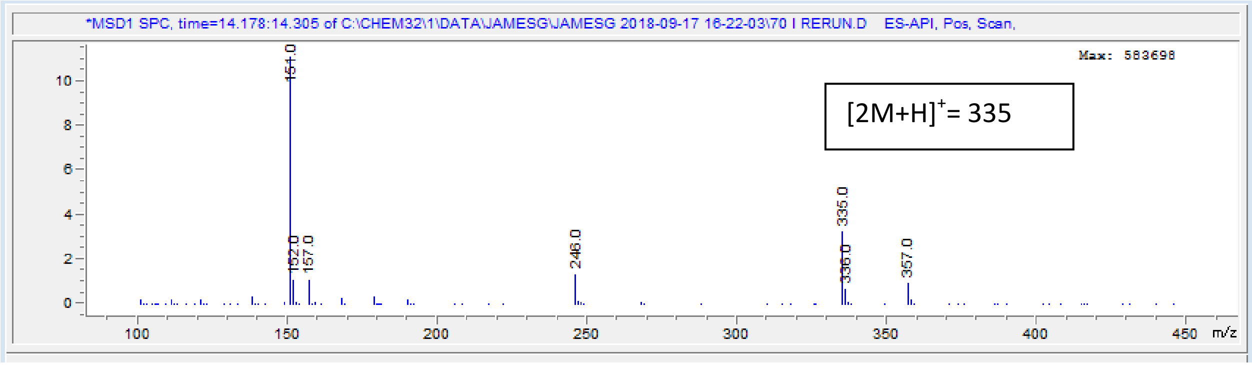
*MSD1 SPC, time=14.178:14.305 of C:\CHEM32\1\DATA\JAMESG\JAMESG 2018-09-17 16-22-03\70 I RERUN.D ES-API, Pos, Scan,
Max: 583698
[2M+H]+= 335
151.0
152.0
157.0
246.0
335.0
336.0
357.0
m/z

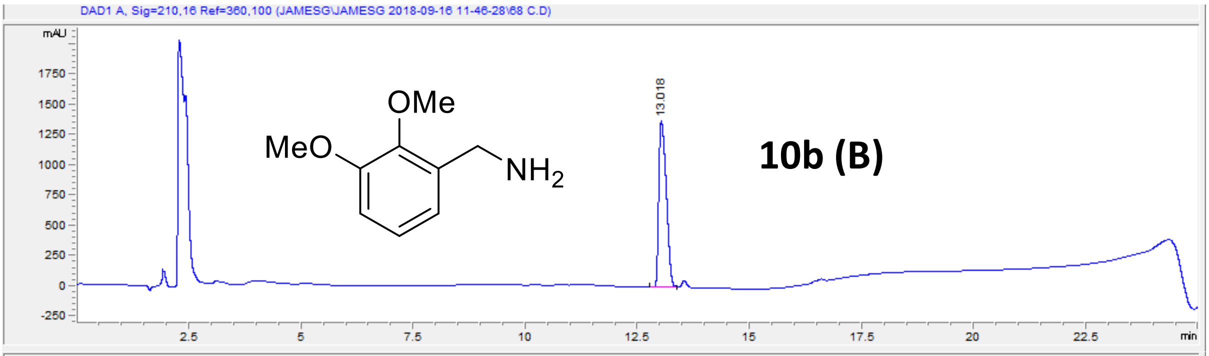
DAD1 A, Sig=210,16 Ref=360,100 (JAMESG\JAMESG 2018-09-16 11-46-28\68 C.D)
mAU
13.018
MeO
OMe
NH2
10b (B)
min

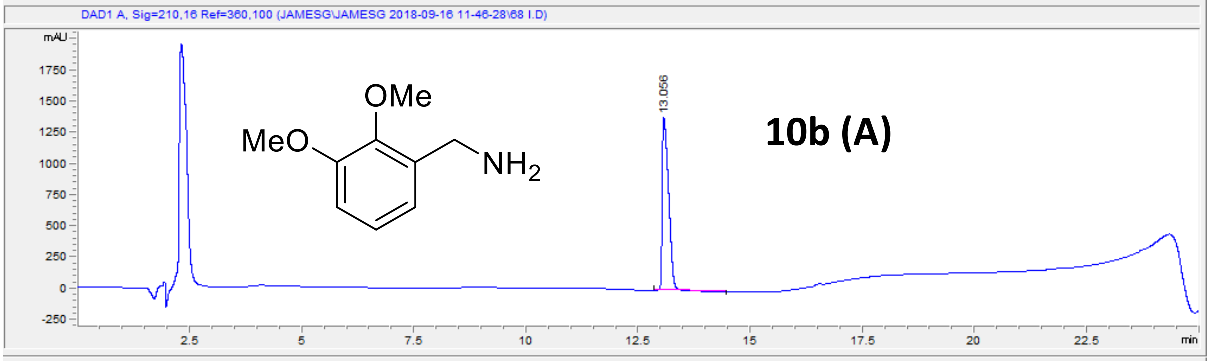
DAD1 A, Sig=210,16 Ref=360,100 (JAMESG\JAMESG 2018-09-16 11-46-28\68 I.D)
mAU
13.056
MeO
OMe
NH2
10b (A)
min

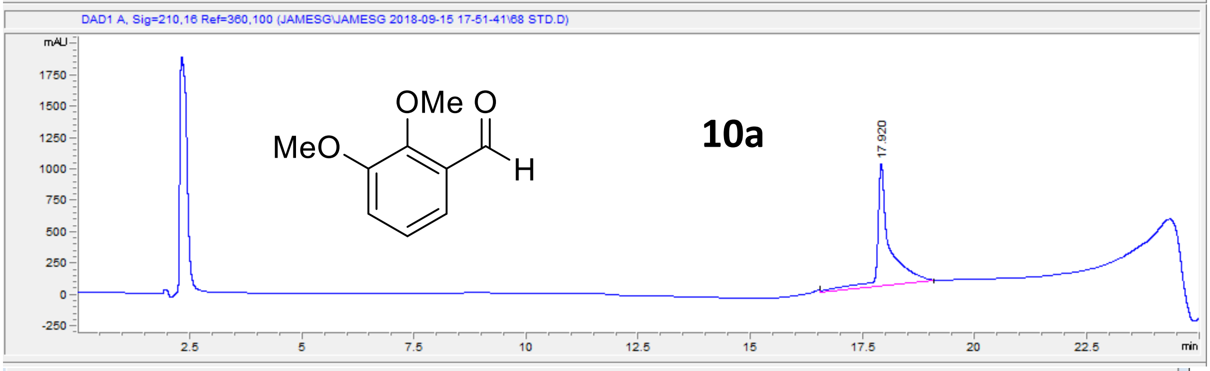
DAD1 A, Sig=210,16 Ref=360,100 (JAMESG\JAMESG 2018-09-15 17-51-41\68 STD.D)
mAU
17.920
MeO
OMe
O
H
10a
min

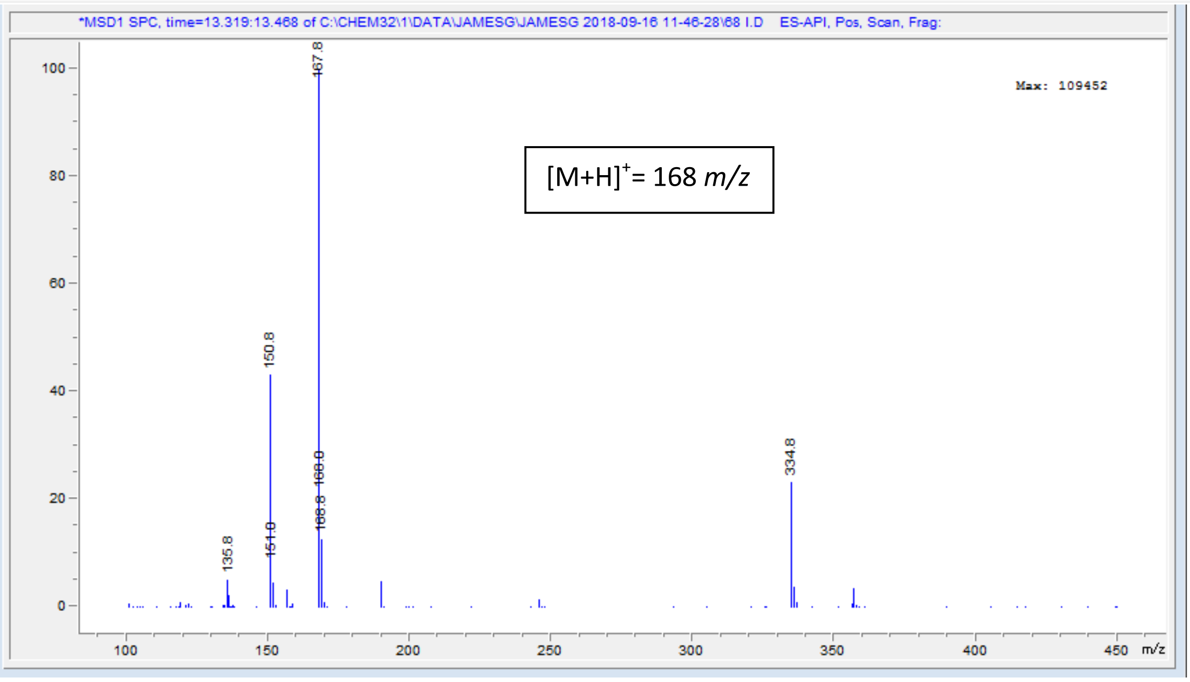
*MSD1 SPC, time=13.319:13.468 of C:\CHEM32\1\DATA\JAMESG\JAMESG 2018-09-16 11-46-28\68 I.D ES-API, Pos, Scan, Frag:
Max: 109452
[M+H]+= 168 m/z
167.8
150.8
168.8
169.0
151.0
135.8
334.8
m/z

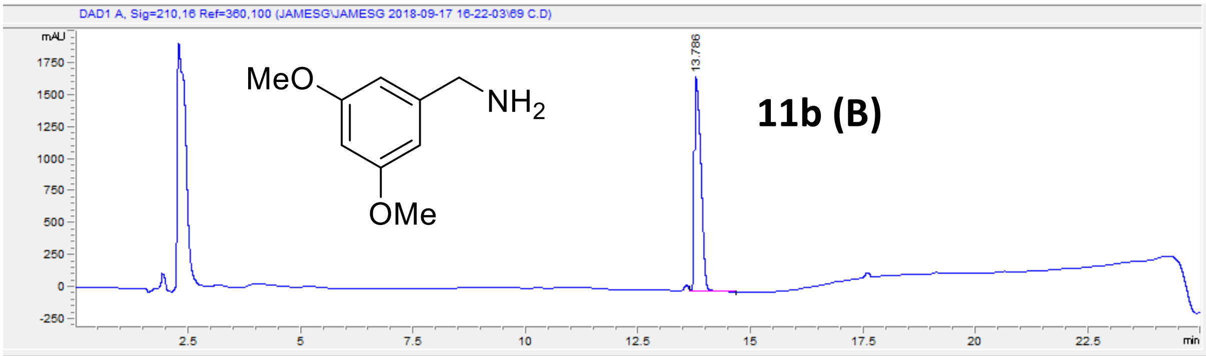
DAD1 A, Sig=210,16 Ref=360,100 (JAMESG\JAMESG 2018-09-17 16-22-03\69 C.D)
MeO
NH2
OMe
11b (B)
13.786
mAU
min

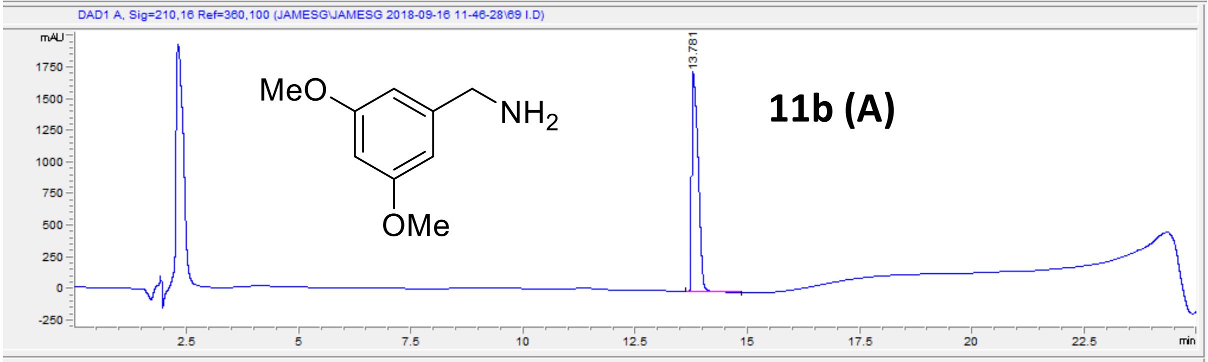
DAD1 A, Sig=210,16 Ref=360,100 (JAMESG\JAMESG 2018-09-16 11-46-28\69 I.D)
MeO
NH2
OMe
11b (A)
13.781
mAU
min

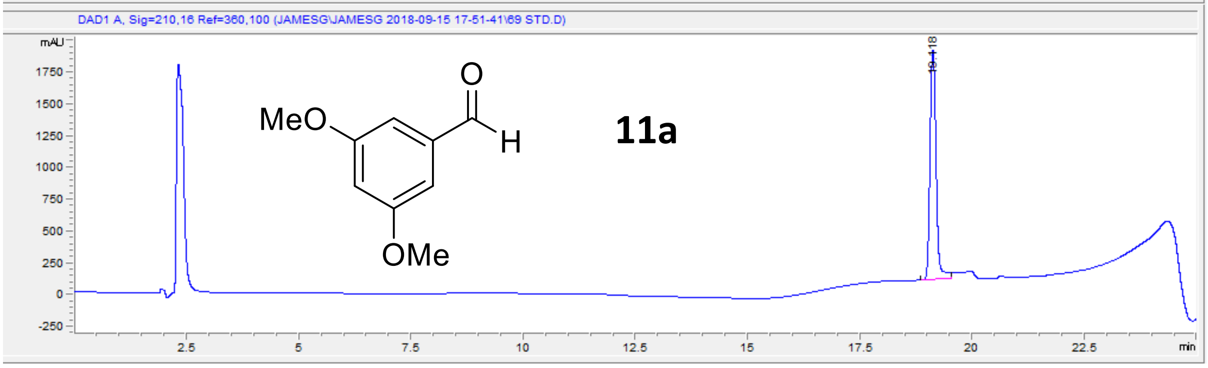
DAD1 A, Sig=210,16 Ref=360,100 (JAMESG\JAMESG 2018-09-15 17-51-41\69 STD.D)
MeO
O
H
OMe
11a
mAU
min

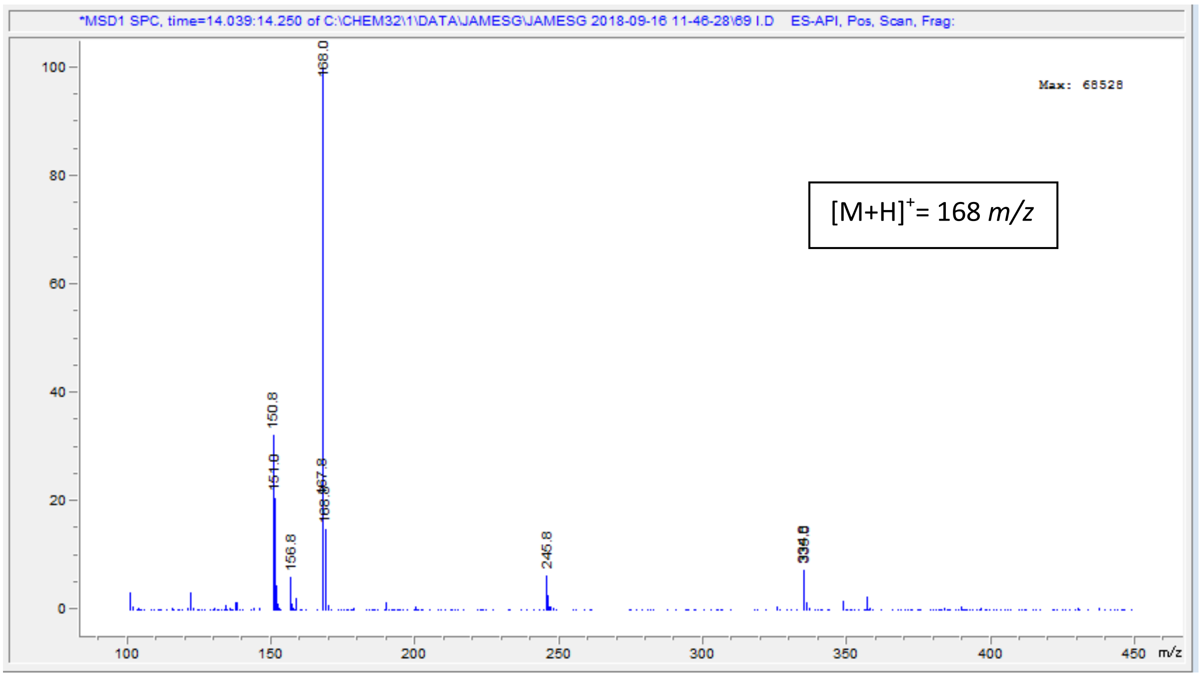
*MSD1 SPC, time=14.039:14.250 of C:\CHEM32\1\DATA\JAMESG\JAMESG 2018-09-16 11-46-28\69 I.D ES-API, Pos, Scan, Frag:
Max: 68528
[M+H]+= 168 m/z
168.0
150.8
156.8
245.8
m/z

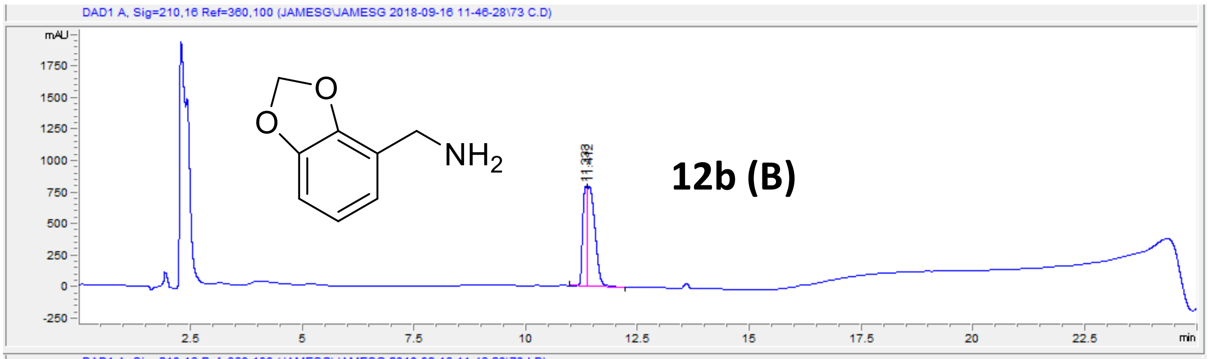
DAD1 A, Sig=210,16 Ref=360,100 (JAMESG\JAMESG 2018-09-16 11-46-28\73 C.D)
mAU
NH2
12b (B)
min

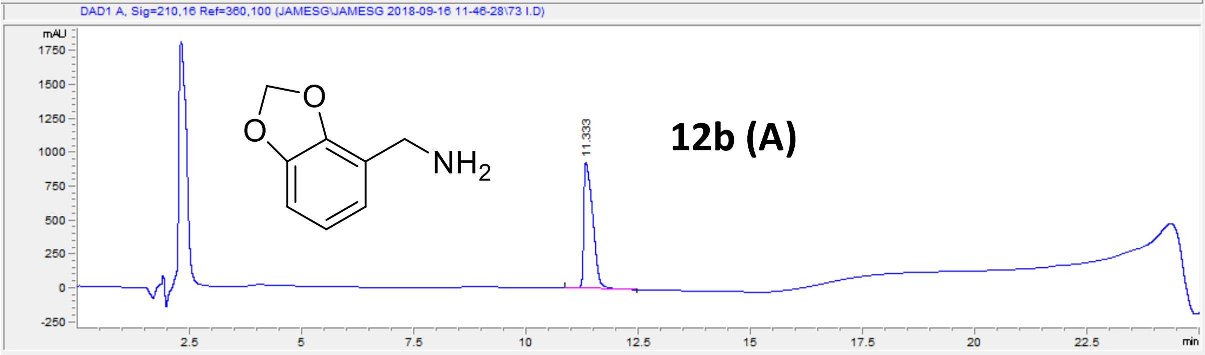
DAD1 A, Sig=210,16 Ref=360,100 (JAMESG\JAMESG 2018-09-16 11-46-28\73 I.D)
mAU
11.333
NH2
12b (A)
min

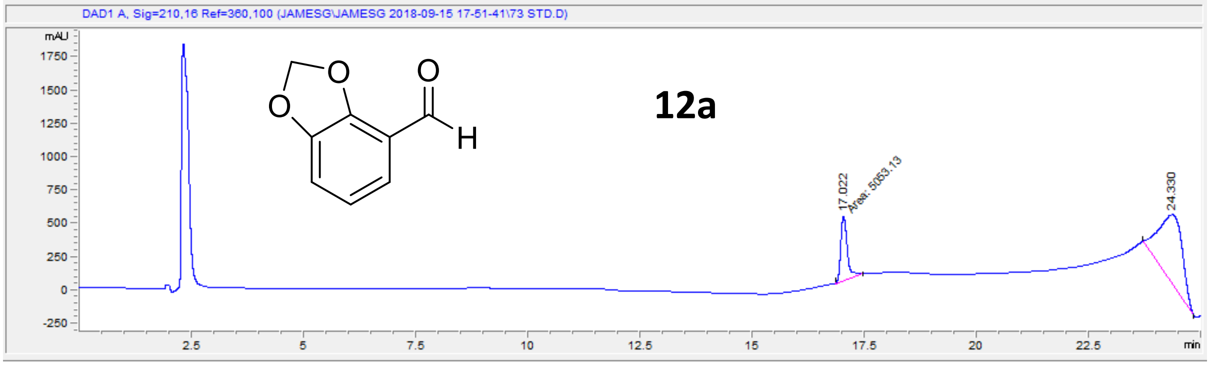
DAD1 A, Sig=210,16 Ref=360,100 (JAMESG\JAMESG 2018-09-15 17-51-41\73 STD.D)
mAU
12a
17.022
Area: 5053.13
24.330
min

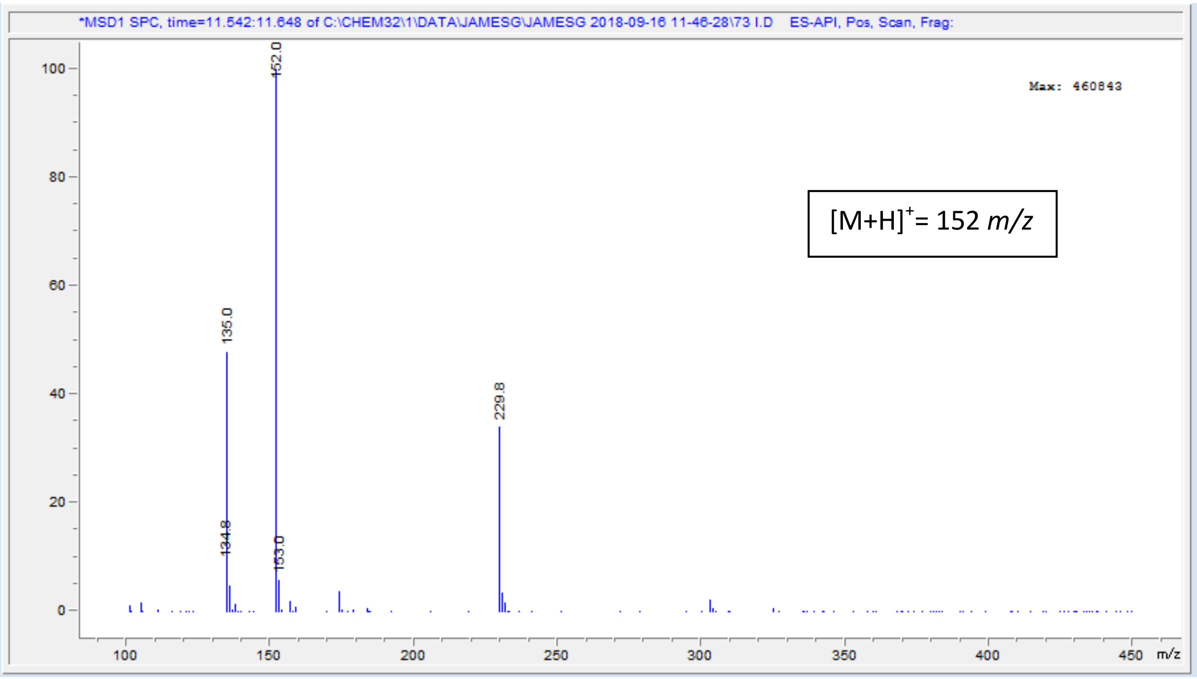
*MSD1 SPC, time=11.542:11.648 of C:\CHEM32\1\DATA\JAMESG\JAMESG 2018-09-16 11-46-28\73 I.D ES-API, Pos, Scan, Frag:
Max: 460843
[M+H]+= 152 m/z
152.0
135.0
229.8
134.8
153.0
m/z

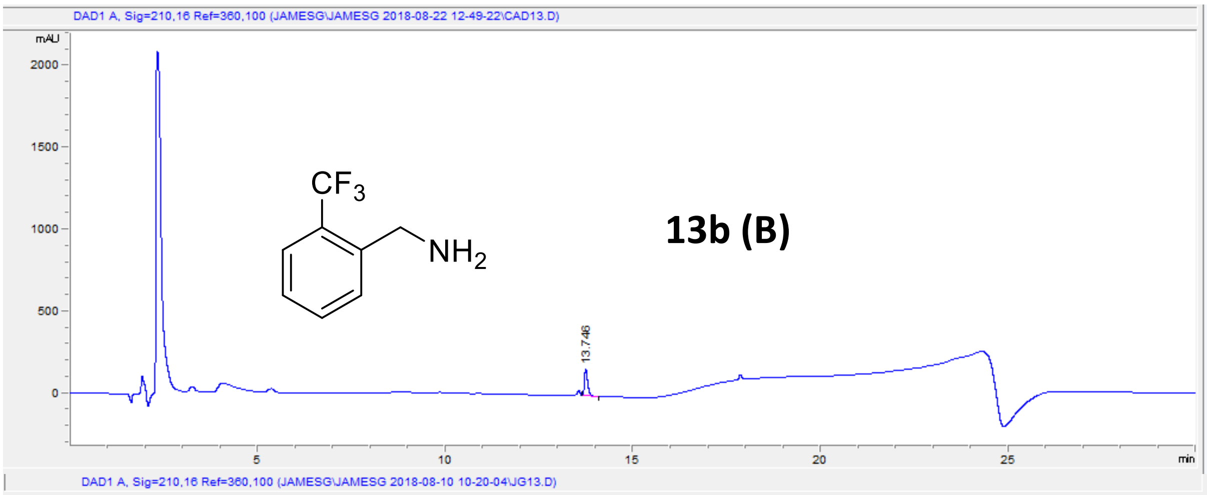
DAD1 A, Sig=210,16 Ref=360,100 (JAMESG\JAMESG 2018-08-22 12-49-22\CAD13.D)
mAU
CF3
NH2
13b (B)
13.746
min

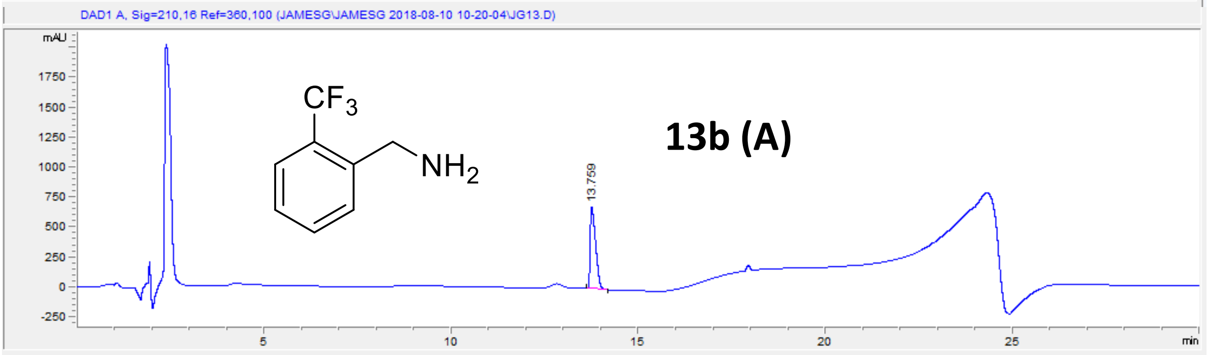
DAD1 A, Sig=210,16 Ref=360,100 (JAMESG\JAMESG 2018-08-10 10-20-04\JG13.D)
mAU
CF3
NH2
13b (A)
13.759
min

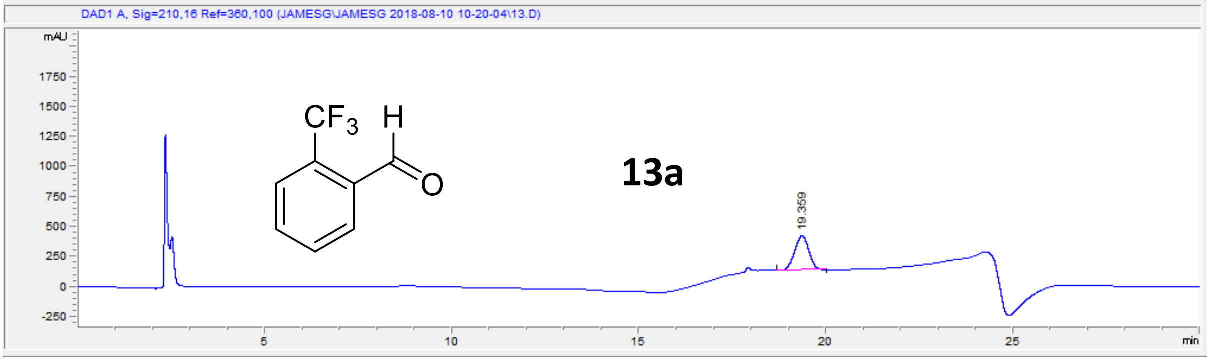
DAD1 A, Sig=210,16 Ref=360,100 (JAMESG\JAMESG 2018-08-10 10-20-04\13.D)
mAU
CF3
H
O
13a
19.359
min

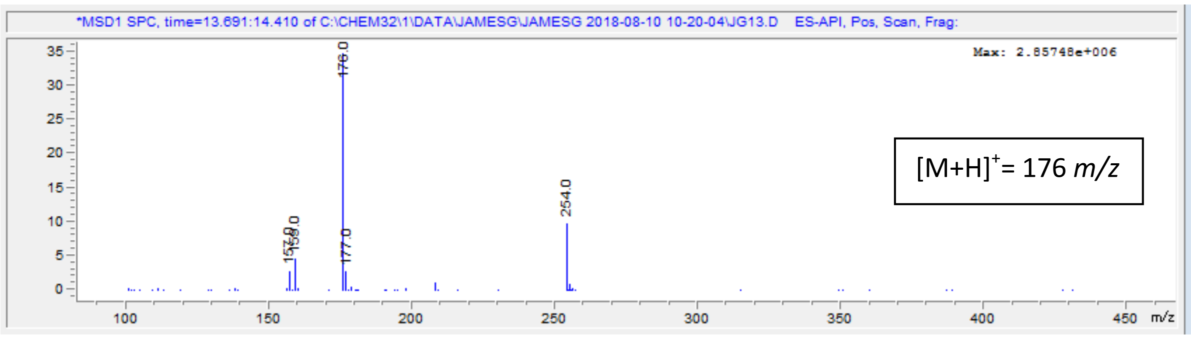
*MSD1 SPC, time=13.691:14.410 of C:\CHEM32\1\DATA\JAMESG\JAMESG 2018-08-10 10-20-04\JG13.D ES-API, Pos, Scan, Frag:
Max: 2.85748e+006
176.0
254.0
177.0
[M+H]+= 176 m/z
m/z

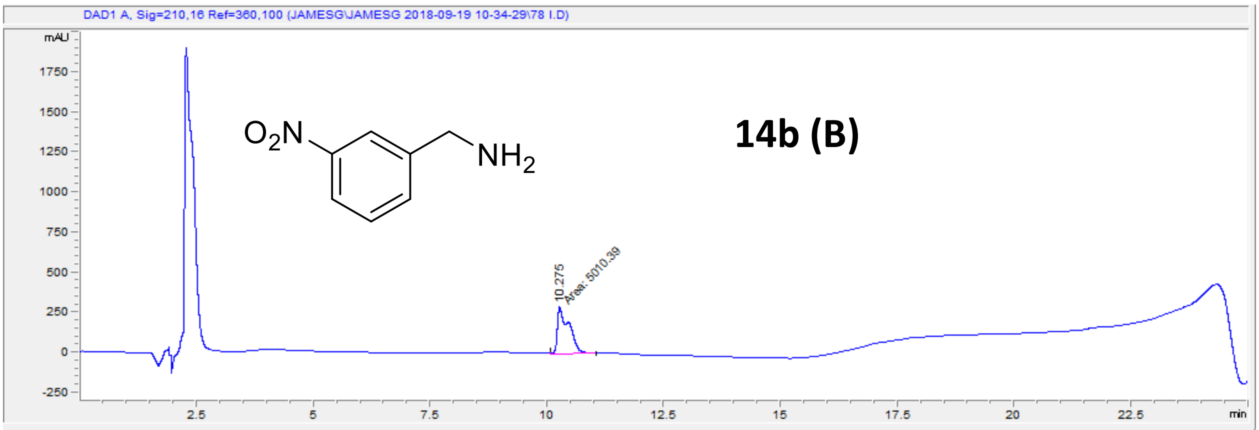
DAD1 A, Sig=210,16 Ref=360,100 (JAMESG\JAMESG 2018-09-19 10-34-29\78 I.D)
14b (B)
O2N
NH2
10.275
Area: 5010.39
mAU
min

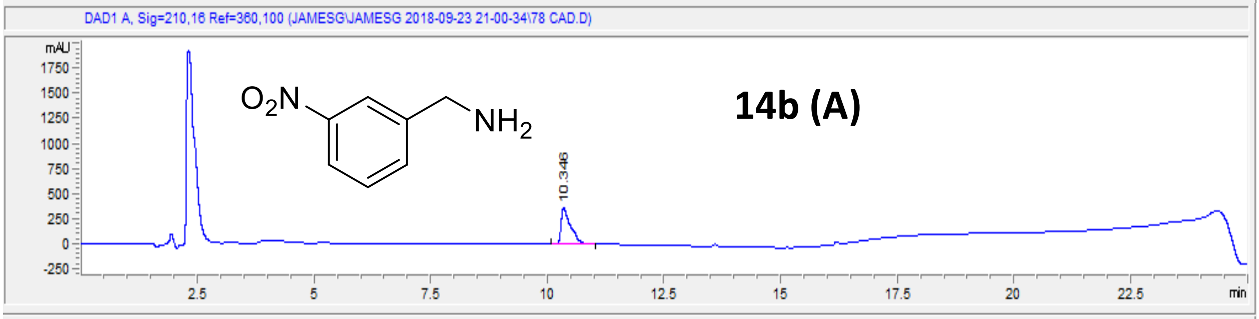
DAD1 A, Sig=210,16 Ref=360,100 (JAMESG\JAMESG 2018-09-23 21-00-34\78 CAD.D)
14b (A)
O2N
NH2
10.346
mAU
min

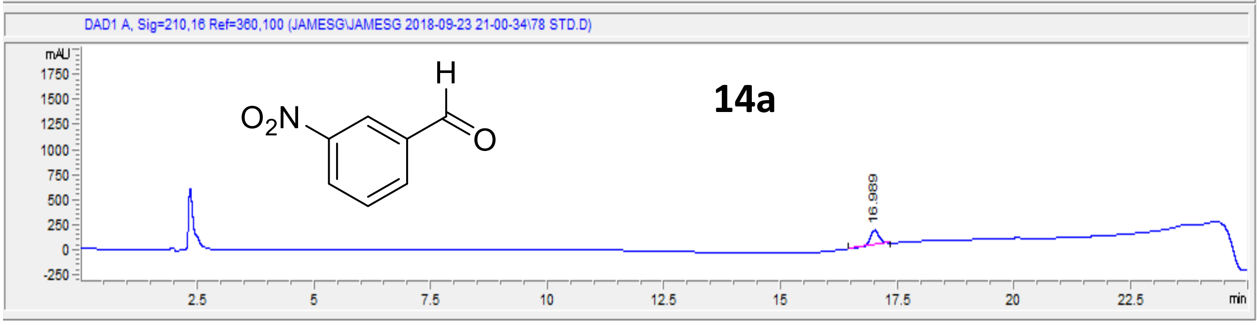
DAD1 A, Sig=210,16 Ref=360,100 (JAMESG\JAMESG 2018-09-23 21-00-34\78 STD.D)
14a
O2N
H
O
16.989
mAU
min

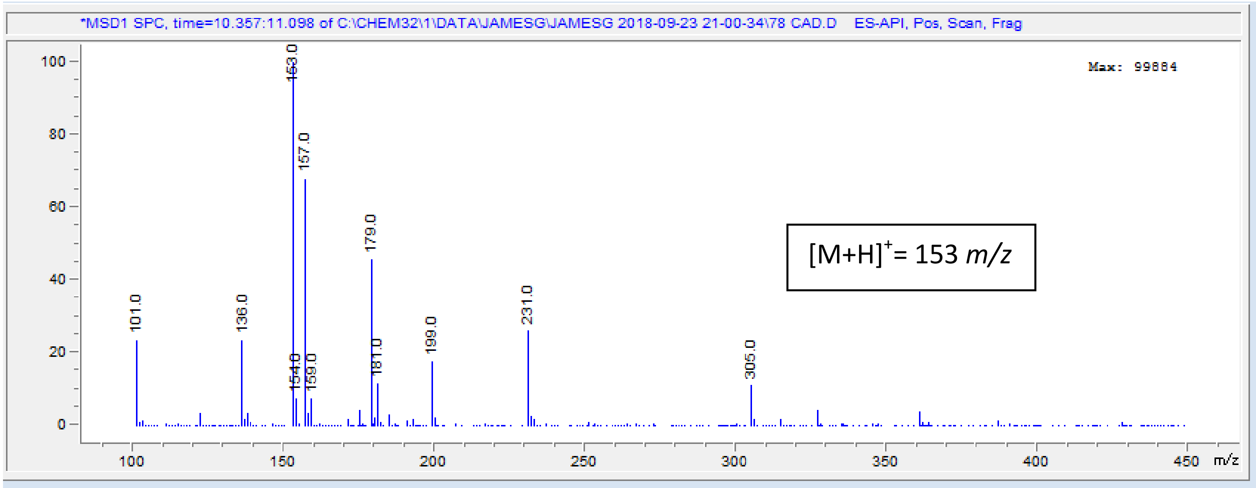
*MSD1 SPC, time=10.357:11.098 of C:\CHEM32\1\DATA\JAMESG\JAMESG 2018-09-23 21-00-34\78 CAD.D ES-API, Pos, Scan, Frag
Max: 99884
[M+H]+= 153 m/z
153.0
157.0
179.0
101.0
136.0
154.0
159.0
181.0
199.0
231.0
305.0
m/z

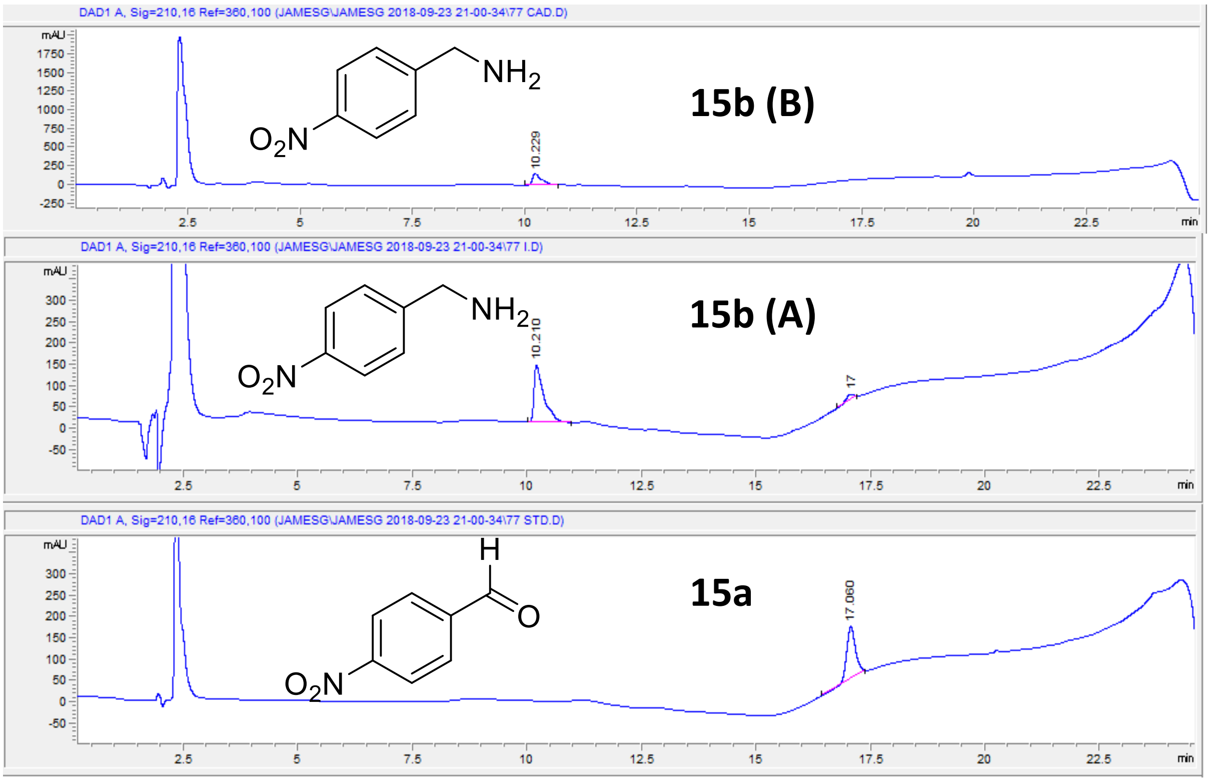

DAD1 A, Sig=210,16 Ref=360,100 (JAMESG\JAMESG 2018-09-23 21-00-34\77 CAD.D)
15b (B)
DAD1 A, Sig=210,16 Ref=360,100 (JAMESG\JAMESG 2018-09-23 21-00-34\77 I.D)
15b (A)
DAD1 A, Sig=210,16 Ref=360,100 (JAMESG\JAMESG 2018-09-23 21-00-34\77 STD.D)
15a

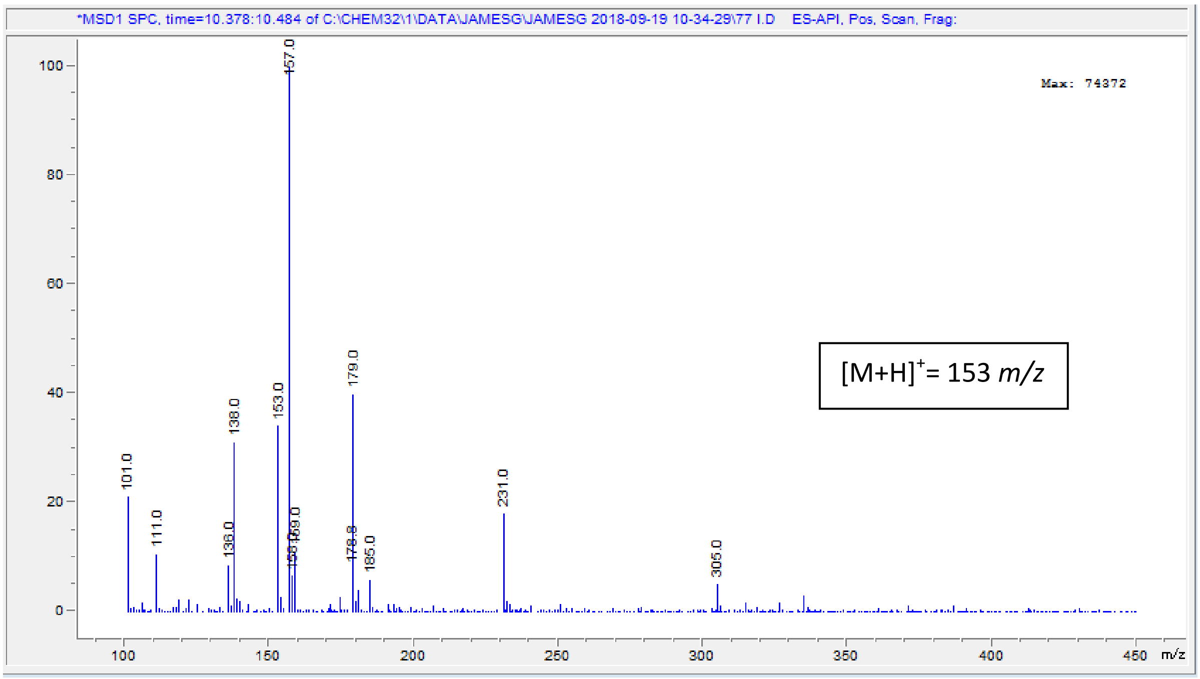

*MSD1 SPC, time=10.378:10.484 of C:\CHEM32\1\DATA\JAMESG\JAMESG 2018-09-19 10-34-29\77 I.D ES-API, Pos, Scan, Frag:
Max: 74372
[M+H]+= 153 m/z

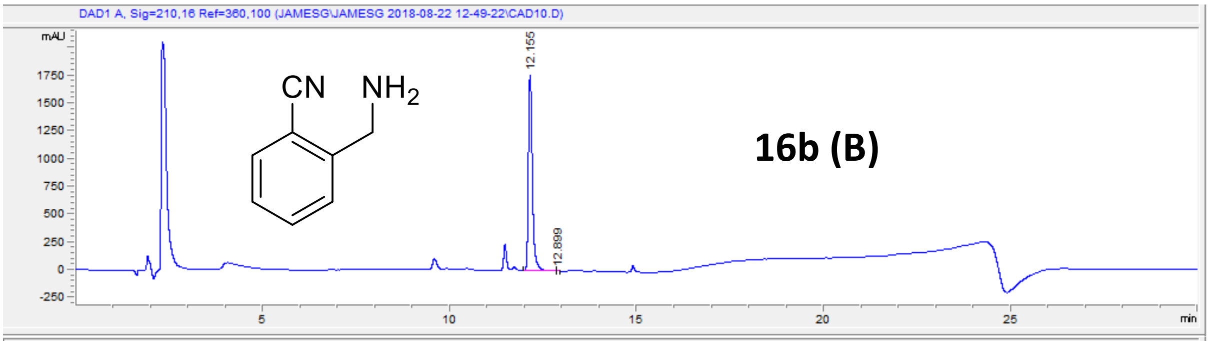
DAD1 A, Sig=210,16 Ref=360,100 (JAMESG\JAMESG 2018-08-22 12-49-22\CAD10.D)
CN NH2
16b (B)
12.155
12.899
mAU
min

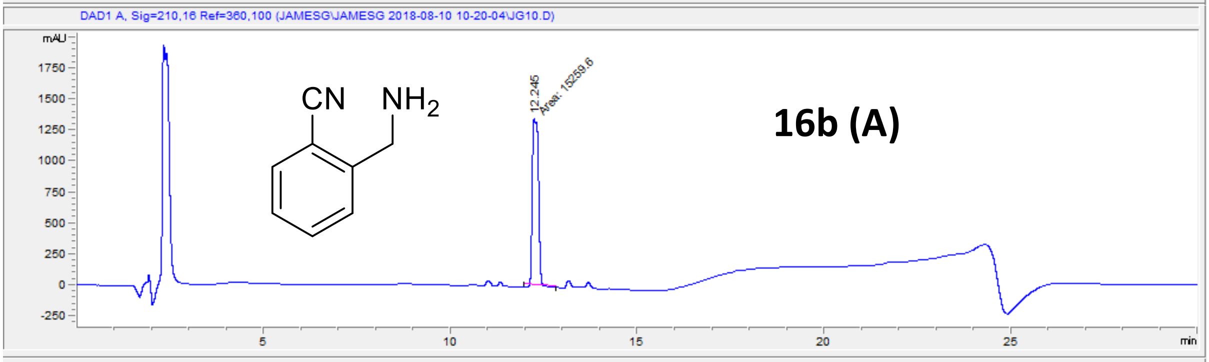
DAD1 A, Sig=210,16 Ref=360,100 (JAMESG\JAMESG 2018-08-10 10-20-04\JG10.D)
CN NH2
16b (A)
12.245
Area: 15259.6
mAU
min

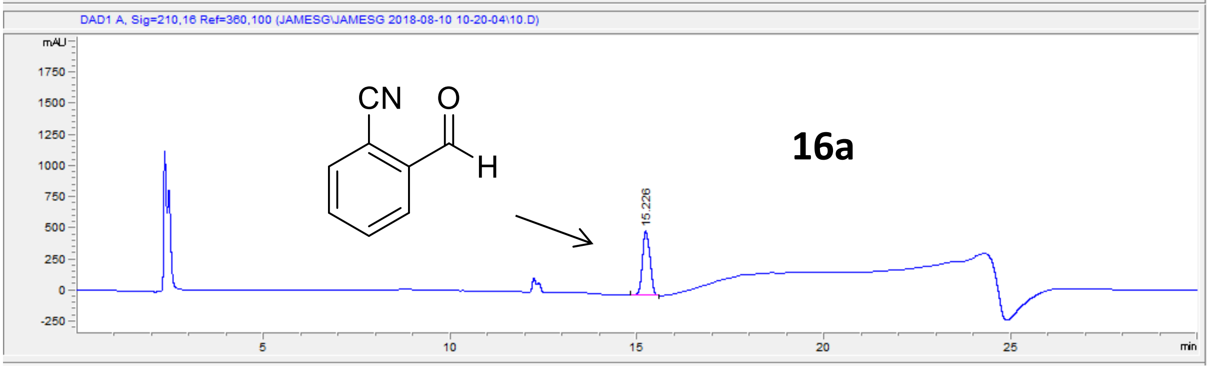
DAD1 A, Sig=210,16 Ref=360,100 (JAMESG\JAMESG 2018-08-10 10-20-04\10.D)
CN O
H
16a
15.226
mAU
min

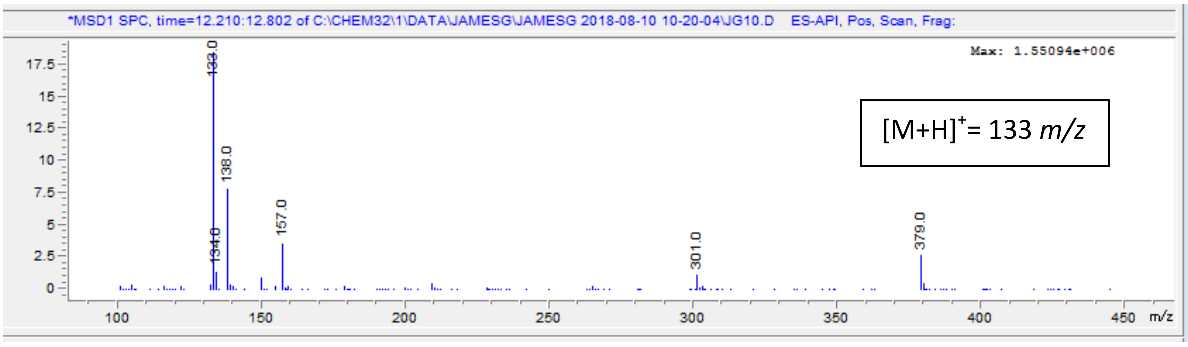
*MSD1 SPC, time=12.210:12.802 of C:\CHEM32\1\DATA\JAMESG\JAMESG 2018-08-10 10-20-04\JG10.D ES-API, Pos, Scan, Frag:
Max: 1.55094e+006
[M+H]+= 133 m/z
133.0
134.0
138.0
157.0
301.0
379.0
m/z

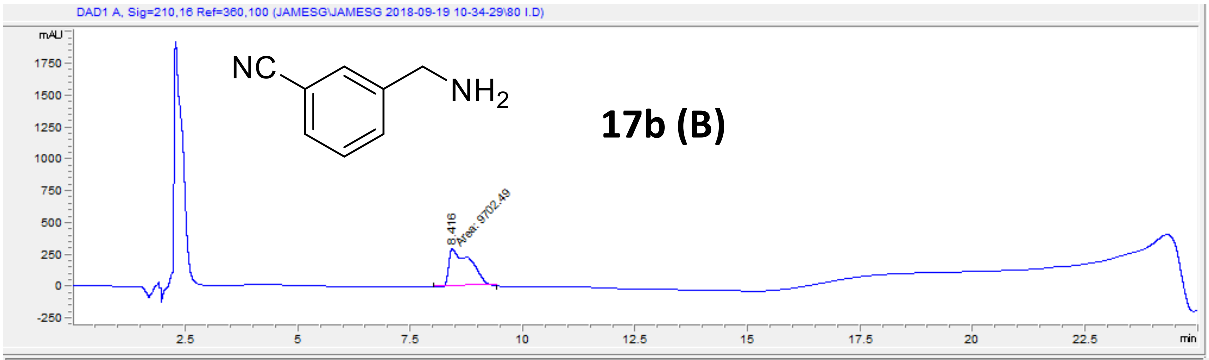
DAD1 A, Sig=210,16 Ref=360,100 (JAMESG\JAMESG 2018-09-19 10-34-29\80 I.D)
mAU
NC
NH_2
17b (B)
8.416
Area: 9702.49
min

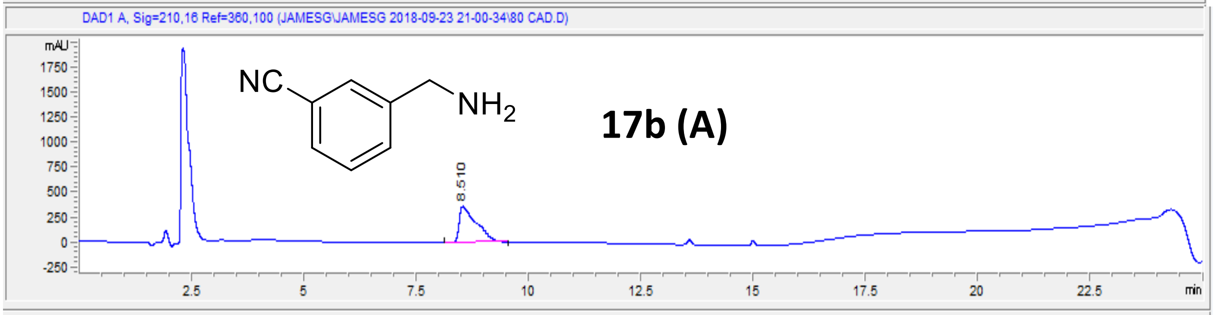
DAD1 A, Sig=210,16 Ref=360,100 (JAMESG\JAMESG 2018-09-23 21-00-34\80 CAD.D)
mAU
NC
NH_2
17b (A)
8.510
min

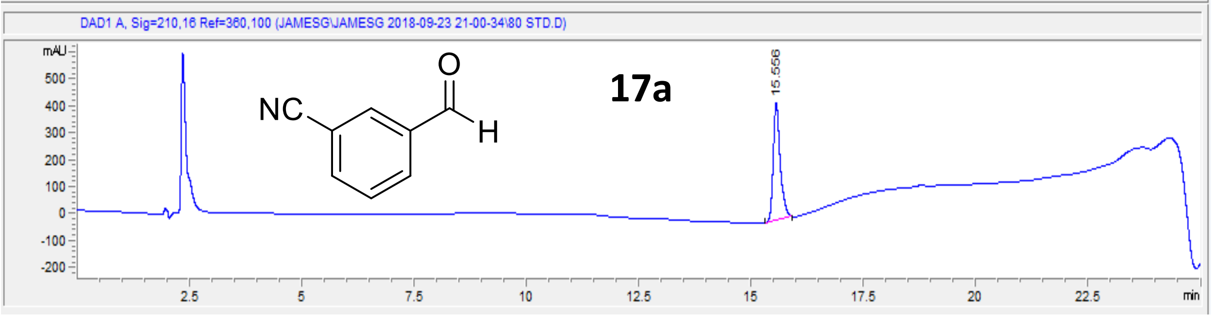
DAD1 A, Sig=210,16 Ref=360,100 (JAMESG\JAMESG 2018-09-23 21-00-34\80 STD.D)
mAU
NC
O
H
17a
15.556
min

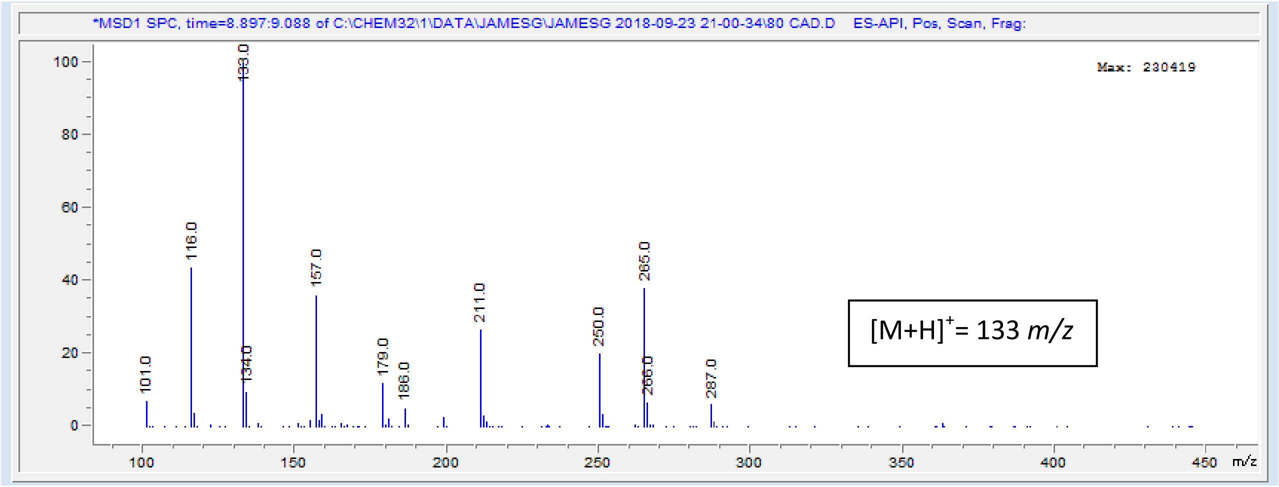
*MSD1 SPC, time=8.897:9.088 of C:\CHEM32\1\DATA\JAMESG\JAMESG 2018-09-23 21-00-34\80 CAD.D ES-API, Pos, Scan, Frag:
Max: 230419
101.0
116.0
133.0
134.0
157.0
179.0
186.0
211.0
250.0
265.0
266.0
287.0
[M+H]$^+$= 133 *m/z*
m/z

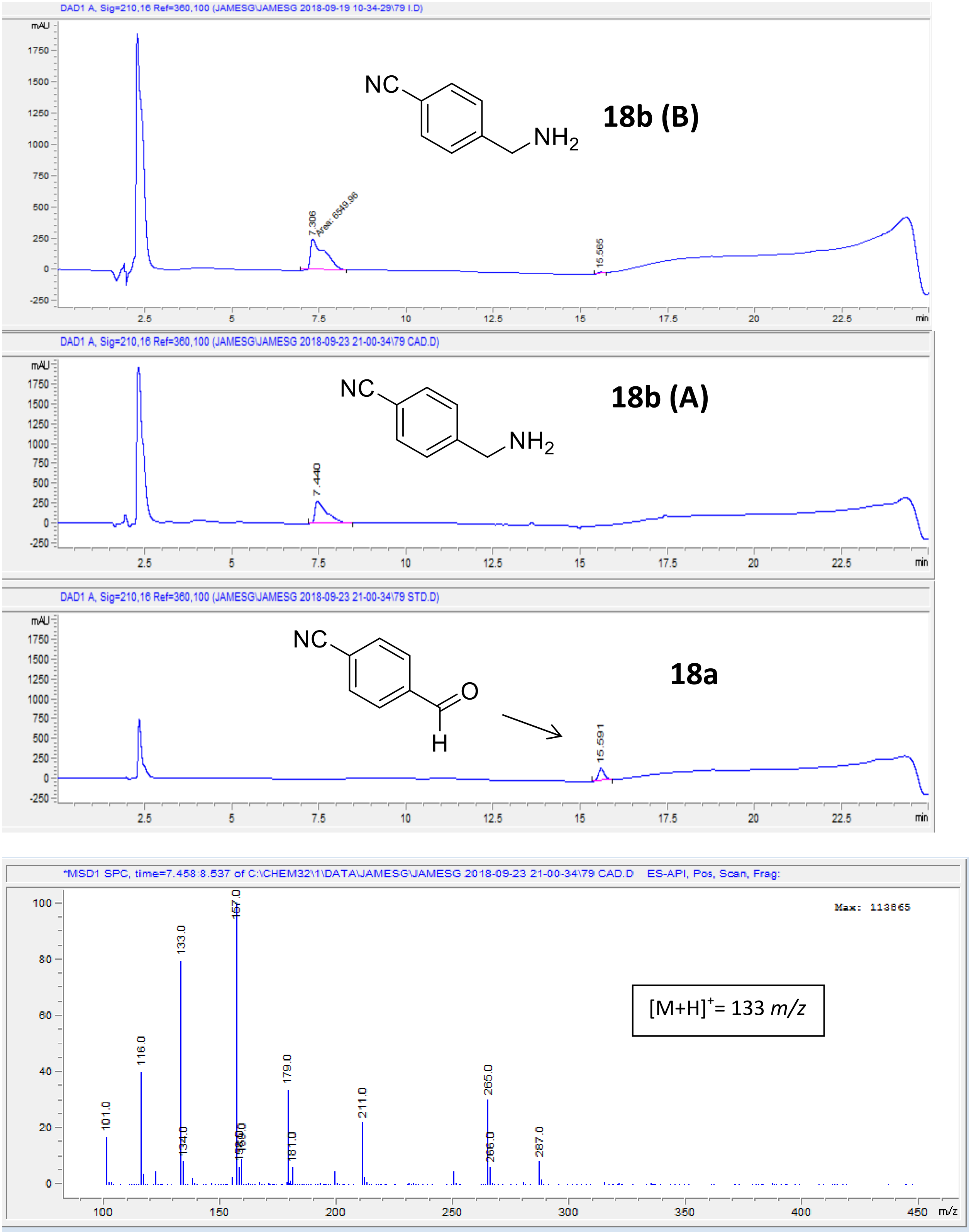
DAD1 A, Sig=210,16 Ref=360,100 (JAMESG\JAMESG 2018-09-19 10-34-29\79 I.D)
NC
NH2
18b (B)
7.306
Area: 6549.96
15.565
DAD1 A, Sig=210,16 Ref=360,100 (JAMESG\JAMESG 2018-09-23 21-00-34\79 CAD.D)
NC
NH2
18b (A)
7.440
DAD1 A, Sig=210,16 Ref=360,100 (JAMESG\JAMESG 2018-09-23 21-00-34\79 STD.D)
NC
O
H
18a
15.591
mAU
min
*MSD1 SPC, time=7.458:8.537 of C:\CHEM32\1\DATA\JAMESG\JAMESG 2018-09-23 21-00-34\79 CAD.D ES-API, Pos, Scan, Frag:
Max: 113865
[M+H]+= 133 m/z
101.0
116.0
133.0
134.0
157.0
179.0
181.0
211.0
265.0
266.0
287.0
m/z

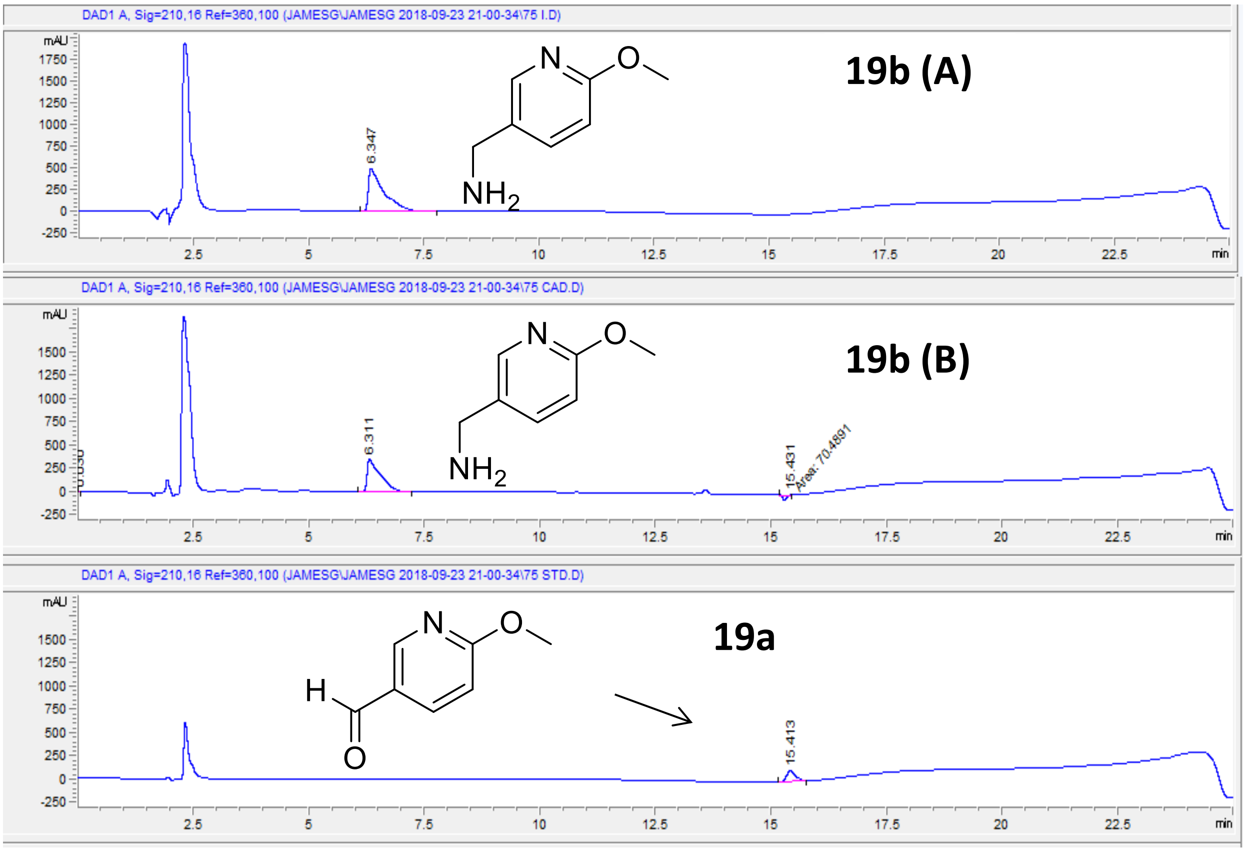
DAD1 A, Sig=210,16 Ref=360,100 (JAMESG\JAMESG 2018-09-23 21-00-34\75 I.D)
19b (A)
6.347
NH2
DAD1 A, Sig=210,16 Ref=360,100 (JAMESG\JAMESG 2018-09-23 21-00-34\75 CAD.D)
19b (B)
6.311
15.431
Area: 70.4891
DAD1 A, Sig=210,16 Ref=360,100 (JAMESG\JAMESG 2018-09-23 21-00-34\75 STD.D)
19a
15.413
mAU
min

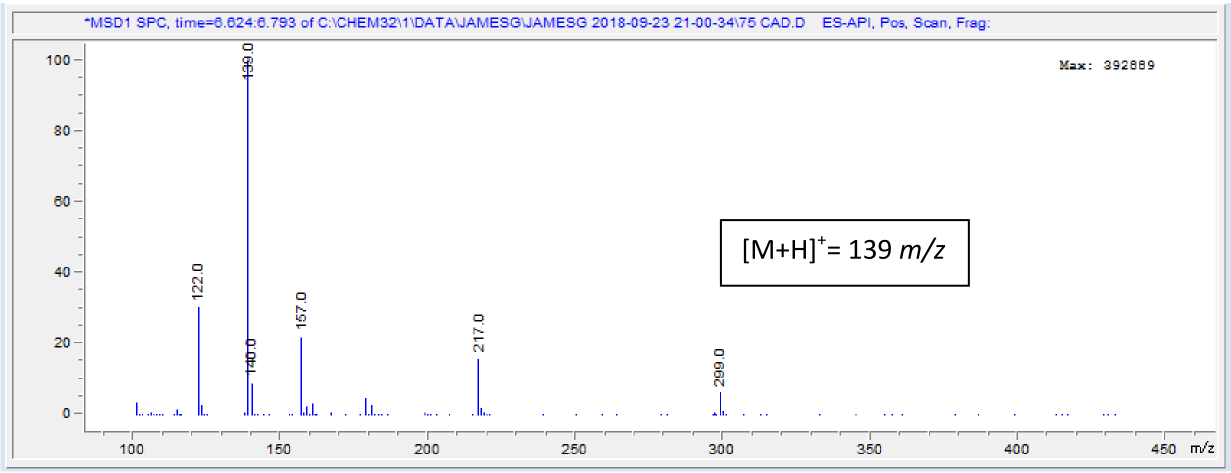
*MSD1 SPC, time=6.624:6.793 of C:\CHEM32\1\DATA\JAMESG\JAMESG 2018-09-23 21-00-34\75 CAD.D ES-API, Pos, Scan, Frag:
Max: 392889
139.0
122.0
140.0
157.0
217.0
299.0
[M+H]+= 139 m/z
m/z

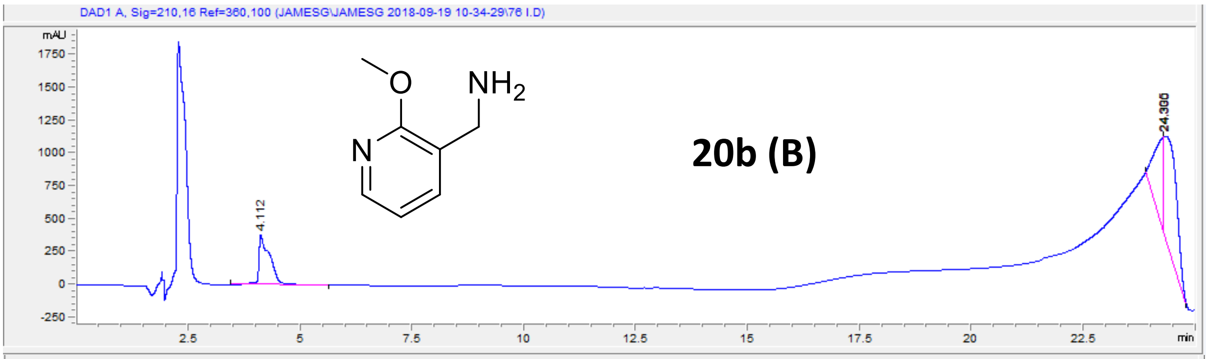
DAD1 A, Sig=210,16 Ref=360,100 (JAMESG\JAMESG 2018-09-19 10-34-29\76 I.D)
mAU
4.112
24.366
min
NH2
O
N
20b (B)

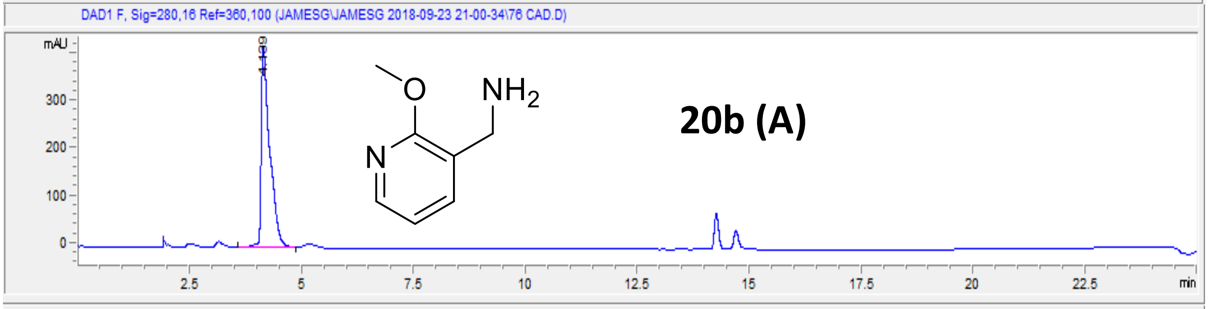
DAD1 F, Sig=280,16 Ref=360,100 (JAMESG\JAMESG 2018-09-23 21-00-34\76 CAD.D)
mAU
min
NH2
O
N
20b (A)

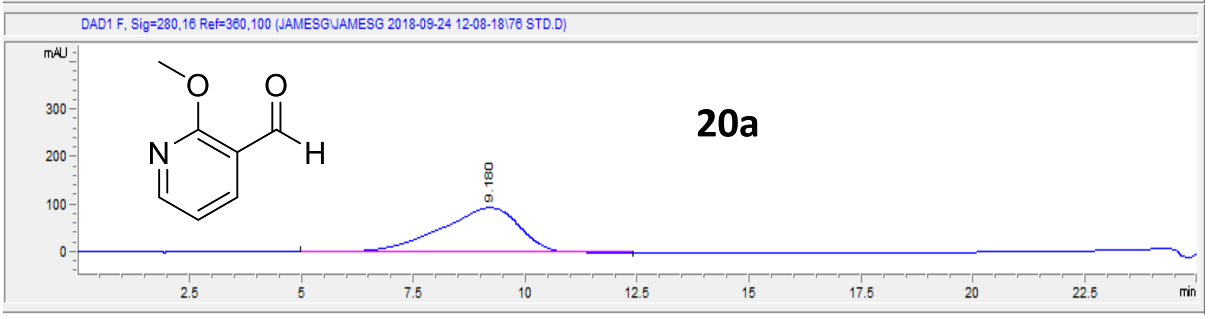
DAD1 F, Sig=280,16 Ref=360,100 (JAMESG\JAMESG 2018-09-24 12-08-18\76 STD.D)
mAU
9.180
min
O
O
N
H
20a

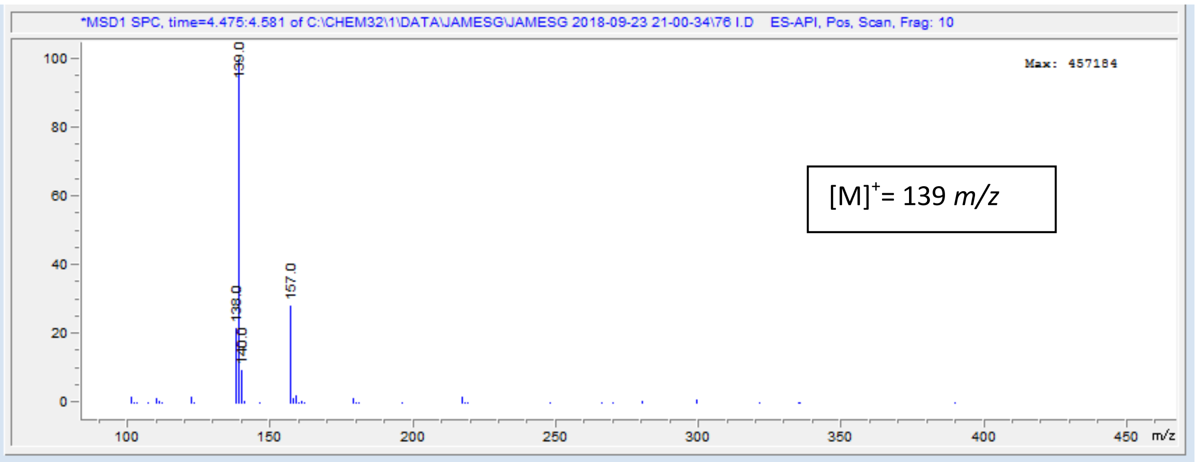
*MSD1 SPC, time=4.475:4.581 of C:\CHEM32\1\DATA\JAMESG\JAMESG 2018-09-23 21-00-34\76 I.D ES-API, Pos, Scan, Frag: 10
Max: 457184
139.0
138.0
140.0
157.0
m/z
[M]+= 139 m/z

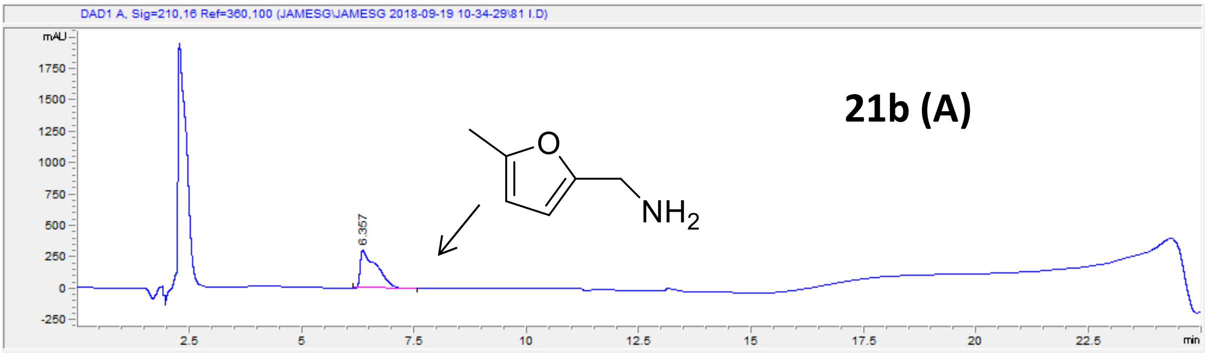
DAD1 A, Sig=210,16 Ref=360,100 (JAMESG\JAMESG 2018-09-19 10-34-29\81 I.D)
21b (A)
O
NH_2
6.357
mAU
min

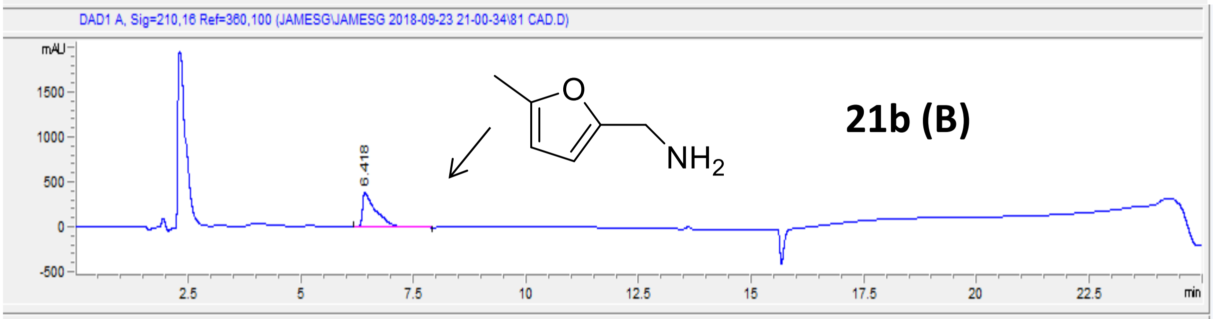
DAD1 A, Sig=210,16 Ref=360,100 (JAMESG\JAMESG 2018-09-23 21-00-34\81 CAD.D)
21b (B)
O
NH_2
6.418
mAU
min

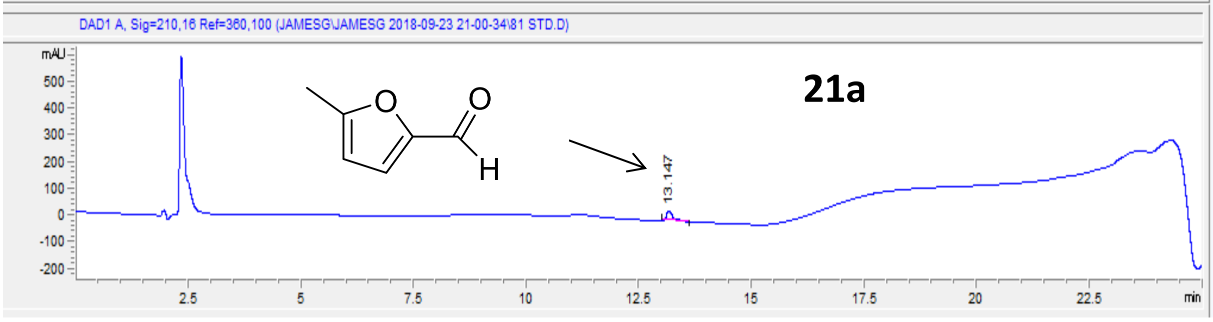
DAD1 A, Sig=210,16 Ref=360,100 (JAMESG\JAMESG 2018-09-23 21-00-34\81 STD.D)
21a
O
O
H
13.147
mAU
min

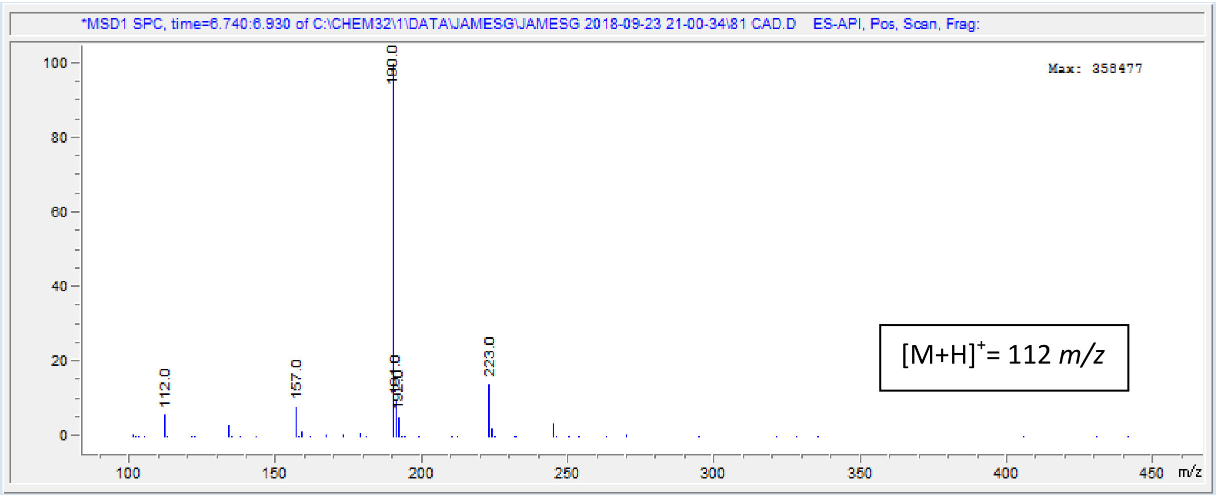
*MSD1 SPC, time=6.740:6.930 of C:\CHEM32\1\DATA\JAMESG\JAMESG 2018-09-23 21-00-34\81 CAD.D ES-API, Pos, Scan, Frag:
Max: 358477
190.0
112.0
157.0
223.0
[M+H]$^+$= 112 *m/z*
m/z

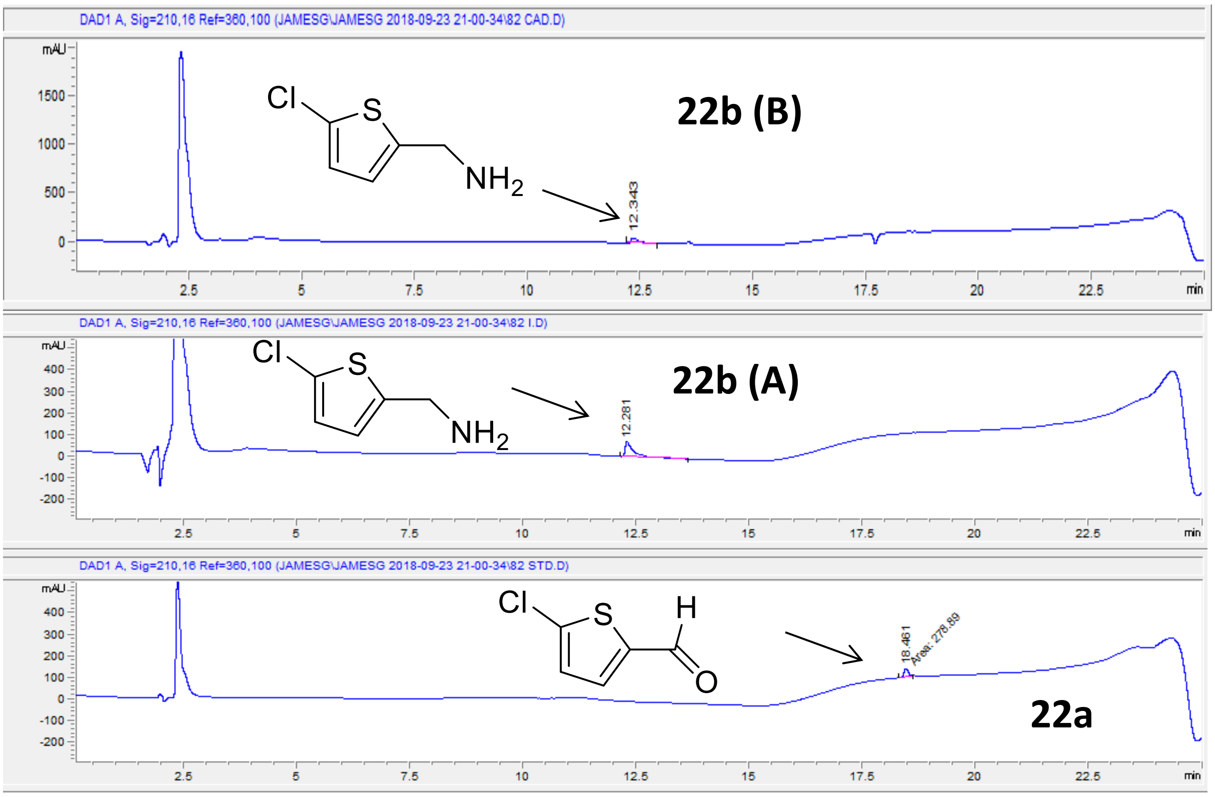
DAD1 A, Sig=210,16 Ref=360,100 (JAMESG\JAMESG 2018-09-23 21-00-34\82 CAD.D)
22b (B)
12.343
DAD1 A, Sig=210,16 Ref=360,100 (JAMESG\JAMESG 2018-09-23 21-00-34\82 I.D)
22b (A)
12.281
DAD1 A, Sig=210,16 Ref=360,100 (JAMESG\JAMESG 2018-09-23 21-00-34\82 STD.D)
18.461
Area: 278.89
22a
mAU
min

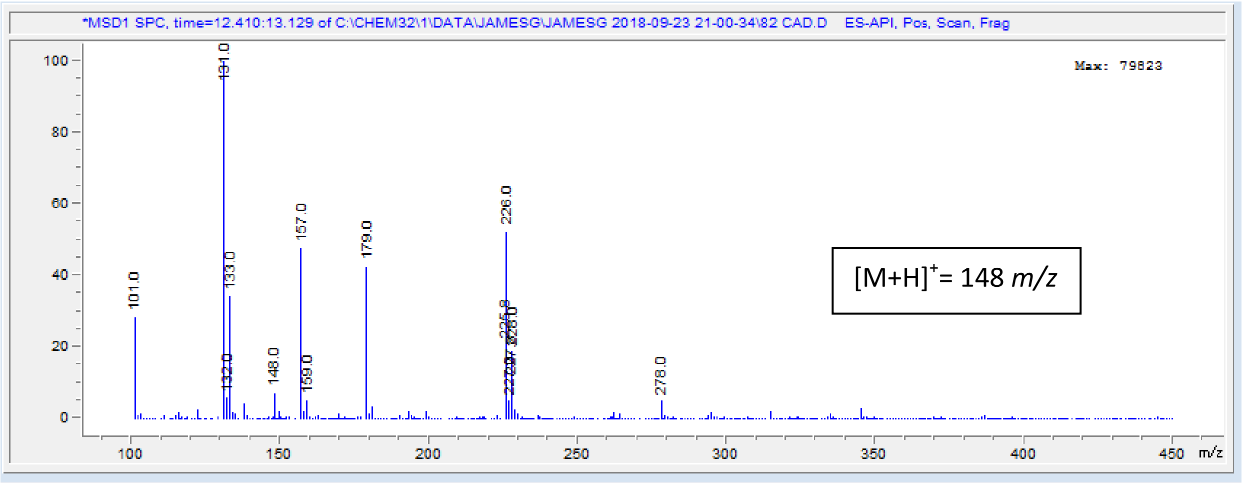
*MSD1 SPC, time=12.410:13.129 of C:\CHEM32\1\DATA\JAMESG\JAMESG 2018-09-23 21-00-34\82 CAD.D ES-API, Pos, Scan, Frag
Max: 79823
[M+H]+= 148 m/z
m/z

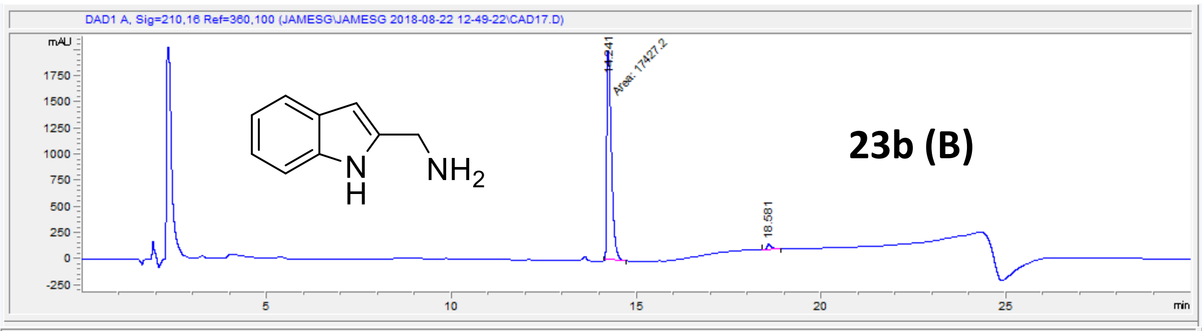
DAD1 A, Sig=210,16 Ref=360,100 (JAMESG\JAMESG 2018-08-22 12-49-22\CAD17.D)
mAU
14.241
Area: 17427.2
18.581
NH2
N
H
23b (B)
5
10
15
20
25
min

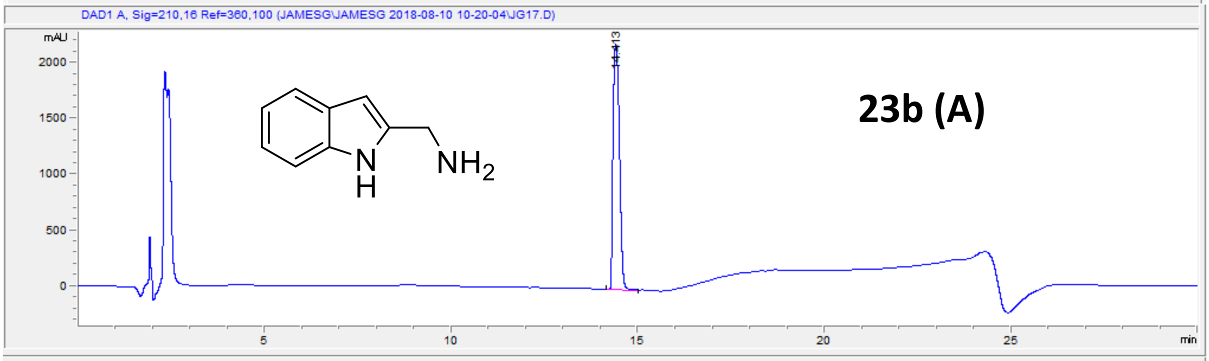
DAD1 A, Sig=210,16 Ref=360,100 (JAMESG\JAMESG 2018-08-10 10-20-04\JG17.D)
mAU
14.413
NH2
N
H
23b (A)
5
10
15
20
25
min

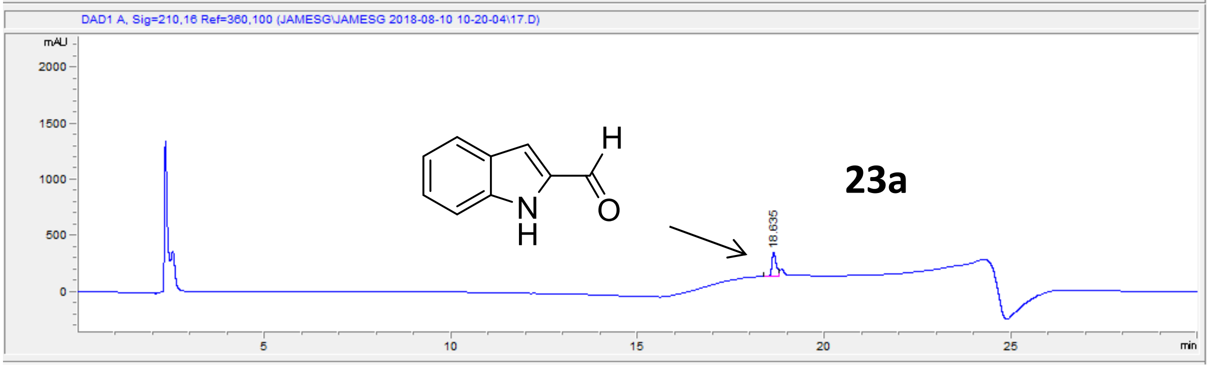
DAD1 A, Sig=210,16 Ref=360,100 (JAMESG\JAMESG 2018-08-10 10-20-04\17.D)
mAU
H
N
H
O
23a
18.635
5
10
15
20
25
min

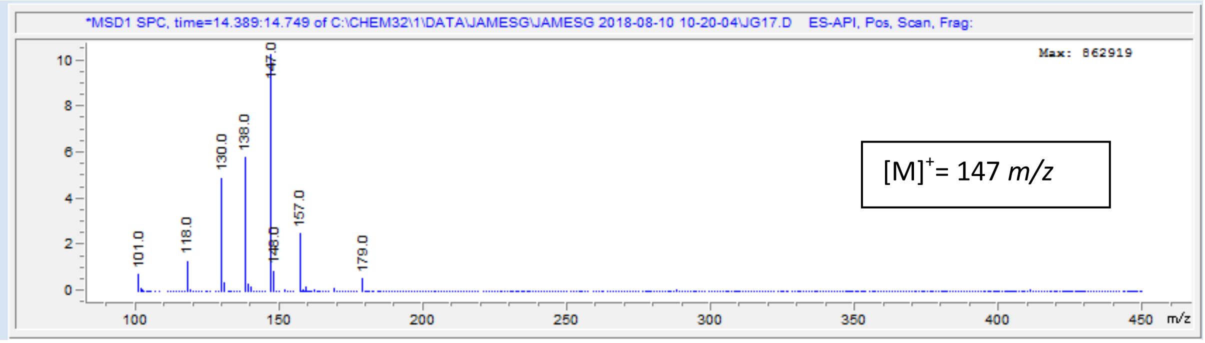
*MSD1 SPC, time=14.389:14.749 of C:\CHEM32\1\DATA\JAMESG\JAMESG 2018-08-10 10-20-04\JG17.D ES-API, Pos, Scan, Frag:
Max: 862919
147.0
138.0
130.0
157.0
118.0
101.0
148.0
179.0
[M]+= 147 m/z
m/z

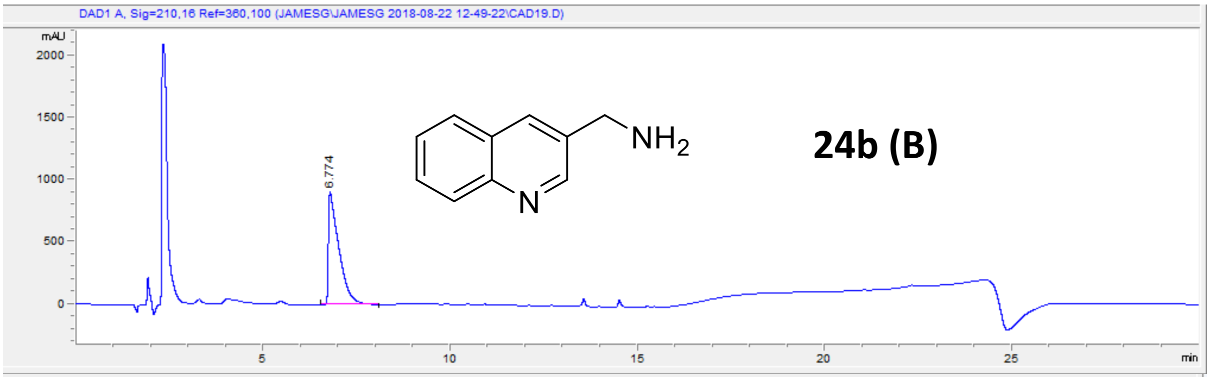
DAD1 A, Sig=210,16 Ref=360,100 (JAMESG\JAMESG 2018-08-22 12-49-22\CAD19.D)
mAU
6.774
NH2
N
24b (B)
min

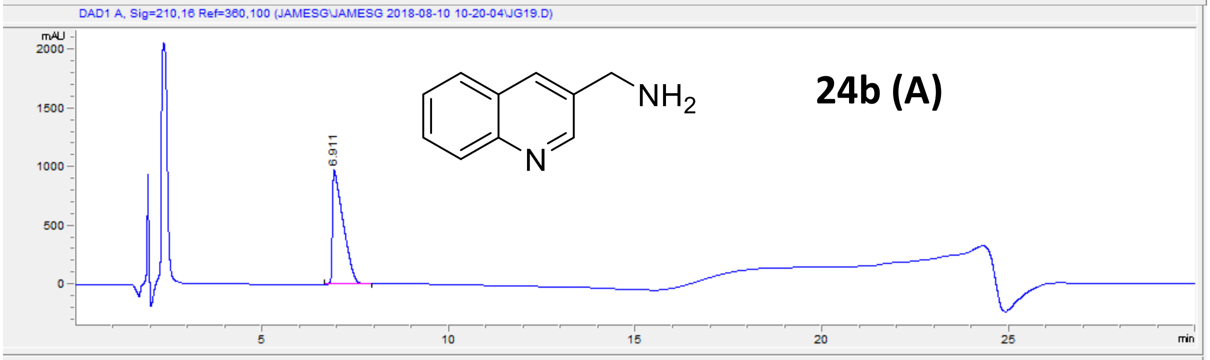
DAD1 A, Sig=210,16 Ref=360,100 (JAMESG\JAMESG 2018-08-10 10-20-04\JG19.D)
mAU
6.911
NH2
N
24b (A)
min

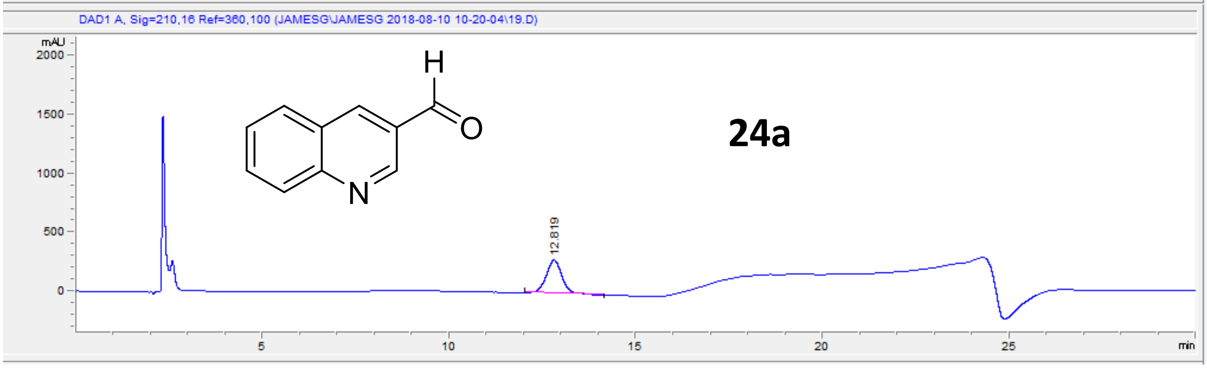
DAD1 A, Sig=210,16 Ref=360,100 (JAMESG\JAMESG 2018-08-10 10-20-04\19.D)
mAU
H
O
N
24a
12.819
min

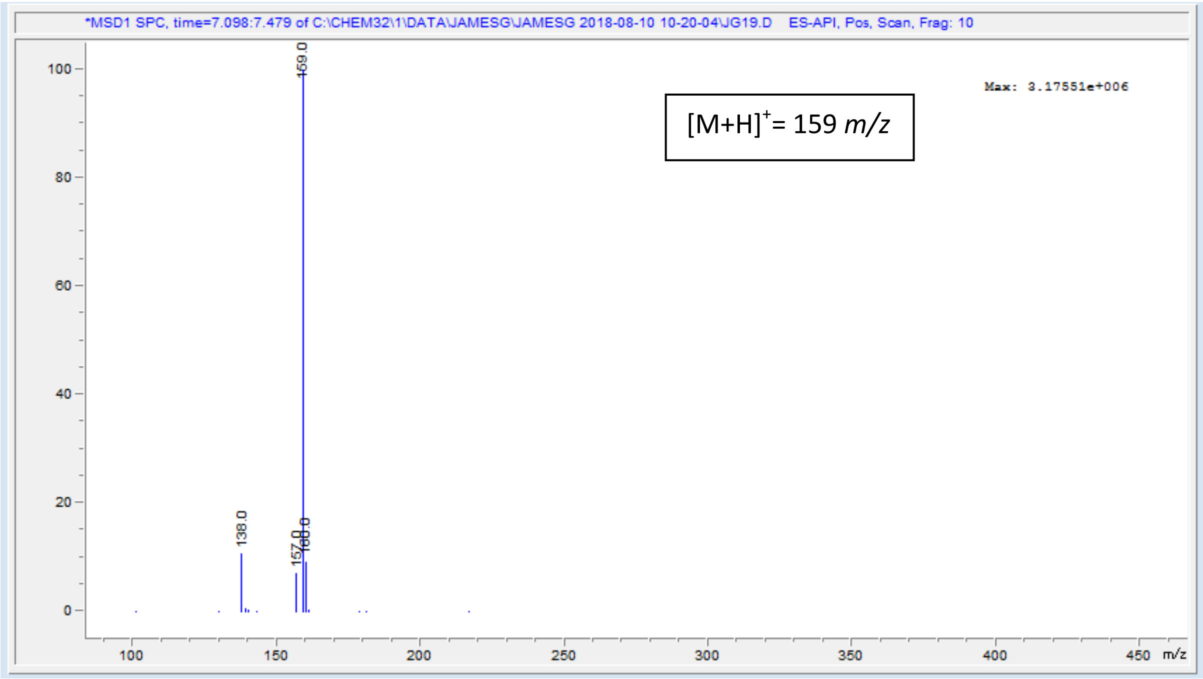
*MSD1 SPC, time=7.098:7.479 of C:\CHEM32\1\DATA\JAMESG\JAMESG 2018-08-10 10-20-04\JG19.D ES-API, Pos, Scan, Frag: 10
Max: 3.17551e+006
[M+H]+= 159 m/z
159.0
138.0
157.0
160.0
m/z

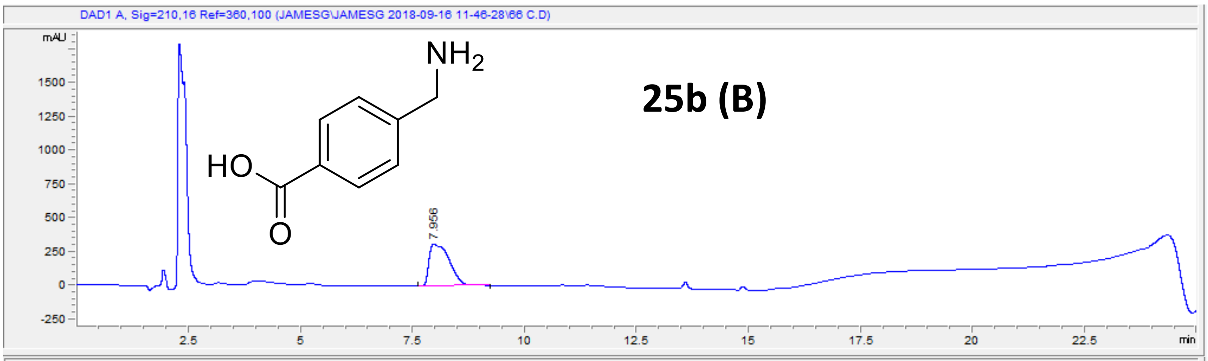
DAD1 A, Sig=210,16 Ref=360,100 (JAMESG\JAMESG 2018-09-16 11-46-28\66 C.D)
mAU
25b (B)
NH2
HO
O
7.956
min

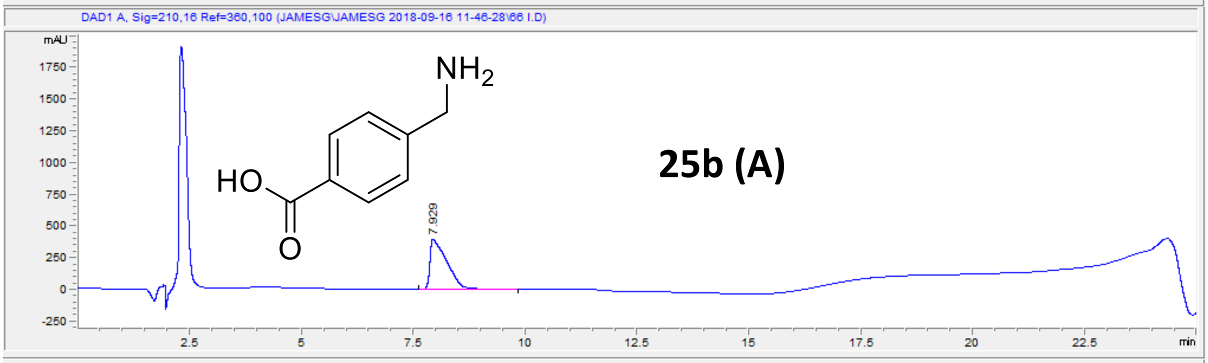
DAD1 A, Sig=210,16 Ref=360,100 (JAMESG\JAMESG 2018-09-16 11-46-28\66 I.D)
mAU
25b (A)
NH2
HO
O
7.929
min

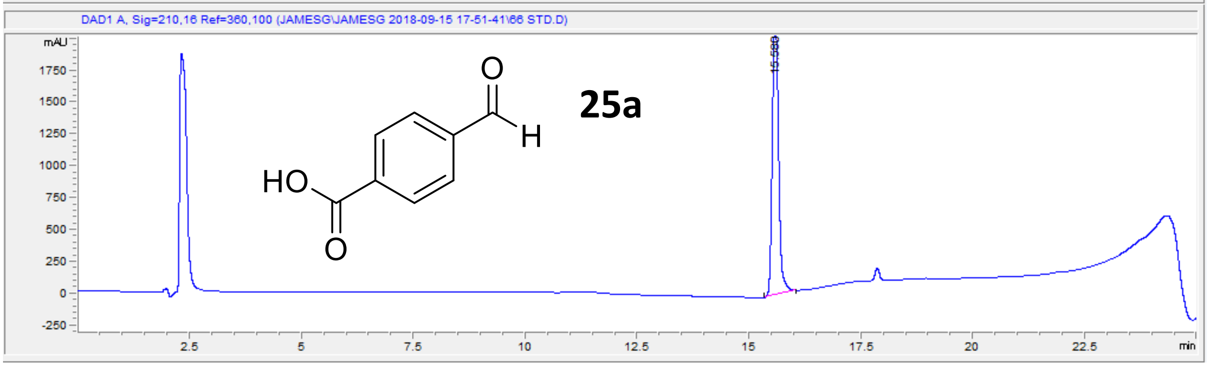
DAD1 A, Sig=210,16 Ref=360,100 (JAMESG\JAMESG 2018-09-15 17-51-41\66 STD.D)
mAU
25a
O
H
HO
O
min

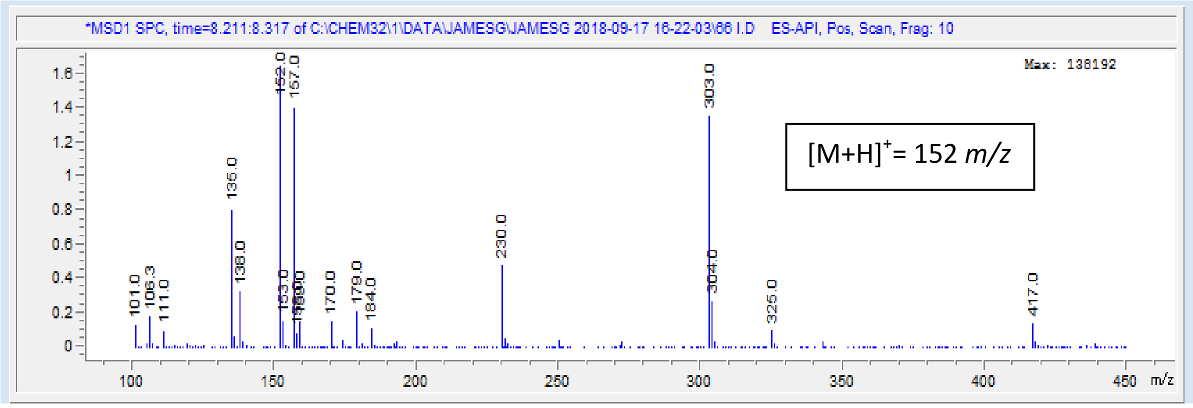
*MSD1 SPC, time=8.211:8.317 of C:\CHEM32\1\DATA\JAMESG\JAMESG 2018-09-17 16-22-03\66 I.D ES-API, Pos, Scan, Frag: 10
Max: 138192
[M+H]+= 152 m/z
152.0
157.0
135.0
138.0
303.0
230.0
101.0
106.3
111.0
170.0
179.0
184.0
304.0
325.0
417.0
m/z

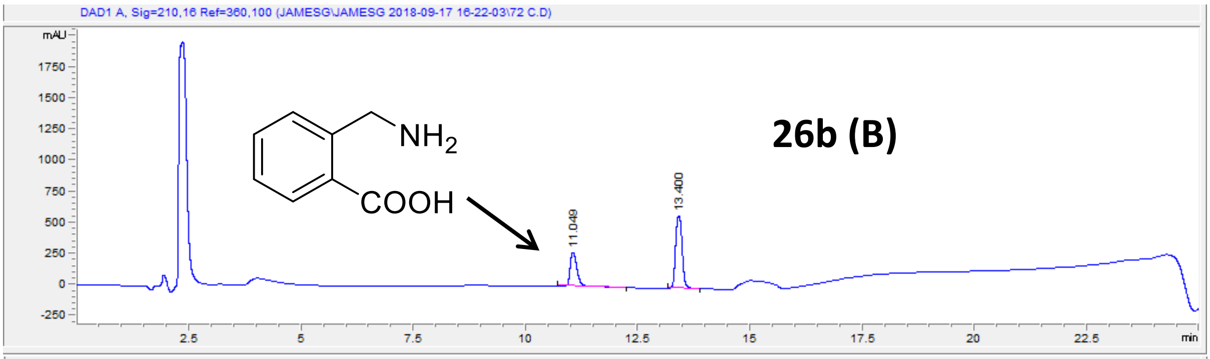
DAD1 A, Sig=210,16 Ref=360,100 (JAMESG\JAMESG 2018-09-17 16-22-03\72 C.D)
26b (B)
NH2
COOH
11.049
13.400
mAU
min

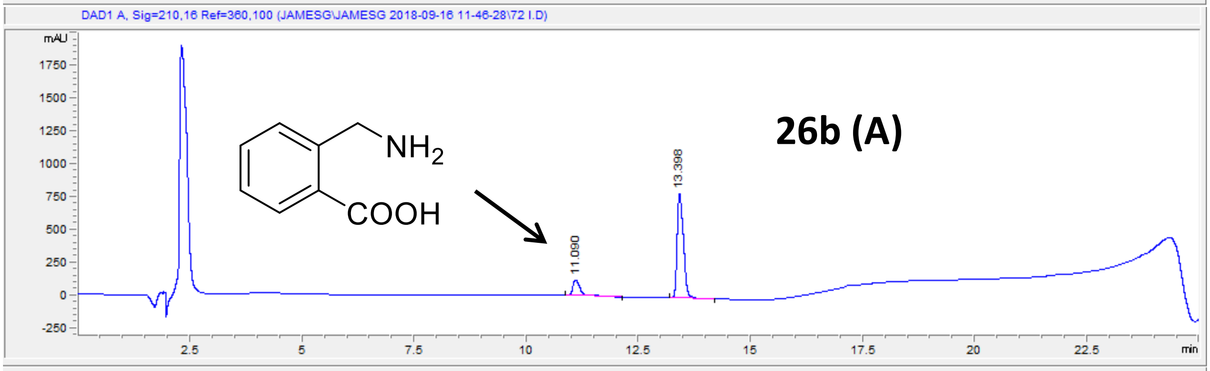
DAD1 A, Sig=210,16 Ref=360,100 (JAMESG\JAMESG 2018-09-16 11-46-28\72 I.D)
26b (A)
NH2
COOH
11.090
13.398
mAU
min

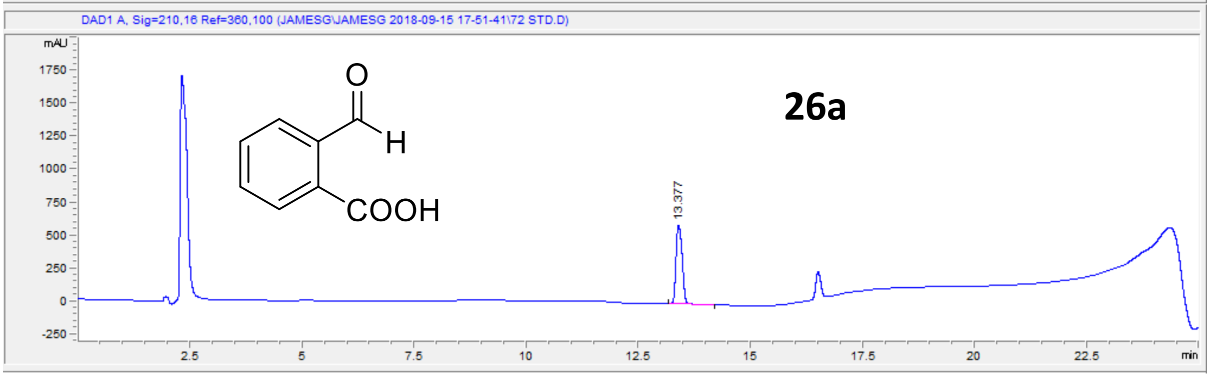
DAD1 A, Sig=210,16 Ref=360,100 (JAMESG\JAMESG 2018-09-15 17-51-41\72 STD.D)
26a
O
H
COOH
13.377
mAU
min

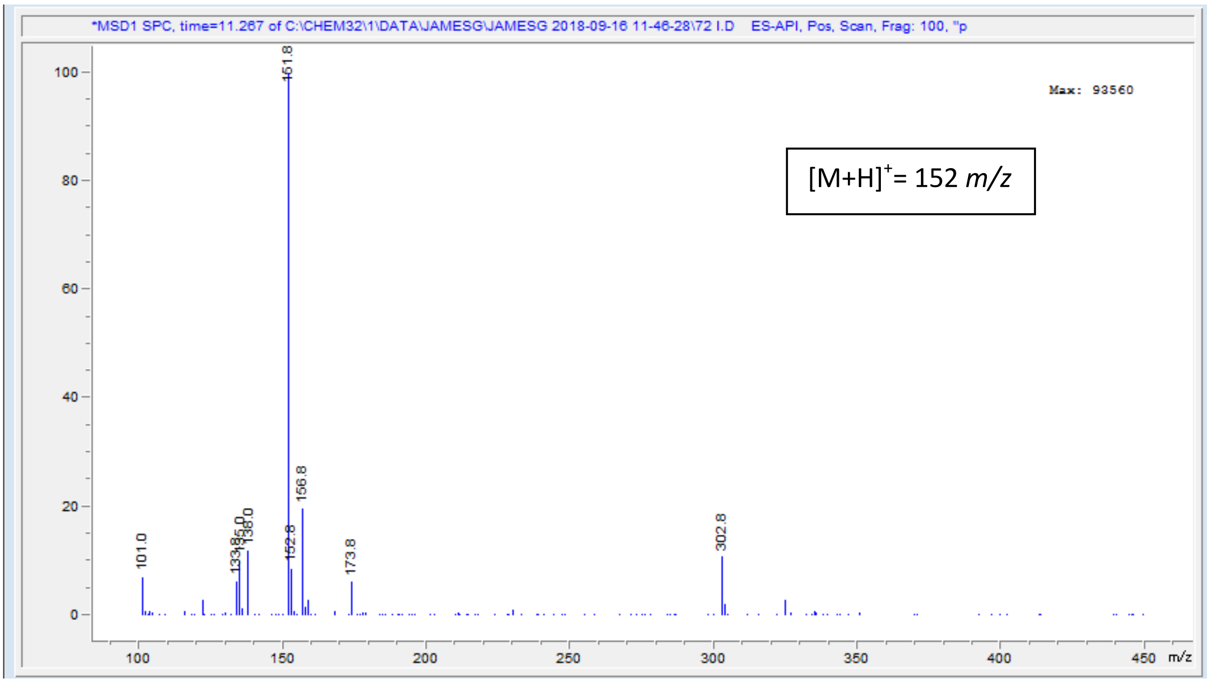
*MSD1 SPC, time=11.267 of C:\CHEM32\1\DATA\JAMESG\JAMESG 2018-09-16 11-46-28\72 I.D ES-API, Pos, Scan, Frag: 100, "p
Max: 93560
[M+H]+= 152 m/z
151.8
156.8
101.0
138.0
152.8
173.8
302.8
m/z

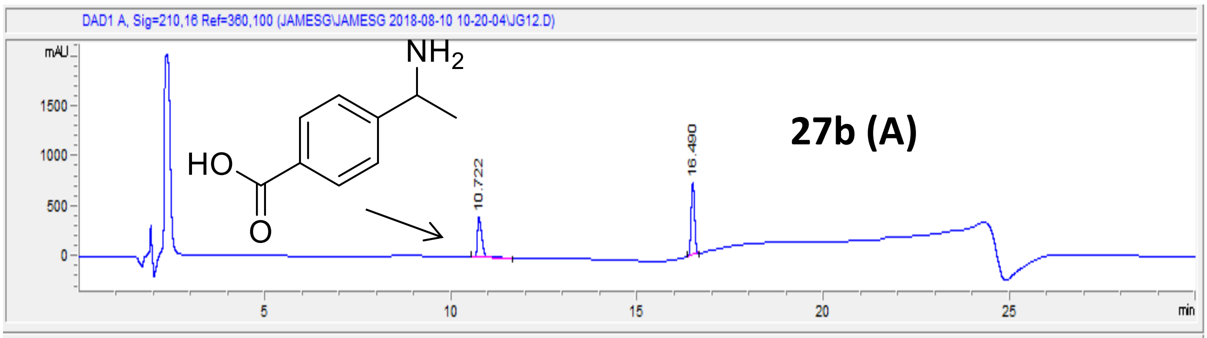
DAD1 A, Sig=210,16 Ref=360,100 (JAMESG\JAMESG 2018-08-10 10-20-04\JG12.D)
27b (A)
10.722
16.490
mAU
min

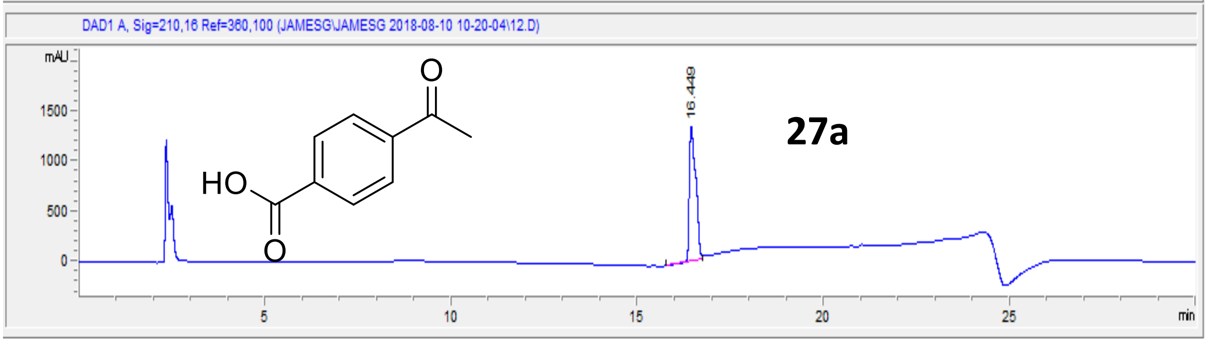
DAD1 A, Sig=210,16 Ref=360,100 (JAMESG\JAMESG 2018-08-10 10-20-04\12.D)
27a
16.449
mAU
min

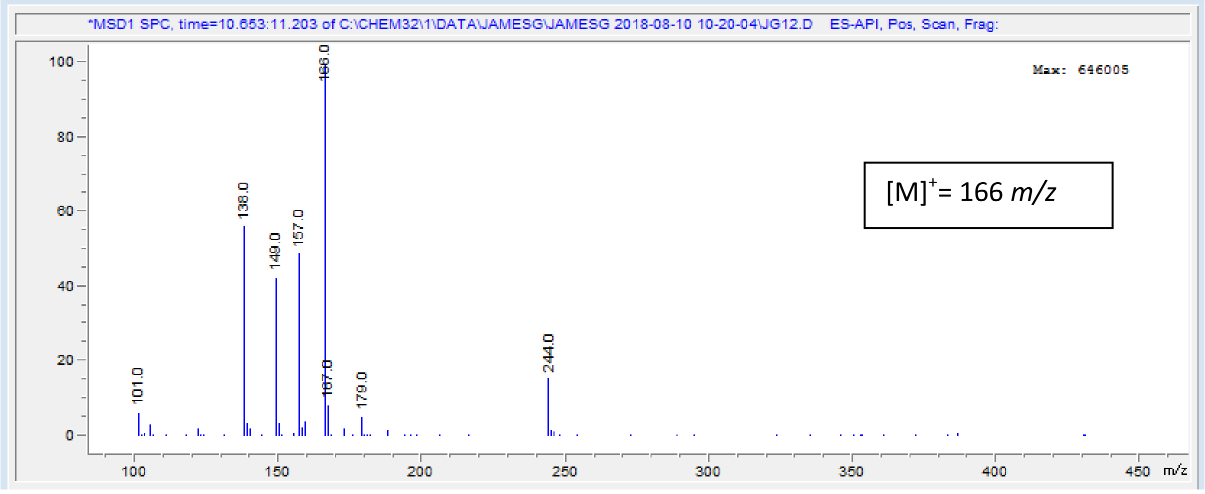
*MSD1 SPC, time=10.653:11.203 of C:\CHEM32\1\DATA\JAMESG\JAMESG 2018-08-10 10-20-04\JG12.D ES-API, Pos, Scan, Frag:
Max: 646005
[M]+= 166 m/z
101.0
138.0
149.0
157.0
166.0
167.0
179.0
244.0
m/z

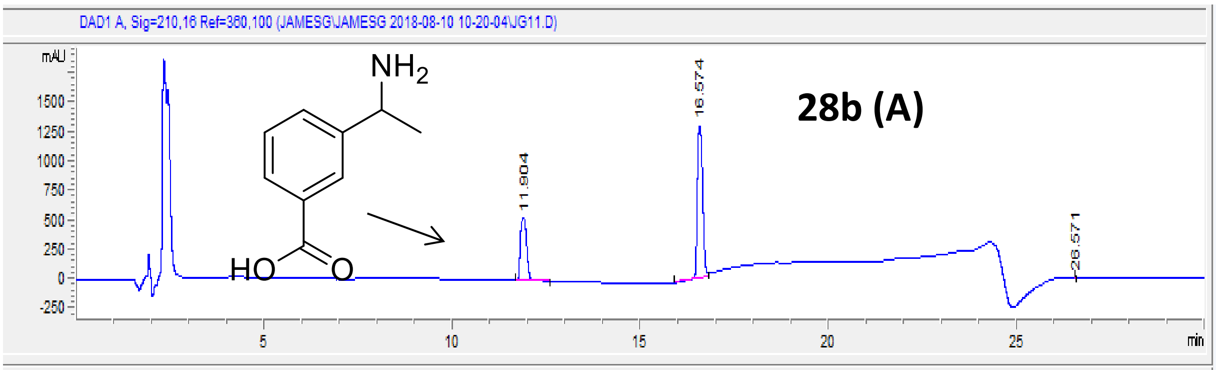
DAD1 A, Sig=210,16 Ref=360,100 (JAMESG\JAMESG 2018-08-10 10-20-04\JG11.D)
mAU
NH2
HO
O
11.904
16.574
26.571
28b (A)
min

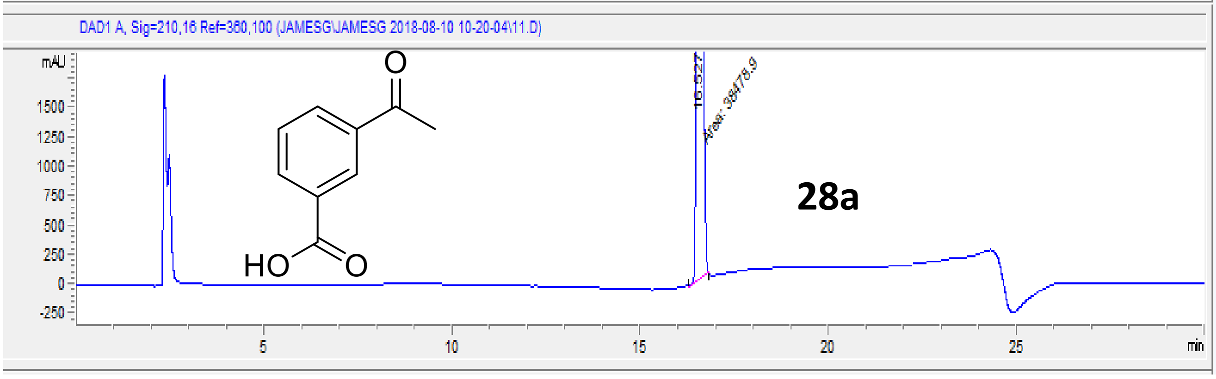
DAD1 A, Sig=210,16 Ref=360,100 (JAMESG\JAMESG 2018-08-10 10-20-04\11.D)
mAU
O
HO
O
28a
min

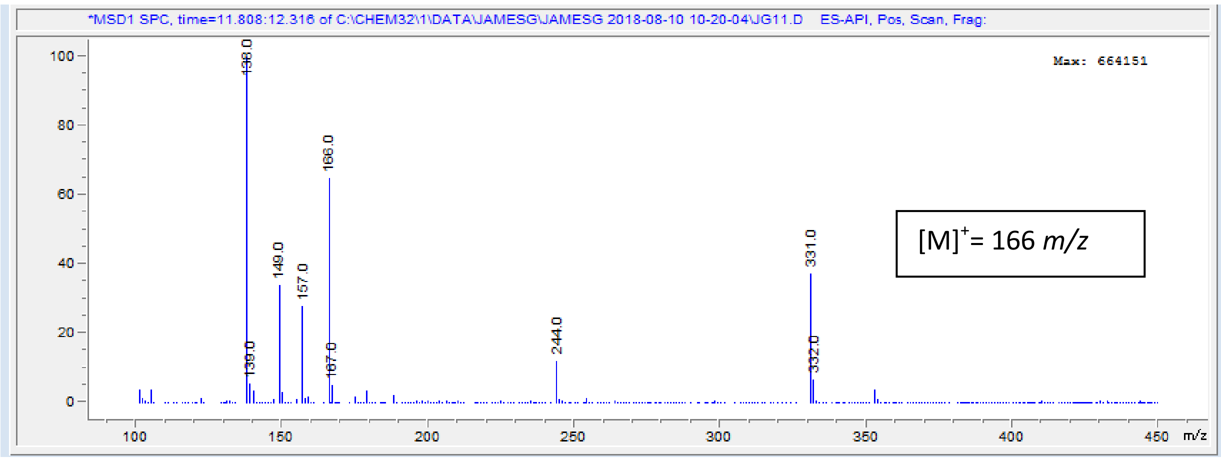
*MSD1 SPC, time=11.808:12.316 of C:\CHEM32\1\DATA\JAMESG\JAMESG 2018-08-10 10-20-04\JG11.D ES-API, Pos, Scan, Frag:
Max: 664151
138.0
149.0
157.0
166.0
244.0
331.0
[M]+= 166 m/z
m/z

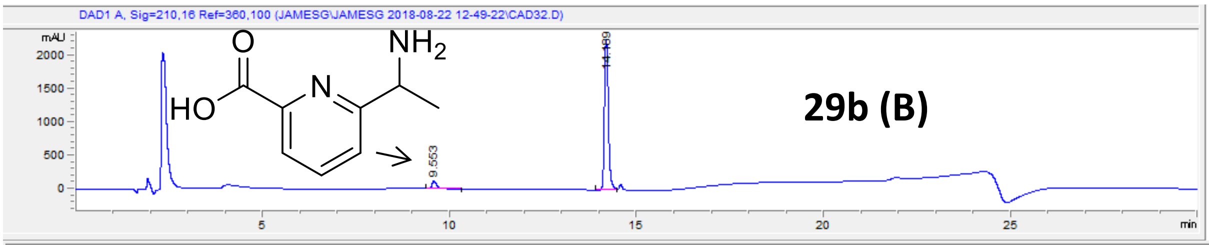
DAD1 A, Sig=210,16 Ref=360,100 (JAMESG\JAMESG 2018-08-22 12-49-22\CAD32.D)
29b (B)
9.553
14.189

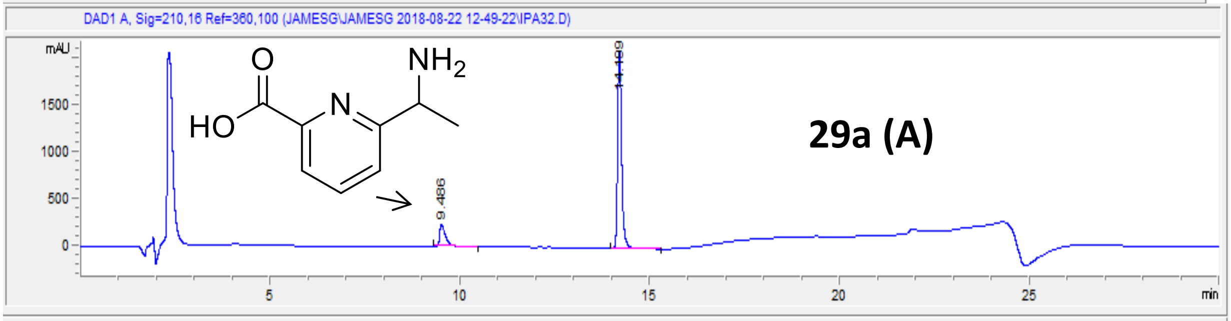
DAD1 A, Sig=210,16 Ref=360,100 (JAMESG\JAMESG 2018-08-22 12-49-22\IPA32.D)
29a (A)
9.486
14.199

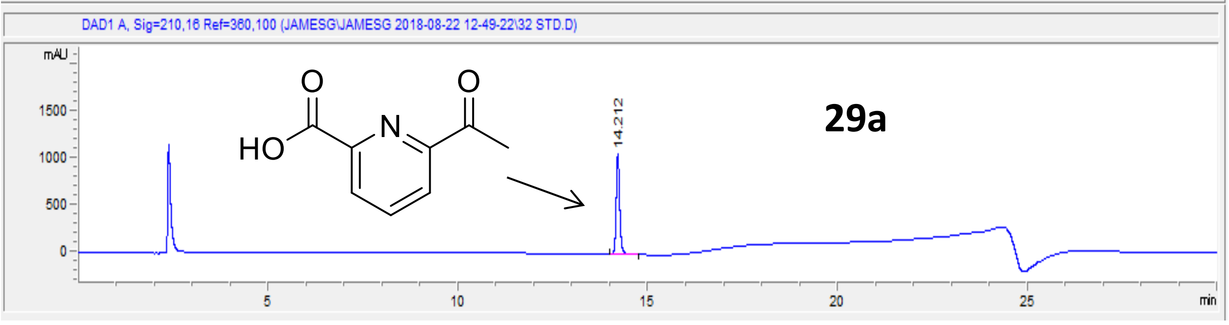
DAD1 A, Sig=210,16 Ref=360,100 (JAMESG\JAMESG 2018-08-22 12-49-22\32 STD.D)
29a
14.212

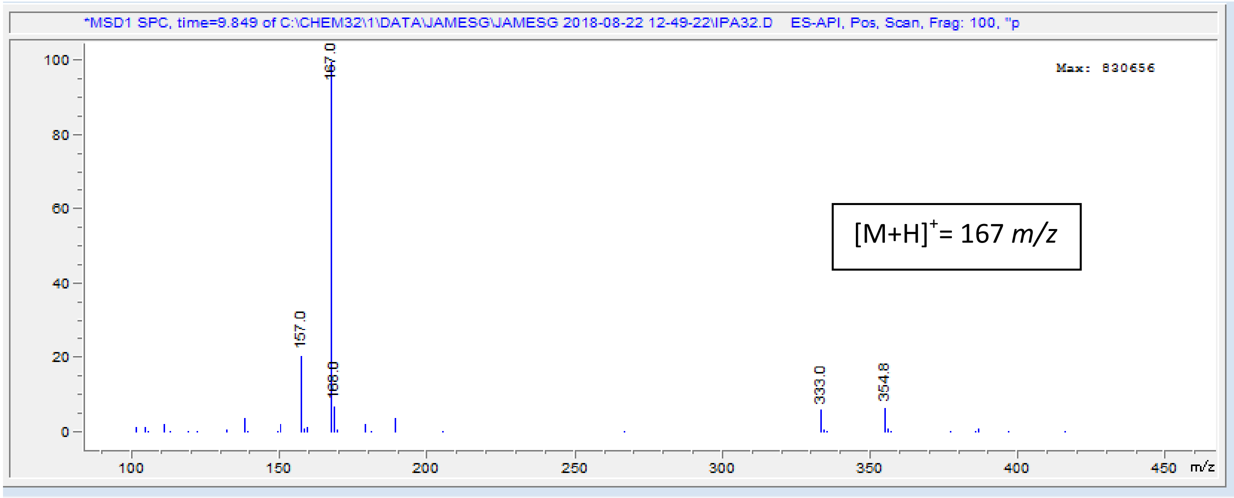
*MSD1 SPC, time=9.849 of C:\CHEM32\1\DATA\JAMESG\JAMESG 2018-08-22 12-49-22\IPA32.D ES-API, Pos, Scan, Frag: 100, "p
Max: 830656
[M+H]+= 167 m/z
167.0
157.0
168.0
333.0
354.8

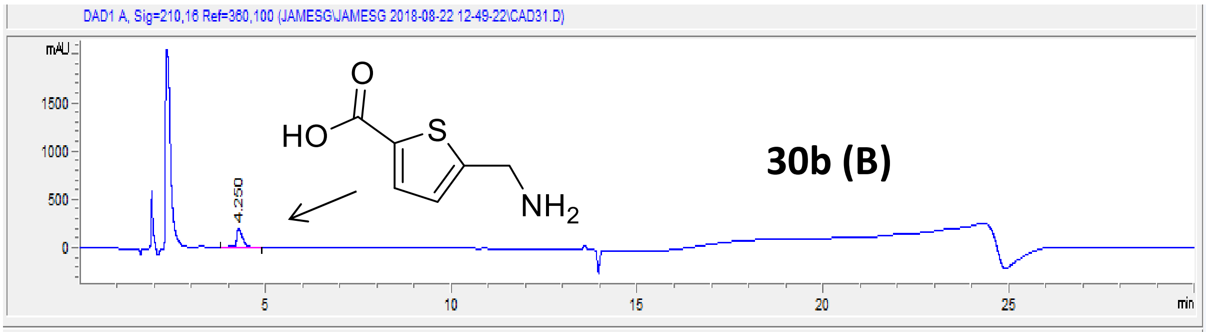
DAD1 A, Sig=210,16 Ref=360,100 (JAMESG\JAMESG 2018-08-22 12-49-22\CAD31.D)
mAU
4.250
HO
O
S
NH2
30b (B)
min

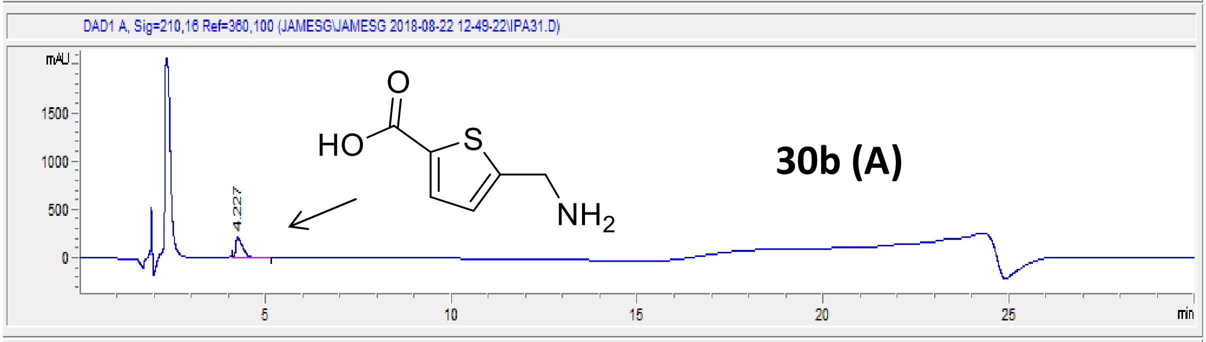
DAD1 A, Sig=210,16 Ref=360,100 (JAMESG\JAMESG 2018-08-22 12-49-22\IPA31.D)
mAU
4.227
HO
O
S
NH2
30b (A)
min

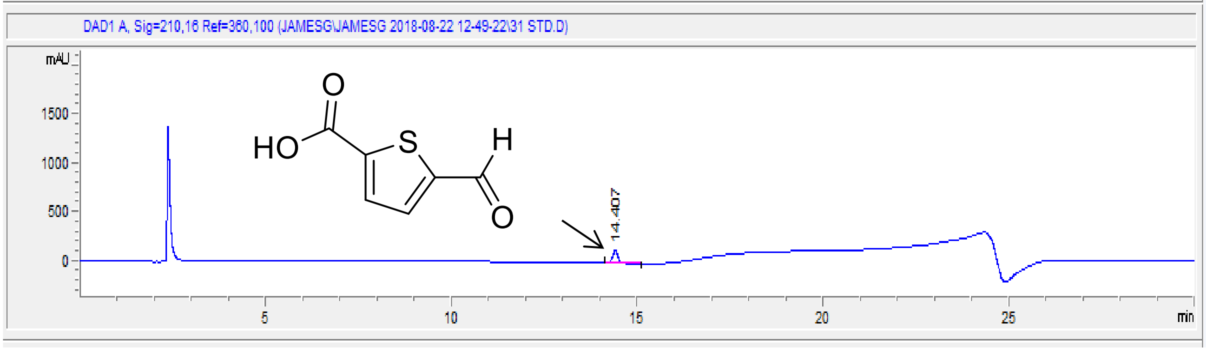
DAD1 A, Sig=210,16 Ref=360,100 (JAMESG\JAMESG 2018-08-22 12-49-22\31 STD.D)
mAU
HO
O
S
H
14.407
min

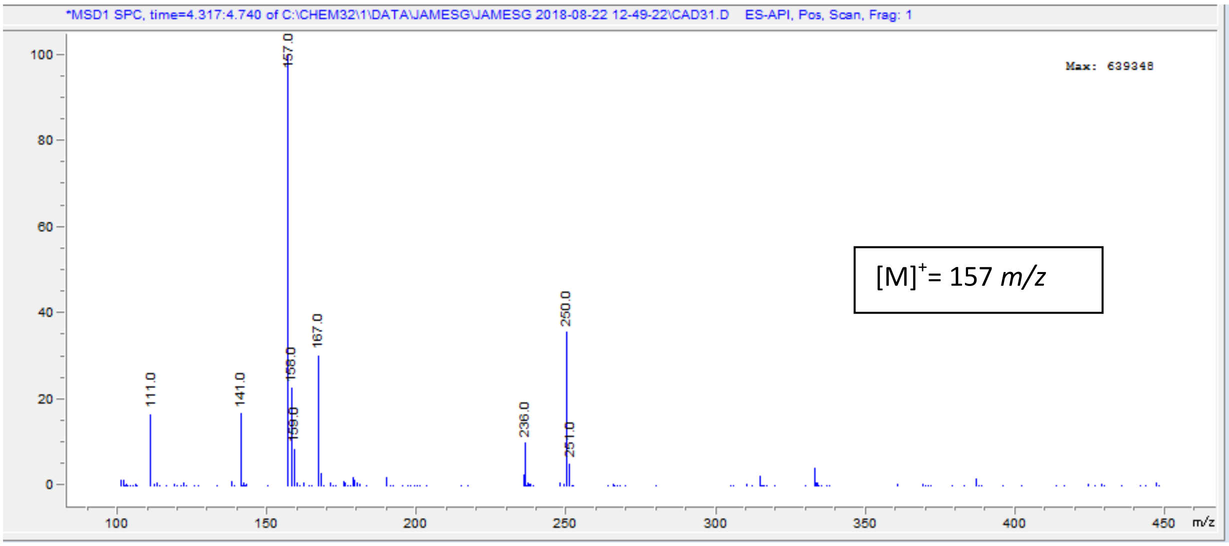
*MSD1 SPC, time=4.317:4.740 of C:\CHEM32\1\DATA\JAMESG\JAMESG 2018-08-22 12-49-22\CAD31.D ES-API, Pos, Scan, Frag: 1
Max: 639348
157.0
111.0
141.0
158.0
167.0
236.0
250.0
251.0
[M]+= 157 m/z
m/z
